# NF-κB and AP-1 are required for the lipopolysaccharide-induced expression of MCP-1, CXCL1, and Cx43 in cultured rat dorsal spinal cord astrocytes

**DOI:** 10.3389/fnmol.2022.859558

**Published:** 2022-07-28

**Authors:** Ying Lu, Bo Li, Axiang Xu, Xuan Liang, Tao Xu, Huan Jin, Ye Xie, Rong Wang, Xiaohong Liu, Xiaohong Gao, Yong Han, Junwei Zeng

**Affiliations:** Department of Physiology, Zunyi Medical University, Zunyi, China

**Keywords:** astrocyte, NF-κB, AP-1, Cx43, MCP-1, CXCL1

## Abstract

TLR4 and Cx43 signaling in dorsal spinal cord has been shown to be involved in the development of neuropathic pain. However, it is not clear whether TLR4 signaling is associated with the expression of MCP-1, CXCL1, and Cx43 in LPS (lipopolysaccharide)-treated rat dorsal spinal cord astrocytes under *in vitro* condition. In the present study, we found that TLR4 antagonist TAK-242 significantly inhibited LPS-induced MCP-1, CXCL1, and Cx43 expression, suggesting the role of TLR4 in response to LPS in cultured dorsal spinal cord astrocytes. Application of TAK-242 significantly blocked LPS-induced NF-κB and AP-1 activity and the expression of MCP-1, CXCL1 and Cx43. Furthermore, NF-κB inhibitor PDTC and AP-1 inhibitor SR11302 significantly blocked LPS-induced MCP-1, CXCL1, and Cx43 expression. DNA-binding activity of NF-κB, its effect on MCP-1 expression was suppressed by PDTC and SR11302. On the other hand, DNA-binding activity of AP-1, its effect on CXCL1 or Cx43 expression was also suppressed by PDTC and SR11302. In addition, PDTC was found to inhibit the nuclear translocation of AP-1 and the expression of c-Jun induced by LPS, which suggested that NF-κBp65 is essential for the AP-1 activity. Similarly, SR11302 significantly blocked LPS-induced the nuclear translocation of NF-κBp65 and the expression of NF-κBp65 induced by LPS. Pretreatment with CBX, Gap26, or Gap19 (Cx43 blockers) significantly inhibited abnormal astrocytic hemichannel opening and chemokines (MCP-1 and CXCL1) release in LPS-stimulated astrocytes. In summary, cell culture experiments revealed that LPS stimulation could evoke TLR4 signaling with the subsequent activation of NF-κB and AP-1, resulting in the expression of MCP-1, CXCL1, and Cx43. TLR4 activation increased Cx43 hemichannel, but not gap-junction activities and induced the release of the MCP-1 and CXCL1 from astrocytes via Cx43 hemichannel. These findings may help us to understand the role of astrocytic signaling in inflammatory response within dorsal spinal cord tissue.

## Introduction

It is clear that toll-like receptors (TLRs) are type I transmembrane glycoproteins that play a crucial role in modulating innate immune responses against invading pathogens. Among these receptors, TLR4 can be activated by lipopolysaccharide (LPS, a component of the cell wall of gram-negative bacteria) to activate nuclear factor-κB (NF-κB) and activator protein 1 (AP-1) pathways. TLR4 is widely expressed in microglia, astrocytes, and macrophages in nervous system and may participate in the pathogenesis of neurological diseases through inducing inflammatory response (Abg Abd Wahab et al., 2019; Muhammad et al., 2019). It is reported that TLR4 triggers the LPS-induced inflammatory responses through activating transcription factors NF-κB and AP-1 in BV-2 cells, rodent brain astrocyte and U373MG human astrocytoma cells (Lu et al., 2010; Gorina et al., 2011; Wang et al., 2011; Imaizumi et al., 2013; Yang et al., 2020). Recent studies have suggested that TLR4, macrophage chemoattractant protein-1 (MCP-1) and chemokine (C-X-C motif) ligand 1 (CXCL1) are expressed in spinal astrocytes and are involved in nociceptive transmission at the spinal cord level. In addition, NF-κB-mediated MCP-1 production in mouse spinal astrocytes was reported by Zhao et al. (2014). Furthermore, treatment with NF-κB inhibitor can affect LPS-induced expression of MCP-1 and CXCL1 in rat cerebral cortex astrocytes (Liu et al., 2018). On the other hand, *in vivo* experiment, immunofluorescence double staining showed that TLR4 is predominantly expressed in spinal astrocytes (Qian et al., 2016). However, it is not clear whether both NF-κB and AP-1 are required for the expression of MCP-1 and CXCL1 in cultured LPS-stimulated dorsal spinal cord astrocytes.

Adjacent astrocytes form gap junction channels which are composed of opposing hexamers of connexins (Cxs). Cx43 is the principal connexin expressed by astrocytes in central nervous system (Bennett et al., 2012). In mouse hippocampal astrocytes, LPS-induced Cx43 hemichannel opening is related to Ca^2+^ mobilization and glutamate release (Abudara et al., 2015). Strikingly, spinal cord injury-induced inflammatory cytokines release and neuropathic pain was abolished in Cx43/Cx30 double knockout mice, but not in Cx30 knockout mice, suggesting the crucial role of Cx43 hemichannel in the development and maintenance of pathological pain (Chen et al., 2012). In cultured mouse spinal astrocytes, TNF-α elicited marked release of the CXCL1, and the release was blocked by Cx43 inhibitor (Chen et al., 2014). However, it is not clear whether the Cx43 expression is associated with transcription factors NF-κB and AP-1 in cultured astrocytes.

Recent evidences have suggested that NF-κBp65 and AP-1 can bind to the promoters of the MCP-1, CXCL1, and Cx43 genes and regulate their transcriptions (Teunissen et al., 2003; Gao et al., 2007; Sutcliffe et al., 2009; Panicker et al., 2010; Chen et al., 2012, 2014; Burke et al., 2014; Lennard Richard et al., 2015; Deng et al., 2017; Angeli et al., 2020). The MCP-1 promoter construct includes binding sites for AP-1 and NF-κB in human airway smooth muscle cells (Sutcliffe et al., 2009). The binding of NF-κB to the MCP-1 promoter was detected in human lung fibroblasts cells (Deng et al., 2017). However, in mouse kidney endothelial cells, NF-κBp65 was found to directly activate transcription of MCP-1, while p50 inhibit the interaction (Lennard Richard et al., 2015). It seems that p65 and p50 subunit participate in MCP-1 transcription. Even more, NF-κBp65 and AP-1 are involved in high glucose-evoked MCP-1 gene expression in rat aortic endothelial cells (Panicker et al., 2010). In addition, IL-1β-induced NF-κB activity is required for CXCL1 transcription in rat islets and β-cell lines (Burke et al., 2014). Similarly, the Cx43 promoter has AP-1 binding sites in rat cardiomyocytes (Teunissen et al., 2003). It is reported that AP-1 site exert an important role in Cx43 expression in human leukemic cell lines (Gao et al., 2007). For this reason, in cultured rat dorsal spinal cord astrocytes, it is interesting to explore whether both NF-κB and AP-1 are required for LPS-induced gene expression of MCP-1, CXCL1, and Cx43.

## Materials and methods

The study was not pre-registered prior to examination of the data or observing the outcomes.

### Animals

Adult male and female Sprague-Dawley rats (250–280 g) were obtained from the Experimental Animal Center of Zunyi Medical University, which were breeding and mated in the animal facility of the Department of Physiology in Zunyi Medical University (Zunyi, China). Rats were housed in groups of two per cage in sanitary barrier room in ventilated racks with free access to food and tap water. The room temperature was maintained at 22°C with a 12 h light/dark cycle (lights on/off at 8:00 AM/8:00 PM). Primary dissociated cultures of dorsal spinal cord were prepared from SD rats (≤3 days) in accordance with the National Institutes of Health guidelines in a manner that minimized animal suffering and animal numbers. All experiments were carried out in accordance with China animal welfare legislation and were approved by the Zunyi Medical University Committee on Ethics in the Care and Use of Laboratory Animals.

### Purification and culture of astrocytes

Primary dissociated cultures of dorsal spinal cord astrocytes were prepared and maintained as described previously (Zeng et al., 2008). No sample calculation was performed to predetermine the sample size. For the experiments of this work, we used 8 litters of 12 pups each of both sexes. Briefly, SD rat pups (≤3 days) were anesthesia with ether vapors, killed by rapid decapitation using sterile scissors, and euthanasia was performed between 8 am to 12 noon to minimize animal suffering. This method of anesthesia was chosen because others common methods (such as isoflurane) were found to decrease primary astrocytic viability (Guo et al., 2017). Their dorsal spinal cord was rapidly cut into pieces and then incubated in 0.125% trypsin (HyClone, SH30042.01) for 30 min at 37 °C. Dulbecco’s modified Eagle’s medium (DMEM/F12 medium, Gibco, C11330500BT) containing 15% (v/v) fetal bovine serum (FBS, MRC, CCS30009.02) was added to stop the action of typsin. The cells were collected after centrifugation at 1,000 *g* min for 5 min. The supernatants were discarded and the cells were suspended in complete medium containing DMEM/F12 and 15% (v/v) fetal bovine serum. 10^5^ cells/cm^2^ cells were planted in poly-D-lysine-coated 75 cm^2^ flask and cultured in the incubator at 37°C in a humidified 5% CO_2_-95% air atmosphere. After 10 days, flasks were shaken (260 rpm/min) overnight to remove non-astroglial cells. Dorsal spinal cord astrocytes stained positively for the astrocytic marker, glial fibrillary acid protein (GFAP).

### Drug application

Lipopolysaccharide (L2630), TAK-242 (TLR4 antagonist; 614316), carbenoxolone (CBX, a Cx43 hemichannel and gap junction inhibitor; C4790) (Yin et al., 2018), Gap19 (inhibiting Cx43 hemichannel activity but not gap junctional communication; SML1426) (Yin et al., 2018), ammonium pyrrolidinedithiocarbamate (PDTC, NF-κBp65 inhibitor; 548000), ethidium bromide (EtBr; E8751) and lucifer yellow (LY; L0144) were purchased from Sigma (St. Louis, MO, United States). Gap26 (a specific gap junction and hemichannel blocker corresponding to residues 63–75 of Cx43; AS-62644) (Yin et al., 2018) was purchased from AnaSpec. SR11302 (AP-1 inhibitor; sc-204295) was purchased from Santa Cruz, Dallas, TX, United States. Among these drugs, LPS, CBX, Gap26, Gap19, PDTC, SR11302, EtBr and LY were dissolved in 0.01M PBS. CBX is a widely used Cx43 gap junction/hemichannel blocker (Ye et al., 2009). TAK-242 was dissolved in 10% dimethyl sulfoxide (DMSO) and diluted in 0.01M PBS. Vehicle (0.01% DMSO diluted in PBS solution) was used in the Veh-treated group and Veh + LPS group. The presence of DMSO (<0.01%) alone did not affect the production and release of chemokine from cultured astrocyte at quiescence state.

**FIGURE 1:**
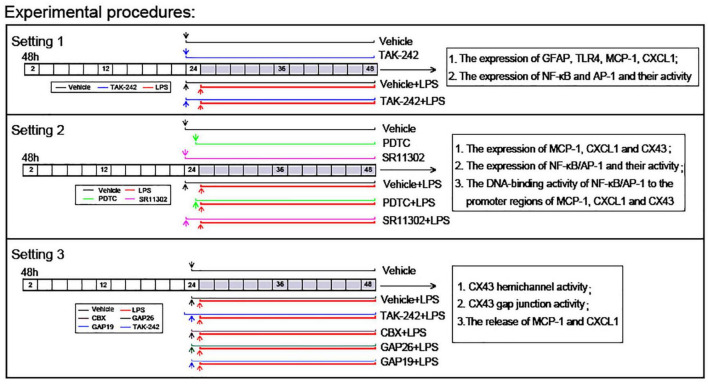
Experimental design *in vitro*.

According to previous studies, LPS at dose of 1 μg/ml for 24 h induced astrocytic activation and inflammatory cytokine production (Oliveira-Junior et al., 2019). However, LPS at dose of 2 μg/ml was shown to remarkable decrease cell viability in primary cultured astrocytes (Xian et al., 2019). For this reason, we used 1μg/mL LPS for 24 h in the following experiments. In addition, TAK-242 at 100 nM was used in order to confirm the role of TLR4 in LPS-induced inflammatory response in cultured astrocytes (Du et al., 2017). It is reported that CBX, Gap26, and Gap19 at 100 μM were frequently used in order to establish the involvement of Cx43 in neuroactive substance release from astrocytes (Takaki et al., 2012; Guillebaud et al., 2020). PDTC at 1 μM and SR11302 at 10 μM were frequently used in order to establish the involvement of NF-κBp65 and AP-1 signaling in cellular responses (Zhao et al., 2015; Liddell et al., 2016). Based on these previous experimental reports, in our present experiments, cells were exposed to LPS (1 μg/ml for 24 h) with or without pretreatment of TAK-242 (100 nM for 2 h), PDTC (1 μM for 30 min), SR11302 (10 μM for 2 h), CBX (100 μM for 1 h), Gap26 (100 μM for 1 h), and Gap19 (100 μM for 1 h), respectively. Afterward, the cells and supernatants were harvested separately and processed according to the different assay protocols (Figure 1).

### Immunofluorescence

To observe the nuclear translocation of NF-κB or AP-1 in astrocytes, cells grown on glass coverslips were stained for NF-κBp65/p50, AP-1 and DAPI. After treatment with 1 μg/ml LPS for 24 h, the cells were fixed in 4% paraformaldehyde for 20 min, washed three times with 0.01 M PBS and incubated with 10% goat serum for 15 min to block non-specific binding. Astrocytes cultures were stained with rabbit anti-NF-κBp65 antibody (520 μg/ml, 1:350, Proteintech Group, Rosemont, IL, United States), mouse anti-NF-κBp50 antibody (200 μg/ml, 1:350, Santa Cruz, Dallas, TX, United States) or rabbit anti-AP-1 antibody (1200 μg/ml, 1:350, Proteintech Group, Rosemont, IL, United States) overnight. Subsequently, the cultures were incubated with FITC-conjugated goat anti-rabbit IgG (600 μg/ml, 1:350 in PBS, Proteintech Group, Rosemont, IL, United States) or FITC-conjugated goat anti-mouse IgG (600 μg/ml, 1:350 in PBS, Proteintech Group, Rosemont, IL, United States) for 1 h at room temperature. Finally, 4’,6-diamidino-2-phenylindole (DAPI, 1:1000, Roche) was applied for 5 min to label all nuclei. We randomly chose 10 visual fields (nearly 50 cells per visual field) to count the percentage of the positive cells (nuclear staining for NF-κBp65/p50 or AP-1) with Image J software.

In addition, cells grown on glass coverslips were stained for TLR4 and Cx43 by double immunofluorescence. Astroglial cultures were stained with mouse anti-TLR4 antibody (1700 μg/ml, 1:350, Proteintech Group, Rosemont, IL, United States) overnight. Subsequently, the cultures were incubated with CY3-conjugated goat anti-mouse IgG (600 μg/ml, 1:350 in PBS, Proteintech Group, Rosemont, IL, United States) for 1 h, followed by incubations with rabbit anti-Cx43 antibody (700 μg/ml, 1:350, Proteintech Group, Rosemont, IL, United States) overnight. Then, the cultures were incubated with FITC-conjugated goat anti-rabbit IgG (600 μg/ml, 1:350 in PBS, Proteintech Group, Rosemont, IL, United States) for 1 h. At last, DAPI was applied for 5 min to label all nuclei. All incubations were held at 25°C and separated by three 5-min washes in 0.01 M PBS in a dark place. Control experiments were performed using an excess of the appropriate homolog peptide antigen to absorb the primary antibodies and thus confirm a specific immunoreaction. After staining, coverslips were mounted on glass slides and examined using Airyscan2 confocal laser scanning microscope 900 (Zeiss, Germany).

### Real time quantitative PCR

The total RNA of cultured dorsal spinal cord astrocytes was isolated by using Trizol reagent after 24 h of drug administration. Real time quantitative PCR (RT-qPCR) is carried out in iCycler IQ Real-Time PCR Detection System (BIO-RAD Co., Pleasanton, CA, United States) with SYBR Green PCR Master Mix (ABI Co., Foster, CA, United States). PCR conditions were as follows: 95°C 30 s 1 Cycle; 95°C 5 s, 60°C 30 s, 40 Cycles. The cycle threshold (Ct) value represents the cycle number at which a fluorescent signal rises statistically above background. The relative quantification of gene expression was analyzed by the 2^–Δ^ ^Δ^ Ct method.

The nucleotide sequences of the primers were synthesized by TaKaRa Biological Engineering Company. Primer preparations were devised according to the sequence searched on GeneBank. The nucleotide sequences of the primers used in this experiment were as follows: (1) MCP-1 (genebank: NC-005109.4): F: 5′-CAGGTC TCTGTCACGCTTC-3′; R: 5′-AGTTCTCCAGCCGACTCA-3′. (2) CXCL1 (genebank: NC-005113.4): F: 5′-AACCGAAGTCATAGCCACA-3′; R: 5′-GGGGA CACCCCTTTAGCA-3′; (3) Cx43 (genebank: NC-051355.1): F: 5′-ATCATCC TCGACTGGTCCT-3′; R: 5′-ACATCCACACGTAGAAGC-3′. (4) NF-κBp65 (genebank: NC-051336.1): F: 5′-CCAAAGACCCACCTCACC-3′; R: 5′-CGCTTCTTCACACACTGGA-3′; (5) NF-κBp50 (genebank: NC-051337.1): F: 5′-GGGCAGAAGTCAACG-3′; R: 5′-TGTCGTCCCATCGTAGGT-3′; (6) c-Jun (genebank: NC-051340.1): F: 5′-TGTCTGTATGCTGGGGTGA-3′; R: 5′-GGTTGCTGGGGAGAGAGA-3′; (7) β-actin (genebank: NC-005111.4): F: 5′GGCTGTATTCCCCTCCATCG-3′; R: 5′-CCAGTTG GTAACAATGCCATGT-3′.

### Western blot

Total proteins were extracted from cultured astrocytes lysed by radioimmunoprecipitation (RIPA) buffer containing protease and phosphatase inhibitor cocktails (Solarbio Science & Technology Co., Ltd, Beijing, China), and the protein concentration was determined by using a BCA protein assay kit (Solarbio Science & Technology Co., Ltd, Beijing, China). Then 20 μg equal amounts of denatured proteins were subjected to SDS-PAGE gel electrophoresis and transferred onto polyvinylidenefluoride (PVDF) membranes (Merck Millipore Ltd, Cork, Ireland). Next, the transferred membranes were blocked with 5% Albumin Bovine V (Solarbio, A8020) for 2 h and incubated with the primary antibodies (Table 1) overnight at 4°C. The following day, the membranes were washed three times with 0.1% Tween-Tris-buffered saline (TBST, Servicebio, G0001), and then incubated with secondary antibodies (Table 1) at room temperature for 1 h. Finally, the membranes were imaged using an Electro-Chemi-Luminescence-Kit. Images of the blots were captured using the G: BOX Chemi XX9 system (Synoptics Group, Frederick, MD, United States). The image was scanned, and band intensity was semi-quantified using Image J (version 1.8.0, National Institutes of Health). β-actin or glyceraldehyde 3-phosphate dehydrogenase (GAPDH) was used as the internal reference, and the gray value of the target protein was divided by the gray value of the internal reference to correct the error. The result represents the relative content of the target protein in the sample. The protein levels were normalized to β-actin or GAPDH protein levels and the results are shown as the percentage of Vehicle (% of Vehicle).

**TABLE 1:** List of antibodies and their information used in this study.

Antibody	Catalog	RRID	Application (Conc.)	Host	Manufacturer
Anti-TLR4	66350-1-Ig	RRID:AB_2881730	WB/IF (1:1000/1:350)	Mouse	Proteintech Group		
Anti-GFAP	16825-1-AP	RRID:AB_2109646	WB/IF (1:2000/1:350)	Rabbit	Proteintech Group		
Anti-MCP-1	66272-1-Ig	RRID:AB_2861337	WB (1:1000)	Mouse	Proteintech Group		
Anti-CXCL1	AF5403	RRID:AB_2837887	WB (1:1000)	Rabbit	Affinity		
Anti-NF-κBp65	10745-1-AP	RRID:AB_2178878	WB/IF (1:1000/1:350)	Rabbit	Proteintech Group		
Anti-IκB-α	10268-1-AP	RRID:AB_2151423	WB (1:1000)	Rabbit	Proteintech Group		
Anti-NF-κBp50	sc-8414	RRID:AB_628015	WB/IF (1:300/1:350)	Mouse	Santa Cruz		
Anti-p-NF-κBp65	ab76302	RRID:AB_1524028	WB (1:1000)	Rabbit	Abcam		
Anti-IKKα	ab32041	RRID:AB_733070	WB (1:1000)	Rabbit	Abcam		
Anti-AP-1	24909-1-AP	RRID:AB_2860574	WB/IF (1:1000/1:350)	Rabbit	Proteintech Group		
Anti-p-AP-1	sc-822	RRID:AB_627262	WB (1:300)	Mouse	Santa Cruz		
Anti-Cx43	26980-1-AP	RRID:AB_2880711	WB/IF (1:1000/1:350)	Rabbit	Proteintech Group		
Anti-p-Cx43 (Ser368)	ab30559	RRID:AB_731707	WB (1:1000)	Rabbit	Abcam		
Anti-β-actin	20536-1-AP	RRID:AB_10700003	WB (1:2000)	Rabbit	Proteintech Group		
Anti-β-actin	66009-1-Ig	RRID:AB_2687938	WB (1:5000)	Mouse	Proteintech Group		
Anti-GAPDH	GB11002	RRID:AB_2904017	WB (1:2000)	Rabbit	Servicebio		
HRP-conjugated anti-Rabbit IgG	SA00001-2	RRID:AB_2722564	WB (1:5000)	Goat	Proteintech Group		
HRP-conjugated anti-Mouse IgG	SA00001-1	RRID:AB_2722565	WB (1:5000)	Goat	Proteintech Group		
FITC-conjugated anti-Rabbit IgG	SA00003-2	RRID:AB_2890897	IF (1:350)	Goat	Proteintech Group		
FITC-conjugated anti-Mouse IgG	SA00003-1	RRID:AB_2890896	IF (1:350)	Goat	Proteintech Group		
CY3-conjugated anti-Mouse IgG	SA00009-1	RRID:AB_2814746	IF (1:350)	Goat	Proteintech Group		

### Ethidium bromide uptake

For dye uptake experiments, astrocytes were stimulated with LPS for 24 h and exposed to 0.5 μM EtBr for 10 min at 37°C. EtBr is impermeable through membrane but can transit through hemichannels and becomes more fluorescent after binding to DNA. After 10 min exposure to EtBr, astrocytes were washed with PBS and stained with DAPI (Feng et al., 2018). Fluorescence photomicrographs were captured with a digital video camera (Olympus Corporation, Tokyo, Japan) connected to an inverted fluorescent microscope equipped with the appropriate filters (Olympus Corporation, Tokyo, Japan). We randomly chose 10 visual fields (nearly 50 cells per visual field) to count the percentage of the EtBr-positive cells by Image J software.

### Scrape-loading dye transfer assay

Gap junction permeability was determined by the scrape-loading/dye transfer technique as previously described by Gangoso et al. (2017). LY is a fluorescent dye that can pass through the gap junctions of loaded cells to their neighbors. Astrocytes were stimulated with LPS for 24 h. Then, scrape-loading was performed by scraping the cell layer with a broken razor blade in PBS containing LY (1 mg/ml). After 5 or 10 min, the dye solution was removed and the cells were carefully washed. Subsequently, fluorescence photomicrographs were captured and the diffusion distance of the dye was quantified using Image J software.

### ELISA assay

Cultured astrocytes were seeded in 12-well plates (1 × 10^6^ cells/well) pretreated with various antagonists followed by stimulation with LPS (1 μg/ml, 24 h). After stimulation, culture media were collected and centrifuged at 10,000 rpm for 5 min. The amounts of MCP-1 and CXCL1 in the culture medium were measured with commercial ELISA kits obtained from Shanghai Hushang Biological Technology Co., Ltd. China.

### Electrophoretic mobility shift assays

The supernatant was discarded from each group of cells, and the nuclear protein samples were obtained according to the manufacturer’s instructions of Minute™ Cytoplasmic and Nuclear Extraction Kit (cat. no. SC-003; Invent Biotechnologies, Inc, Dallas, TX, United States). Cells were washed twice with cold PBS and suspended in 120 μl of cytoplasmic extraction buffer (pH 7.9, 10 mM Hepes, 10 mM KCl, 0.1 mM EDTA, 0.1 mM EGTA, 1 mM dithiothreitol, 0.5 mM PMSF). The samples were vigorously vortexed for 15 s and lysed on ice for 2 min (repeat this for five times). Following lysis, the extract was centrifuged at 10,000 × *g* for 5 min to transfer the supernatant as the cytosol fraction. The nuclei were suspended in 60 μl of nuclear extraction buffer (20 mM Hepes, 20% glycerol, 0.4 M NaCl, 0.1 mM EDTA, 0.1 mM EGTA, 1 mM DTT, 0.2 mM PMSF). The samples were vigorously vortexed for 15 s and lysed on ice for 2 min (repeat this for five times). The extract was centrifuged at 10,000 × *g* for 5 min and the white microtransparent precipitation at the bottom was the nuclear protein. The nuclear protein was determined by using a BCA protein assay kit.

It is reported that the NF-κBp65 binding site in the promoter of MCP-1 gene (254 bp to 261 bp, p65-1: 5′-AGTTGAGGGGGACTTTCCCAGGC-3′) and the AP-1 binding site in the promoter of Cx43 gene (251 bp to 257 bp, AP-1-1: 5′-CGCTTGATGAGTCAGCCGGAA-3′) (Liu and Quinn, 2002; Teunissen et al., 2003). In addition, as shown in Table 2, the JASPAR and PROMO websites predicted the possible binding sequences of transcription factors (NF-κBp65 or AP-1) to the promoter region of MCP-1, CXCL1, or Cx43. We found that only one sets of high comparability DNA alignment with sequence logo for NF-κBp65 in the promoter region of MCP-1 and 2 binding sites in CXCL1, while with three binding sites for NF-κBp65 in the promoter region of Cx43. In addition, we found three sets of high comparability DNA alignment with sequence logo for AP-1 in the promoter region of MCP-1 and three binding sites in CXCL1, while with two binding sites for AP-1 in the promoter region of Cx43. According to the above information, to detected the interactions of transcription factors (NF-κBp65 or AP-1) and MCP-1 gene promoter, the following oligonucleotides were used: biotin 3’ end DNA-labeled NF-κBp65 probes (p65-1: 5′-AGTTGAGGGGGACTTTCCCAGGC-3′; p65-2: 5′-TAAAATGTGATTTCCCTTCAGT-3′) or AP-1 probes (AP-1-2: 5′-TATATCCCTGACACACCTGGGG-3′; AP-1-3: 5′-CCTCCTGCTGGGTCA GTTCTCCG-3′; AP-1-4: 5′-TGGGATGCTCAGTCATAGAGATG-3′). To detected the interactions of transcription factors (NF-κBp65 or AP-1) and CXCL1 gene promoter, the following oligonucleotides were used: biotin 3′ end DNA-labeled NF-κBp65 probes (p65-3: 5′-CTGTCCTGGAATGTCCTTGTCC-3′; p65-4: 5′-TTTACCTGGGATGTCCTCTCCT-3′) and AP-1 probes (AP-1-5: 5′-GGTCCTGACACAGAGTCACTGT-3′; AP-1-6: 5′-TTGGGATATGACTCTGGG GACA-3′; AP-1-7: 5′-AAATCTTTTGACTTATTCCATT-3′).

**TABLE 2:** JASPAR and PROMO analysis of the NF-κBp65, p50 or AP-1 binding sites in the promoter region of MCP-1, CXCL1, or Cx43.

Matrix ID	Name	Dissimilarity/Score	Sequence ID	Start	End	Predicted sequence
MA0107.1	p65-2	7.39%/8.94994	MCP-1NC_051345.1:6941206569413863	1663	1672	GTGATTTCCC
MA0107.1	p65-3	9.53128	CXCL1NC_051349.1:c1874545718743678	1349	1358	TGGAATGTCC
MA0107.1	p65-4	8.94994	CXCL1NC_051349.1:c1874545718743678	1612	1621	TGGGATGTCC
MA0107.1	p65-5	8.79582	Cx43NC_051355.1:3787665037889097	1575	1584	TGGATATTCC
MA0107.1	p65-6	8.67754	Cx43NC_051355.1:3787665037889097	239	248	GGGATTTTTC
MA0107.1	p65-7	8.0962	Cx43NC_051355.1:3787665037889097	2041	2050	GGTGTTTTCC
MA0105.1	p50-1	7.98083	MCP-1NC_051345.1:6941206569413863	389	398	GTGACATCCC
MA0105.1	p50-2	8.56885	MCP-1NC_051345.1:6941206569413863	1663	1672	GTGATTTCCC
MA0099.2	AP-1-2	0.81%/8.77298	MCP-1NC_051345.1:6941206569413863	201	207	TGACACA
MA0099.2	AP-1-3	0.61%/6.83623	MCP-1NC_051345.1:6941206569413863	460	466	TGGGTCA
MA0099.2	AP-1-4	4.74%/8.68023	MCP-1NC_051345.1:6941206569413863	883	889	TCAGTCA
MA0099.2	AP-1-5	2.55%/8.77298	CXCL1NC_051349.1:c1874545718743678	199	205	TGACACA
MA0099.2	AP-1-6	0.00%/7.61448	CXCL1NC_051349.1:c1874545718743678	323	329	TGACTCT
MA0099.2	AP-1-7	0.51%/6.74348	CXCL1NC_051349.1:c1874545718743678	667	673	TGACTTA
MA0099.2	AP-1-8	7.99993	Cx43NC_051355.1:3787665037889097	1236	1242	TGAGTCA
MA0099.2	AP-1-9	7.57255	Cx43NC_051355.1:3787665037889097	20	26	TTAATCA

At last, to detected the interactions of transcription factors (NF-κBp65 or AP-1) and Cx43 gene promoter, the following oligonucleotides were used: biotin 3′ end DNA-labeled NF-κBp65 probes (p65-5: 5′-TGTGTTTGGATATTCCGTGTTT-3′; p65-6: 5′-TCTTGGGGGATTTTTCCTTTGA-3′; p65-7: 5′-ACGTTTGGT GTTTTCCTCTGGC-3′) and AP-1 probes (AP-1-1: 5′-CGCTTGATGAGTCAG CCGGAA-3′; AP-1-8: 5′-TTGACAGTTGAGTCAATGATTTC-3′; AP-1-9: 5′-ATCAACATTTAATCATCTCCTCA-3′). In our study, these portions of the transcription factors/promoter region were PCR amplified and all labeled with biotin at 3′ end DNA.

It is reported that the p50–p65 heterodimers play a key role for MCP-1 gene expression in rat aortic endothelial cells (Panicker et al., 2010). Then, as shown in Table 2, the JASPAR and PROMO websites predicted the possible binding sequences of p50 to the promoter region of MCP-1. We found that two sets of high comparability DNA alignment with sequence logo for NF-κBp50 in the promoter region of MCP-1. In addition, the combination of p50 with the promoter regions of CXCL1 or Cx43 was no predicted with online websites JASPAR and PROMO. Furthermore, the combination of p100 with the promoter region of MCP-1, CXCL1 or Cx43 was also no detected with online websites JASPAR and PROMO. For this reason, to detect the interactions of p50 and MCP-1 gene promoter, the following oligonucleotides were used: biotin 3′ end DNA-labeled NF-κBp50 probes (p50-1: 5′-TGTGAGGTGACATCCCCAGATT-3′; p50-2: 5′-TAAAATGTGATTTCCCTTCAGT-3′).

The Thermo Scientific LightShift Chemiluminescent EMSA Kit (Thermo Scientific Pierce, Shanghai, China) was used to detect the interactions of transcription factors (NF-κBp65, p50 or AP-1) and gene promoter (MCP-1, CXCL1, or Cx43). Nuclear proteins were incubated with biotin-labeled oligonucleotide probes containing the binding sequence for MCP-1, CXCL1 or Cx43 (biotin 3′ end DNA labeling kit, Viagene Biotech Inc.). The binding reaction was performed by adding 8 μg of nuclear extracts, 20 fmol of biotin end-labeled DNA, and 1 μg/μl poly (dI-dC) to the end volume of 20 μl. For competition, a 100-fold molar excess of unlabeled cold NF-κBp65, p50 or AP-1 probe had the same sequence. After incubation for 20 min at room temperature, the reaction is then subjected to gel electrophoresis on a 5% native polyacrylamide gel and transferred to a nylon membrane. The biotin end-labeled DNA was detected using the streptavidin-horseradish peroxidase conjugate and the chemiluminescent substrate. The DNA binding activity was quantified by optical densitometry using Image J software. The binding activity was normalized to control and the results are shown as the percentage of control (% of control).

### Statistical analysis

All data were presented as mean ± standard deviation (SD). For this study, randomization and blinding were not performed. No statistical methods were used to determine the sample numbers in advance. No outlier test was conducted, thus, all data were included in the analysis. Hence, this should be considered an exploratory study.

Shapiro–Wilk tests were performed to examine normality of each data set. Since the distribution was even in most cases, parametric statistics were used to analyze the data. Data analysis was performed using the software Statistical Package for Social Sciences version 17.0 (SPSS Inc., Chicago, IL, United States). The One-Way ANOVA or Two-Way ANOVA plus a *post hoc* test were used for statistical analysis to compare the differences among treatment groups. Statistical Power analysis was performed by using the ‘General Linear model-Univariate’ function in SPSS 17.0 software. Where indicated, ‘n’ stands for the number of separate experiments carried out and, within each experiment, usually 4–5 technical replicates were performed for each condition/drug-treatment. For rat astrocyte cultures, a separate experiment is counted as cells extracted from different neonatal rat dorsal spinal cord. Differences at the *p* < 0.05 level were considered statistically significant. The statistical powers at the *p* > 0.8 level were considered as a real difference in different groups.

## Results

### TAK-242 inhibits lipopolysaccharide-induced astrocytic activation and MCP-1 and CXCL1 expression in cultured dorsal spinal cord astrocytes

**FIGURE 2:**
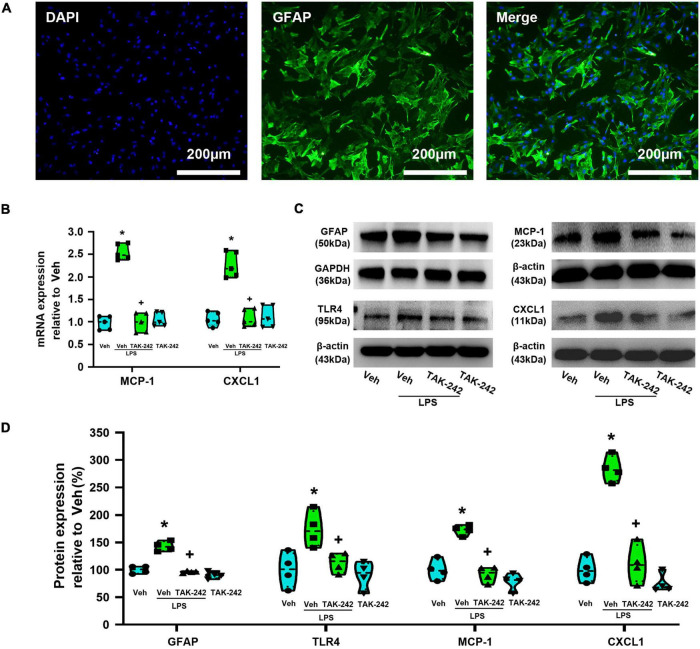
TAK-242 attenuates LPS-induced expression of GFAP, TLR4, MCP-1, and CXCL1 in cultured astrocytes. **(A)** Micrographs of purified astrocytes from rat dorsal spinal cord. High-magnification corresponding views of astrocytes labeled for DAPI (blue), GFAP (green), and their overlay (merge). Scale bar: 200 μm. **(B)** RT-qPCR quantitative analysis of MCP-1 and CXCL1 mRNA expression. *p < 0.05 vs. Veh-treated group, ^+^*p* < 0.05 vs. Veh + LPS-treated group, *n* = 5 per group. **(C)** WB images of GFAP, TLR4, MCP-1, and CXCL1 expression. The top panel was the target band, GFAP, TLR4, MCP-1, and CXCL1, and the bottom one was for the loading control GAPDH/β-actin. **(D)** Statistical analysis of the expression of GFAP, TLR4, MCP-1, and CXCL1 at the protein level [intensity ratios (normalized to β-actin or GAPDH expression) relative to the Veh-treated group]. *p < 0.05 vs. Veh-treated group, ^+^*p* < 0.05 vs. Veh + LPS-treated group, all data were expressed as mean ± standard deviation of at least four independent experiments (*n* = 4 per group).

In the present study, dorsal spinal cord astrocytes were identified with immunofluorescence stained with GFAP polyclonal rabbit antibody. The immunofluorescence staining after two generation showed that the purity of the astrocytes was more than 98% (Figure 2A). PCR and WB assays showed that the expression of MCP-1 and CXCL1 at the mRNA and protein levels were significantly increased in Veh + LPS group compared with Veh-treated group (*p* < 0.05, Power = 1, Figures 2B–D), whereas the effect was completely reversed by pretreatment with TAK-242 (100 nM for 2 h, *p* < 0.05, Power = 1). Pretreatment with TAK-242 significantly suppressed LPS-induced TLR4 and GFAP expression (TLR4: *p* < 0.05, Power = 0.935; GFAP*: p* < 0.05, Power = 1), which indicated that TLR4 activation is one of the key factors in LPS-induced astrocytic activation.

### TAK-242 significantly decreases lipopolysaccharide-induced NF-κB and AP-1 activation in cultured dorsal spinal cord astrocytes

**FIGURE 3:**
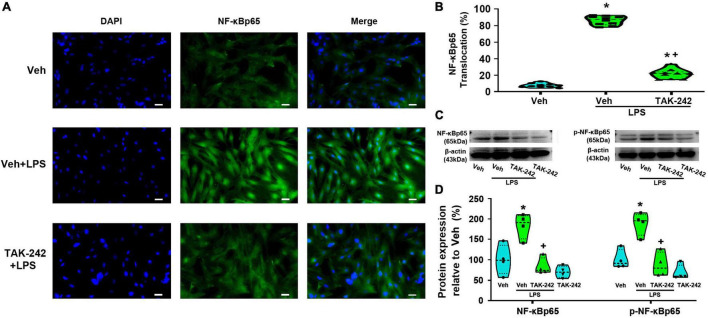
TAK-242 suppresses LPS-induced NF-κBp65 activity in cultured astrocytes. Cells were pretreated with TAK-242 (100 nM, 2 h) and then stimulated with LPS (1 μg/ml, 24 h). **(A)** Immunofluorescence staining images showed NF-κBp65 localization in astrocytes. Scale bar: 50 μm. **(B)** The percentage of nuclear p65-positive cells among the total cells was quantified. *p < 0.05 vs. Veh-treated group, ^+^*p* < 0.05 vs. Veh + LPS-treated group, *n* = 10 fields (at least 50 cells were counted per condition). **(C)** WB images of NF-κBp65 and p-NF-κBp65 expression. The top panel was the target band, NF-κBp65 and p-NF-κBp65, and the bottom one was for the loading control β-actin. **(D)** Statistical analysis of the protein expression level of NF-κBp65 and p-NF-κBp65 [intensity ratios (normalized to β-actin expression) relative to the Veh-treated group]. *p < 0.05 vs. Veh-treated group, ^+^*p* < 0.05 vs. Veh + LPS-treated group, all data were expressed as mean ± standard deviation of at least four independent experiments (*n* = 4 per group).

**FIGURE 4:**
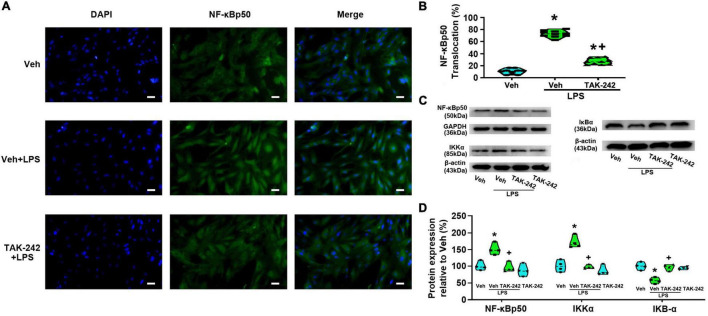
TAK-242 suppresses LPS-induced nuclear translocation of NF-κBp50 and the expression of NF-κBp50, IκB-α, and IKKα in cultured astrocytes. Cells were pretreated with TAK-242 (100 nM, 2 h) and then stimulated with LPS (1 μg/ml, 24 h). **(A)** Immunofluorescence staining images showed NF-κBp50 localization in astrocytes. Scale bar: 50 μm. **(B)** The percentage of nuclear p50-positive cells among the total cells was quantified. *p < 0.05 vs. Veh-treated group, ^+^*p* < 0.05 vs. Veh + LPS-treated group, *n* = 10 fields (at least 50 cells were counted per condition). **(C)** WB image of NF-κBp50, IκB-α, and IKKα protein expression. The top panel was the target band, NF-κBp50, IκB-α, and IKKα, and the bottom one was for the loading control β-actin/GAPDH. **(D)** Statistical analysis of the protein expression of NF-κBp50, IκB-α, and IKKα [intensity ratios (normalized to β-actin or GAPDH expression) relative to the Veh-treated group]. *p < 0.05 vs. Veh-treated group, ^+^*p* < 0.05 vs. Veh + LPS-treated group, all data were expressed as mean ± standard deviation of at least four independent experiments (*n* = 4 per group).

**FIGURE 5:**
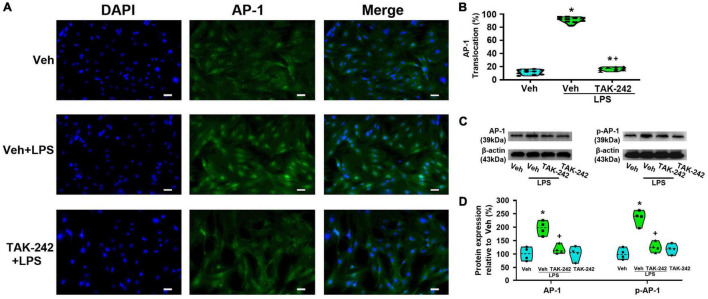
TAK-242 suppresses LPS-induced AP-1 activity in cultured astrocytes. Cells were pretreated with TAK-242 (100 nM, 2 h) and then stimulated with LPS (1 μg/ml, 24 h). **(A)** Immunofluorescence staining images showed AP-1 localization in astrocytes. Scale bar: 50 μm. **(B)** The percentage of nuclear AP-1-positive cells among the total cells was quantified. *p < 0.05 vs. Veh-treated group, ^+^*p* < 0.05 vs. Veh + LPS-treated group, *n* = 10 fields (at least 50 cells were counted per condition). **(C)** WB images of AP-1 and p-AP-1 protein expression. The top panel was the target band, AP-1 and p-AP-1, and the bottom one was for the loading control β-actin. **(D)** Statistical analysis of the protein expression of AP-1 and p-AP-1 [intensity ratios (normalized to β-actin expression) relative to the Veh-treated group]. *p < 0.05 vs. Veh-treated group, ^+^*p* < 0.05 vs. Veh + LPS-treated group, all data were expressed as mean ± standard deviation of at least four independent experiments (*n* = 4 per group).

We detected the protein level and activity of NF-κB and AP-1 in astrocytes before and after LPS stimulation. Immunofluorescence staining results showed that NF-κBp65 (Figures 3A,B), p50 (Figures 4A,B), and AP-1 (Figures 5A,B) were weakly expressed and mainly localized in the cytoplasm at quiescence state. After LPS stimulation, the expression of NF-κBp65, p50 and AP-1 were mainly localized in the nucleus (*p* < 0.05, Power = 1). The nuclear translocation rate of NF-κBp65, p50 and AP-1 in TAK-242 + LPS group was suppressed to 24.6, 25.2, and 19.9% of that in LPS-treated cells, respectively. It seems that TAK-242 pretreatment markedly reduced but not totally abolished the LPS-induced nuclear translocation of NF-κBp65, p50 and AP-1.

WB results showed that the expression of NF-κBp65 and p-NF-κBp65 were significantly increased in Veh + LPS group compared with Veh-treated cells, whereas the effect was completely reversed by TAK-242 (NF-κBp65: *p* < 0.05, Power = 0.998; p-NF-κBp65: *p* < 0.05, Power = 1, Figures 3C,D). Furthermore, LPS application increased the level of NF-κBp50 and IKKα protein and decreased the level of IκB-α in Veh + LPS group compared with Veh-treated group, whereas the effect was also completely reversed by TAK-242 (NF-κBp50: *p* < 0.05, Power = 0.998; IKKα: *p* < 0.05, Power = 1; IκB-α: *p* < 0.05, Power = 1, Figures 4C,D). In addition, WB results showed that the LPS-stimulated astrocytes exhibited a significant increase in AP-1 and p-AP-1 expression compared with Veh-treated group. Pretreatment with TAK-242 nearly completely inhibited the increased expression of AP-1 and p-AP-1 induced by LPS (AP-1: *p* < 0.05, Power = 0.999; p-AP-1: *p* < 0.05, Power = 1, Figures 5C,D). These results indicated that TLR4 activation is required for LPS-induced NF-κB and AP-1 activity, as well as their nuclear translocation.

### The treatment with PDTC or SR11302 significantly reduced the lipopolysaccharide-induced MCP-1 and CXCL1 expression in cultured dorsal spinal cord astrocytes

**FIGURE 6:**
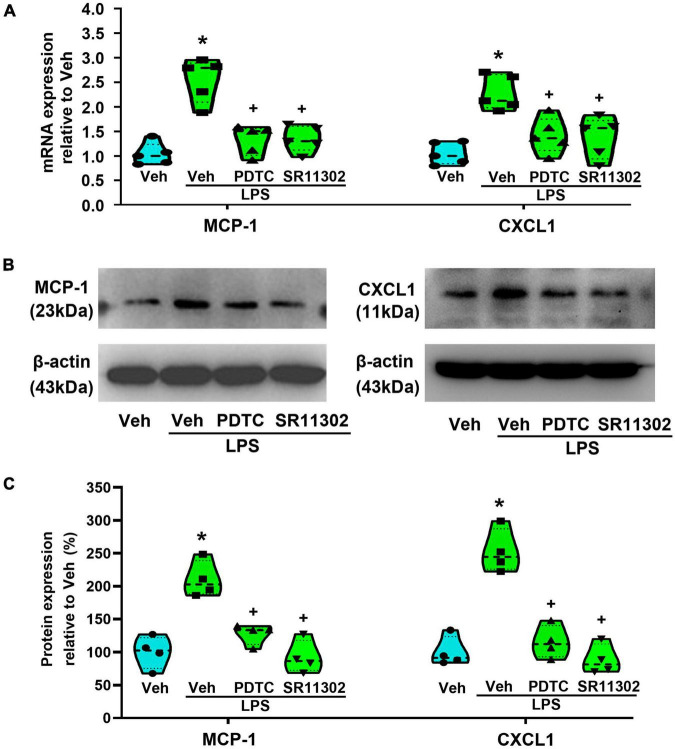
The treatment with PDTC or SR11302 inhibited LPS-induced MCP-1 and CXCL1 expression in cultured astrocytes. **(A)** RT-qPCR quantitative analysis of MCP-1 and CXCL1 mRNA expression. *p < 0.05 vs. Veh-treated group, ^+^*p* < 0.05 vs. Veh + LPS-treated group, *n* = 5 per group. **(B)** WB images of MCP-1 and CXCL1 expression. The top panel was the target band, MCP-1 and CXCL1, and the bottom one was for the loading control β-actin. **(C)** Statistical analysis of the protein expression of MCP-1 and CXCL1 [intensity ratios (normalized to β-actin expression) relative to the Veh-treated group]. *p < 0.05 vs. Veh-treated group, ^+^*p* < 0.05 vs. Veh + LPS-treated group, all data were expressed as mean ± standard deviation of at least four independent experiments (*n* = 4 per group).

To confirm the role of NF-κB or AP-1 in LPS-induced the expression of MCP-1 and CXCL1 in astrocytes, PDTC and SR11302 were used. As shown in Figure 6, both PCR and WB experiments demonstrated that, pretreatment with PDTC (1 μM, 30 min) or SR11302 (10 μM, 2 h) completely inhibited the expression of MCP-1 and CXCL1 at the mRNA (MCP-1: *p* < 0.05, Power = 1; CXCL1: *p* < 0.05, Power = 0.995) and protein levels (*p* < 0.05, Power = 1). It appears that LPS-induced increased expression of MCP-1 and CXCL1 is associated with the activation of the NF-κB or AP-1.

### The DNA-binding activity of NF-κB or AP-1 to the promoter regions of MCP-1 and CXCL1 in lipopolysaccharide-treated cultured dorsal spinal cord astrocytes

**FIGURE 7:**
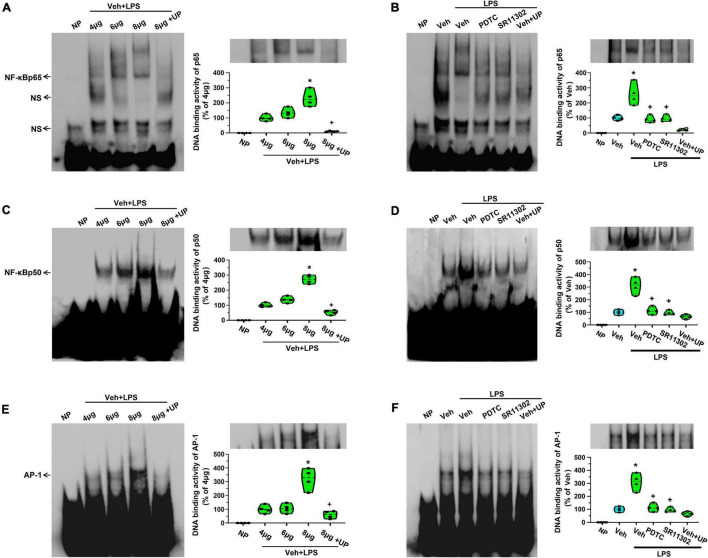
The treatment with PDTC or SR11302 decreased LPS-induced NF-κBp65, NF-κBp50, and AP-1 DNA binding activity in cultured astrocytes. **(A)** EMSA analysis was performed on binding reactions (the binding of NF-κBp65 to MCP-1 promoter) conducted in the presence of the indicated μg protein amount of Veh + LPS-treated cells (left). NP, no protein; UP, unlabeled probe; NS, non-specific band. NF-κBp65 DNA binding activity in Veh + LPS-treated cells was analyzed (right). *p < 0.05 vs. 4 μg group, ^ +^*p* < 0.05 vs. 8 μg group, *n* = 4 per group. **(B)** EMSA analysis was performed on binding reactions (the binding of NF-κBp65 to MCP-1 promoter) conducted in LPS-treated cells pretreated with or without PDTC or SR11302. For competition, a 100-fold molar excess of unlabelled probe (comp) was added to nuclear extracts from Veh + LPS group. This is a representative of three separate experiments (left). NF-κBp65 DNA binding activity in different groups was analyzed (right). *p < 0.05 vs. Veh-treated group, ^+^*p* < 0.05 vs. Veh + LPS-treated group, *n* = 4 per group. **(C)** EMSA analysis was performed on binding reactions (the binding of NF-κBp50 to MCP-1 promoter) conducted in the presence of the indicated μg protein amount of Veh + LPS-treated cells (left). NP, no protein; UP, unlabeled probe. NF-κBp50 DNA binding activity was analyzed. *p < 0.05 vs. 4 μg group, ^ +^*p* < 0.05 vs. 8 μg group, *n* = 4 per group. **(D)** EMSA analysis was performed on binding reactions (the binding of NF-κBp50 to MCP-1 promoter) conducted in LPS-treated cells pretreated with or without PDTC or SR11302. For competition, a 100-fold molar excess of unlabeled probe (comp) was added to nuclear extracts from Veh + LPS group. This is a representative of three separate experiments (left). NF-κBp50 DNA binding activity in different groups was analyzed (right). *p < 0.05 vs. Veh-treated group, ^+^*p* < 0.05 vs. Veh + LPS-treated group, *n* = 4 per group. **(E)** EMSA analysis was performed on binding reactions (the binding of AP-1 to CXCL1 promoter) conducted in the presence of the indicated μg protein amount of Veh + LPS-treated cells (left). NP, no protein; UP, unlabeled probe. AP-1 DNA binding activity was analyzed. *p < 0.05 vs. 4 μg group, ^ +^*p* < 0.05 vs. 8 μg group, *n* = 4 per group. **(F)** EMSA analysis was performed on binding reactions (the binding of AP-1 to CXCL1 promoter) conducted in LPS-treated cells pretreated with or without PDTC or SR11302. For competition, a 100-fold molar excess of unlabeled probe (comp) was added to nuclear extracts from Veh + LPS group. AP-1 DNA binding activity in different groups was analyzed (right). *p < 0.05 vs. Veh-treated group, ^+^*p* < 0.05 vs. Veh + LPS-treated group, *n* = 4 per group.

To further explore the DNA-binding activity of NF-κB or AP-1 to the promoter regions of MCP-1 and CXCL1 in astrocytes, EMSA was used to localize the transcription factors binding site (p65, p50, or AP-1) in the promoter regions of MCP-1 and CXCL1. As shown in Figures 7A,B, a single shift was observed in the presence of increasing amounts of purified nuclear extracts of cells activated for 24 h in the presence of LPS (p65-1: biotin 3’ end DNA-labeled probe containing a NF-κBp65-activated site element). Addition of excess cold unlabeled oligonucleotide completely abolished p65 bands confirming the specificity of the NF-κBp65 binding activity (*p* < 0.05, Power = 1). It appears that NF-κBp65 in astrocytes recognizes the sequence from bp 254 to 261 (5′-AGTTGAGGGGGACTTTCCCAGGC-3′) and may play a key role in the expression of MCP-1 in LPS-treated astrocytes. Although we tested several conditions, we could not observe a gel shift of the other probes (p65-2, AP-1-2, AP-1-3, and AP-1-4) with nuclear extracts from any groups (data not shown). The result does not support the idea that the binding of AP-1 to MCP-1 promoter is induced in LPS-treated astrocytes. In addition, as shown in Figures 7C,D, a single shift was observed in the presence of increasing amounts of purified nuclear extracts of cells activated for 24 h in the presence of LPS (p50-1: biotin 3’ end DNA-labeled probe containing a NF-κBp50-activated site element). Addition of excess cold unlabeled oligonucleotide completely abolished p50 bands confirming the specificity of the NF-κBp50 binding activity (*p* < 0.05, Power = 1). It appears that NF-κBp50 in astrocytes recognizes the sequence from bp 389 to 398 (5′-TGTGAGGTGACATCCCCAGATT-3′) and may be involved in LPS-induced MCP-1 gene expression.

Second, as shown in Figures 7E,F, a single shift was observed in the presence of increasing amounts of purified nuclear extracts of cells activated for 24 h in the presence of LPS (AP-1-6: biotin 3’ end DNA-labeled probe containing an AP-1-activated site element). Addition of excess cold unlabeled oligonucleotide completely abolished AP-1 bands confirming the specificity of the AP-1 binding activity (*p* < 0.05, Power = 1). It appears that AP-1 in astrocytes recognizes the sequence from bp 323 to 329 (5′-TTGGGATATGACTCTGGGGACA-3′) and may play a key role in the expression of CXCL1 in LPS-treated astrocytes. We could not observe a gel shift of the other probes (p65-3, p65-4, AP-1-5, and AP-1-7) with nuclear extracts from any groups (data not shown). This result seems does not support the idea that the binding of NF-κBp65 to CXCL1 promoter is induced in LPS-treated astrocytes.

As shown in Figures 7B,D,F, LPS application increased NF-κ Bp65-, NF-κ Bp50-, and AP-1-specific binding activities in cultured astrocytes. However, this activation was obviously diminished when cells were pretreated with PDTC or SR11302 (*p* < 0.05, Power = 1). It looks likely that LPS-induced MCP-1 and CXCL1 gene expression is related to the activation of NF-κB or AP-1.

**FIGURE 8:**
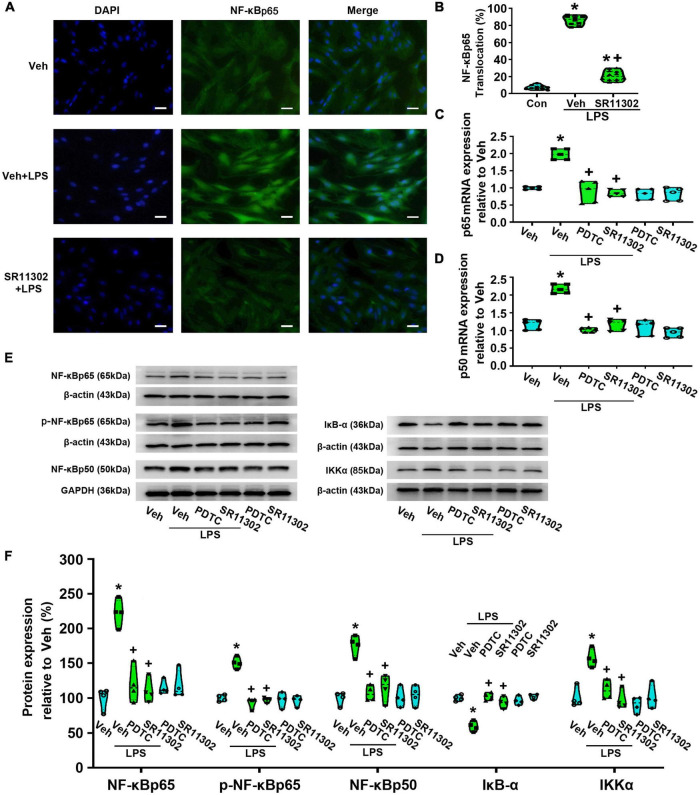
The treatment with PDTC or SR11302 decreased LPS-induced NF-κBp65 activity in cultured astrocytes. Cells were pretreated with PDTC (1 μM, 30 min) or SR11302 (10 μM, 2 h) and then stimulated with LPS (1 μg/ml, 24 h). **(A)** Immunofluorescence staining images showed NF-κBp65 localization. Scale bar: 50 μm. **(B)** The percentage of nuclear NF-κBp65-positive cells among the total cells was quantified. *p < 0.05 vs. Veh-treated group, ^+^*p* < 0.05 vs. Veh + LPS-treated group, *n* = 10 fields (at least 50 cells were counted per condition). **(C)** RT-qPCR quantitative analysis of NF-κBp65 mRNA expression. *p < 0.05 vs. Veh-treated group, ^+^*p* < 0.05 vs. Veh + LPS-treated group, *n* = 5 per group. **(D)** RT-qPCR quantitative analysis of NF-κBp50 mRNA expression. *p < 0.05 vs. Veh-treated group, ^+^*p* < 0.05 vs. Veh + LPS-treated group. **(E)** WB images of NF-κBp65, p-NF-κBp65, NF-κBp50, IκB-α, and IKKα protein expression. The top panel was the target band, NF-κBp65, p-NF-κBp65, NF-κBp50, IκB-α and IKKα, and the bottom one was for the loading control β-actin/GAPDH. **(F)** Statistical analysis of the protein expression of NF-κBp65, p-NF-κBp65, NF-κBp50, IκB-α, and IKKα [intensity ratios (normalized to β-actin or GAPDH expression) relative to the Veh-treated group]. *p < 0.05 vs. Veh-treated group, ^+^*p* < 0.05 vs. Veh + LPS-treated group, all data were expressed as mean ± standard deviation of at least four independent experiments (*n* = 4 per group).

Thus, to confirm whether SR11302 can suppress LPS-induced NF-κBp65 activation in astrocytes, we evaluated NF-κBp65 nuclear translocation rate. As shown in Figures 8A,B, immunofluorescence staining results showed that LPS-induced NF-κBp65 nuclear translocation was markedly reduced but not totally abolished by SR11302 (*p* < 0.05, Power = 1). Subsequently, compared with the Veh-treated astrocytes, the mRNA expression of NF-κBp65 and p50 in the LPS-treated astrocytes were significantly increased, while SR11302 significantly reversed this situation (*p* < 0.05, Power = 1, Figures 8C,D). The WB data confirmed this result. LPS-induced the increased expression of NF-κBp65, p-NF-κBp65, NF-κBp50, IKKα was significantly suppressed by SR11302 (*p* < 0.05, Power = 1, Figures 8E,F). In addition, pretreatment with SR11302 significantly inhibited LPS-induced IκB-α degradation (*p* < 0.05, Power = 1, Figures 8E,F).

**FIGURE 9:**
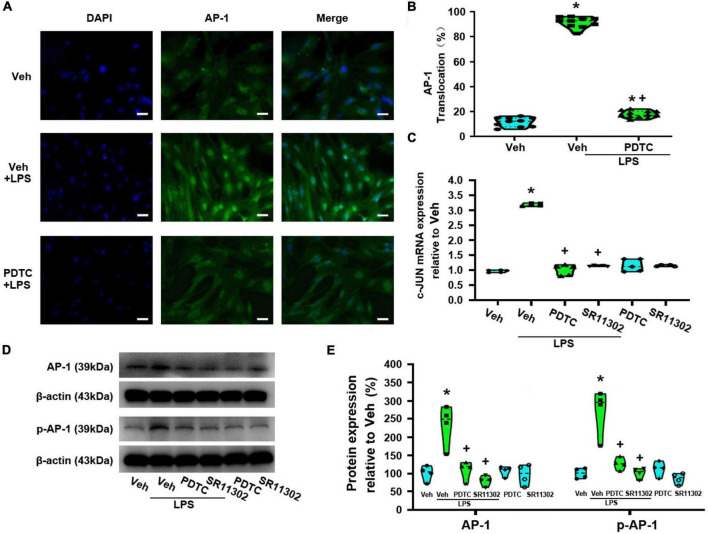
The treatment with of PDTC or SR11302 decreased LPS-induced AP-1 activity in cultured astrocytes. Cells were pretreated with PDTC (1 μM, 30 min) or SR11302 (10 μM, 2 h) and then stimulated with LPS (1 μg/ml, 24 h). **(A)** Immunofluorescence staining images showed AP-1 localization in astrocytes. Scale bar: 50 μm. **(B)** The percentage of nuclear AP-1-positive cells among the total cells was quantified. *p < 0.05 vs. Veh-treated group, ^+^*p* < 0.05 vs. Veh + LPS-treated group, *N* = 10 fields (at least 50 cells were counted per condition). **(C)** RT-qPCR quantitative analysis of c-Jun mRNA expression. *p < 0.05 vs. Veh-treated group, ^+^*p* < 0.05 vs. Veh + LPS-treated group, *n* = 5 per group. **(D)** WB images of AP-1 and p-AP-1 expression. The top panel was the target band, AP-1 and p-AP-1, and the bottom one was for the loading control β-actin. **(E)** Statistical analysis of the protein expression of AP-1 and p-AP-1 [intensity ratios (normalized to β-actin expression) relative to the Veh-treated group]. *p < 0.05 vs. Veh-treated group, ^+^*p* < 0.05 vs. Veh + LPS-treated group, all data were expressed as mean ± standard deviation of at least four independent experiments (*n* = 4 per group).

To confirm the role of NF-κBp65 activation in LPS-induced AP-1 activity, we evaluated AP-1 nuclear translocation rate. Compared with the Veh + LPS-treated group, PDTC treatment nearly completely abolished LPS-induced AP-1 nuclear translocation (*p* < 0.05, Power = 1, Figures 9A,B). Meanwhile, compared with the Veh-treated astrocytes, LPS increased the expression of AP-1 (c-Jun) at the mRNA level (*p* < 0.05, Power = 1), while PDTC significantly reversed this situation (*p* < 0.05, Power = 1, Figure 9C). Moreover, LPS-induced the increased expression of AP-1 and p-AP-1 was significantly suppressed by PDTC (AP-1: *p* < 0.05, Power = 0.852; p-AP-1: *p* < 0.05, Power = 1, Figures 9D,E).

### The treatment with TAK-242, ammonium pyrrolidinedithiocarbamate or SR11302 significantly reduced lipopolysaccharide-induced Cx43 expression in cultured dorsal spinal cord astrocytes

**FIGURE 10:**
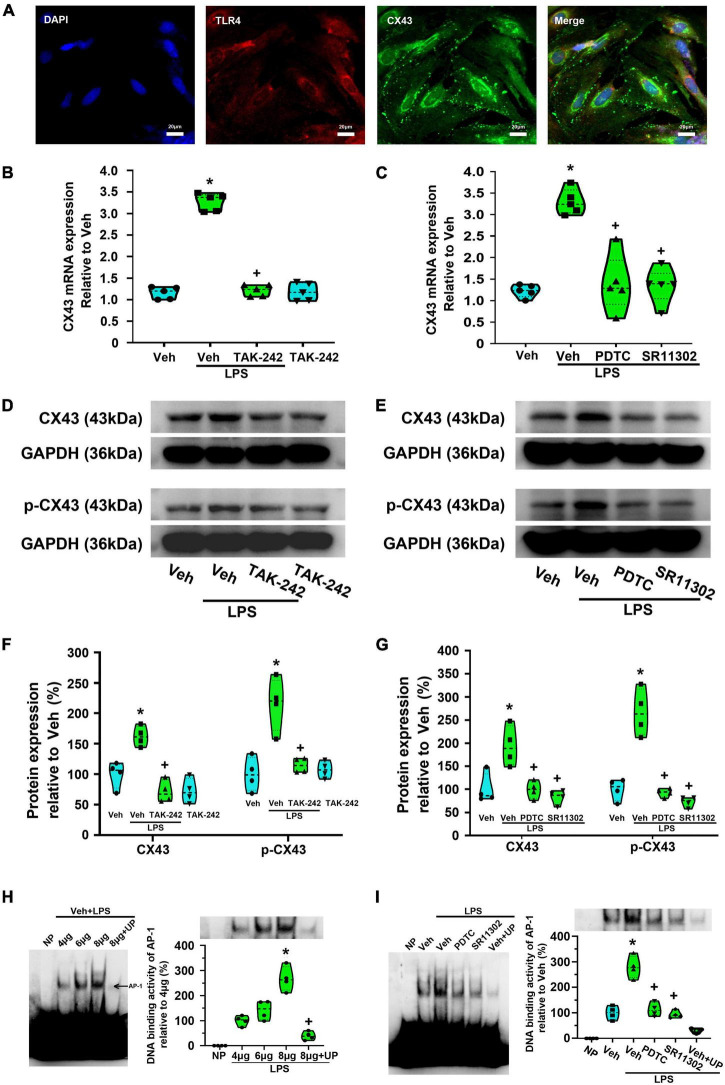
Effect of TAK-242, PDTC or SR11302 on LPS-induced Cx43 expression in cultured astrocytes. **(A)** Double immunofluorescence indicates the expression of TLR4 on Cx43-labeled dorsal spinal cord astrocytes. Scale bar: 20 μm. **(B)** RT-qPCR quantitative analysis showed that TAK-242 significantly inhibited LPS-induced Cx43 mRNA expression. *p < 0.05 vs. Veh-treated group, ^+^*p* < 0.05 vs. Veh + LPS-treated group, *n* = 5 per group. **(C)** RT-qPCR quantitative analysis showed that PDTC or SR11302 significantly inhibited LPS-induced Cx43 mRNA expression. *p < 0.05 vs. Veh-treated group, ^+^*p* < 0.05 vs. Veh + LPS-treated group, *n* = 5 per group. **(D)** WB results showed that TAK-242 significantly inhibited LPS-induced Cx43 and p-Cx43 expression. The top panel was the target band, Cx43 and p-Cx43 (Ser368), and the bottom one was for the loading control GAPDH. **(E)** WB results showed that PDTC or SR11302 significantly inhibited LPS-induced Cx43 and p-Cx43 expression. The top panel was the target band, Cx43 and p-Cx43 (Ser368), and the bottom one was for the loading control GAPDH. **(F)** Statistical analysis shows the effect of TAK-242 on Cx43 and p-Cx43 protein expression [intensity ratios (normalized to GAPDH expression) relative to the Veh-treated group]. *p < 0.05 vs. Veh-treated group, ^+^*p* < 0.05 vs. Veh + LPS-treated group, *n* = 4 per group**. (G)** Statistical analysis shows the effect of PDTC or SR11302 on Cx43 and p-Cx43 protein expression [intensity ratios (normalized to GAPDH expression) relative to the Veh-treated group]. *p < 0.05 vs. Veh-treated group, ^+^*p* < 0.05 vs. Veh + LPS-treated group, all data were expressed as mean ± standard deviation of at least four independent experiments (*n* = 4 per group). **(H)** EMSA analysis was performed on binding reactions (the binding of AP-1 to Cx43 promoter) conducted in the presence of the indicated μg protein amount of Veh + LPS-treated cells (left). NP, no protein; UP, unlabeled probe. AP-1 DNA binding activity was analyzed (right). *p < 0.05 vs. 4 μg group, ^ +^*p* < 0.05 vs. 8 μg group, *n* = 4 per group. **(I)** EMSA analysis was performed on binding reactions (the binding of AP-1 to Cx43 promoter) conducted in LPS-treated cells pretreated with or without PDTC or SR11302. This is a representative of three separate experiments (left). For competition, a 100-fold molar excess of unlabelled probe (comp) was added to nuclear extracts from Veh + LPS group. AP-1 DNA binding activity in different groups was analyzed (right). *p < 0.05 vs. Veh-treated group, ^+^*p* < 0.05 vs. Veh + LPS-treated group, *n* = 4 per group.

In the present study, immunofluorescence staining results indicated the expression of TLR4 on Cx43-labeled dorsal spinal cord astrocytes (Figure 10A). Compared with the Veh-treated astrocytes, LPS increased Cx43 expression at the mRNA and protein level, while the effect were significantly reversed by TAK-242 (*p* < 0.05, Power = 1, Figures 10B,D). Previously studies suggested that Cx43 phosphorylation at Ser368 is related to the dysregulation of cell-to-cell communication (Zhong et al., 2018). We found that TAK-242 treatment significantly inhibited the LPS-induced the increased p-Cx43 expression (*p* < 0.05, Power = 0.999, Figure 10D).

To confirm the role of TLR4-mediated NF-κBp65 and AP-1 activation in LPS-induced Cx43 expression, PDTC and SR11302 were used. As shown in Figures 10C,E, compared with the Veh + LPS-treated astrocytes, PDTC or SR11302 treatment significantly inhibited the LPS-induced Cx43 expression at mRNA (*p* < 0.05, Power = 1) and protein (*p* < 0.05, Power = 0.999) level, and its phosphorylation (*p* < 0.05, Power = 1).

Electrophoretic mobility shift assays were used to localize the transcription factors (NF-κBp65 or AP-1) binding site in Cx43 promoter. As shown in Figures 10H,I, a single shift was observed in the presence of increasing amounts of purified nuclear extracts of cells activated for 24 h in the presence of LPS (AP-1-1: biotin 3’ end DNA-labeled probe containing an AP-1-activated site element). Addition of excess cold unlabeled oligonucleotide completely abolished AP-1 bands confirming the specificity of the AP-1 binding activity (*p* < 0.05, Power = 1). It appears that AP-1 recognizes the sequence from bp 251 to 257 (5′-CGCTTGATGAGTCAGCCGGAA-3′) and may play a key role in Cx43 transcription in LPS-treated astrocytes. We could not observe a gel shift of the other probes (p65-5, p65-6, p65-7, AP-1-8, and AP-1-9) with nuclear extracts from any groups. This result seems does not support the idea that the binding of NF-κBp65 to Cx43 promoter is induced in LPS-treated astrocytes. Both PDTC and SR11302 significantly inhibited LPS-induced AP-1-specific binding activities in Cx43 promoter (*p* < 0.05, Power = 1). The NF-κB activation may indirectly affect LPS-induced AP-1-specific binding activities in LPS-induced Cx43 transcription.

### The treatment with TAK-242, CBX, Gap26, or Gap19 significantly reduced lipopolysaccharide-induced the release of MCP-1 and CXCL1 from cultured dorsal spinal cord astrocytes

**FIGURE 11:**
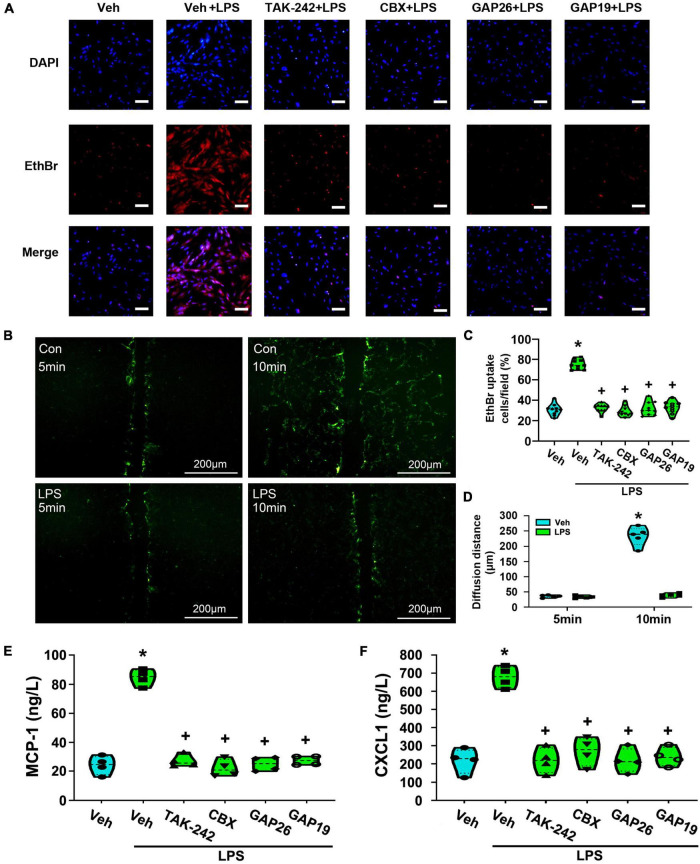
The treatment with TAK-242, CBX, Gap26 or Gap19 inhibited astrocyte hemichannel activity and the release of MCP-1 and CXCL1 from cultured astrocytes. **(A)** Representative photomicrographs showed EtBr uptake in astrocytes after LPS treatment and the inhibition promoted by TAK-242, CBX, Gap26, or Gap19. Scale bar: 100 μm. **(B)** Astrocytes were incubated in the presence of LY before and after LPS application. The fluorescence diffusion distance of LY is representative as the gap junction function in astrocytes. Scale bar: 200 μm. **(C)** Quantification of EtBr uptake intensity in different groups (at least 50 cells were counted per condition). *p < 0.05 vs. Veh-treated group, ^+^*p* < 0.05 vs. Veh + LPS-treated group. **(D)** The histogram showed an analysis of the distance between the dye transfer front and the scrape line of each group. *p < 0.05 vs. Veh-treated group, ^+^*p* < 0.05 vs. Veh + LPS-treated group, *n* = 5 per group. **(E)** The effect of LPS-induced MCP-1 release was suppressed by TAK-242, CBX, Gap26, or Gap19. *p < 0.05 vs. Veh-treated group, ^+^*p* < 0.05 vs. Veh + LPS-treated group, *n* = 4 per group. **(F)** The effect of LPS-induced CXCL1 release was suppressed by TAK-242, CBX, Gap26 or Gap19. *p < 0.05 vs. Veh-treated group, ^+^*p* < 0.05 vs. Veh + LPS-treated group, all data were expressed as mean ± standard deviation of at least four independent experiments (*n* = 4 per group).

EtBr uptake assay was used to measure the opening of Cx43 hemichannels in astrocytes. As shown in Figures 11A,C, LPS-treated astrocytes exhibited significantly greater EtBr uptake than that of the Veh-treated group (*p* < 0.05, Power = 1). However, pretreatment with TAK-242 (100 nM, 2 h), CBX (100 μM, 1 h), Gap26 (100 μM, 1 h) or Gap19 (100 μM, 1 h) all completely suppressed EtBr uptake than that of Veh + LPS-treated group (*p* < 0.05, Power = 1). It means that LPS application enhanced hemichannel activity in cultured astrocytes, whereas the effect was nearly totally reversed by TAK-242, CBX, Gap26, or Gap19.

The effect of LPS on the communication through gap junctions was explored by using a simple scrape loading/dye transfer assay. The diffusion distance of LY via gap junction migrates to neighboring cells and is considered as a marker to evaluate the activity of gap junction. As shown in Figures 11B,D, the diffusion distance of LY was longer in Veh-treated astrocytes. However, compared with the Veh-treated cells, the diffusion distance in Veh + LPS-treated cells was shortened upon LPS exposure (*p* < 0.05, Power = 1). It seems that LPS application significantly inhibited gap junction intercellular communication between astrocytes.

At last, the release of MCP-1 and CXCL1 from astrocytes was explores by using ELISA assay. As shown in Figures 11E,F, compared with Veh-treated astrocytes, LPS-induced the release of MCP-1 and CXCL1 was significantly increased (*p* < 0.05, Power = 1). Furthermore, LPS-induced the release of MCP-1 and CXCL1 were almost totally abolished in cultures co-incubated with TAK-242 (*p* < 0.05, Power = 1), CBX (*p* < 0.05, Power = 1), Gap26 (*p* < 0.05, Power = 1) or Gap19 (*p* < 0.05, Power = 1). It looks likely that TLR4-mediated Cx43 hemichannel opening is associated with LPS-induced the release of MCP-1 and CXCL1 from cultured astrocytes.

## Discussion

Lipopolysaccharide is a bacterial endotoxin that induces TLR4 activation to trigger inflammatory response. In the present study, LPS alone increases the expression of GFAP, TLR4, MCP-1, and CXCL1 in cultured rat dorsal spinal cord astrocytes, and the effect can be blocked by the TLR4-specific antagonist TAK-242. It means that TLR4 activation results in augmented astrogliosis reaction and the chemokine production.

In many different cell types, the transcriptional factors NF-κB and AP-1 play a pivotal role in TLR4-induced inflammatory response (Bian et al., 2013; Sun et al., 2017; Akhter et al., 2018; Wang et al., 2020). We evaluated the expression and activity of NF-κB and AP-1 in LPS-treated astrocytes. According to western blot analysis, LPS-induced the expression of NF-κBp65, p-NF-κBp65, IKKα, AP-1, and p-AP-1 was significantly reversed by TAK-242. At the same time, the expression of IκB-α in LPS-treated astrocyte was substantially reduced and recovered by TAK-242. The result clearly demonstrates that the expression and activity of NF-κB and AP-1 is regulated by TLR4 in LPS-treated astrocytes. The data from double-label immunofluorescence assays further confirmed that TLR4 activation is necessary for LPS-induced NF-κBp65 and AP-1 activation in astrocytes.

Recent studies have suggested that NF-κBp65 and AP-1 are important transcription factors for the expression of some inflammation-related genes involved in inflammatory response. For example, the chemokine gene transcripts MCP-1 and CXCL1 were induced via NF-κB activation by TNF-α in mouse cortical neurons (Listwak et al., 2013). The activation of NF-κB and AP-1 can induce MCP-1 expression in glioblastoma cells (Jung et al., 2018). Similarly, in agreement with these previous studies, we also found that LPS-stimulated astrocytes exhibited increased MCP-1 and CXCL1 expression, which was accompanied by the increased activity of NF-κBp65 and AP-1. More importantly, NF-κB inhibitor PDTC and AP-1 inhibitor SR11302 substantially suppressed LPS-mediated NF-κBp65 and AP-1 activity as well as the expression of MCP-1 and CXCL1 in astrocytes. It appears that TLR4-mediated NF-κBp65 and AP-1 signaling is required for LPS-induced MCP-1 and CXCL1 expression in cultured dorsal spinal cord astrocytes.

However, these above results did not allow a differentiation between direct and indirect effects of NF-κBp65 or AP-1 on target gene expression. Then, to get further insight into the molecular mechanisms that control these two genes expression at the chromatin level, we studied the promoter of MCP-1 and CXCL1. The presence of NF-κBp65 bands as seen in EMSA reflects a strong LPS induction of MCP-1 gene via NF-κBp65. In addition, the presence of NF-κBp50 bands as seen in EMSA reflects a strong LPS induction of MCP-1 gene via NF-κBp50. It appears that p65- and p50 subunit-dependent mechanism may be involved in LPS-induced MCP-1 gene expression. Despite Panicker et al. (2010) indicated that p50-p65 heterodimers is involved in MCP-1 gene expression in rat aortic endothelial cells, it is not clear whether p50–p65 heterodimers play a role in LPS-induced MCP-1 gene expression in dorsal spinal cord astrocytes. This needs to be confirmed with additional studies in the future.

More importantly, we found that the inhibition of AP-1 activity with SR11302 inhibited LPS-induced the expression of NF-κBp65 (mRNA, protein), NF-κBp50 (mRNA, protein), p-NF-κBp65 (protein), IKKα (protein). LPS-induced an increase in NF-κBp65 DNA-binding activity in MCP-1 promoter was also inhibited by SR11302. It looks likely that LPS-induced NF-κB activity is associated with AP-1 activation. The unexpected cross-coupling of two transcription factors may be involved in the MCP-1 transcription. Previous studies have suggested the importance of AP-1 in NF-κB activity. In the early years, Stein et al. (1993) reported that the bZIP regions of c-Fos and c-Jun are capable of physically interacting with NF-κBp65 through the Rel homology domain. Lately, Wang and Sonenshein (2005) suggested the role of AP-1 in transcriptional regulation of a gene encoding an NF-κB subunit, and its involvement in induction of RelB activity. In addition, Jun activation domain-binding protein 1 (Jab1) is expressed in brain astrocyte and is involved in AP-1 activation and I-κB ubiquitination (Luo et al., 2006; Orel et al., 2010). Despite these findings, it seems that the molecular mechanism governing this process awaits further investigation.

The activated c-Jun translocates into the nucleus, and interacts with Fos and other Jun proteins to generate AP-1 complex, which was followed by increased activation of AP-1. In many different cell types, the induction of AP-1 activity by LPS is related to NF-κBp65 activation (Krappmann et al., 2004; Yang et al., 2005; Dong et al., 2012; Kobelt et al., 2020). Moreover, it is reported that NF-κBp65 overexpression increased c-Jun mRNA expression and c-Jun-dependent promoter activity (Yang et al., 2005; Kobelt et al., 2020). These previously observations are consistent with our experimental results from PCR, western blot analysis and immunofluorescence staining for nuclear translocation of AP-1. It appears that NF-κBp65 play a key role in regulating both AP-1 (c-Jun) expression and its activity in LPS-treated astrocytes. More importantly, in the present study, the presence of AP-1 bands as seen in EMSA reflects a strong LPS induction of CXCL1 gene via AP-1. LPS-induced an increase in AP-1 DNA-binding activity in CXCL1 promoter was inhibited by PDTC or SR11302. While AP-1 may exert a direct effect on the CXCL1 promoter, it induces the expression of CXCL1 and NF-κB may play an indirectly role in CXCL1 transcription. More studies should be performed to detect the detailed mechanisms of the cross-talk between NF-κB and AP-1 families in astrocytic activation and inflammatory response.

In central nervous system, Cx43 forms two types of channels in astrocytes: gap junction channels for direct intercellular communication between astrocytes and unopposed hemichannels that permit cytosolic exchanges with the extracellular space (Chen et al., 2014). Our results showed that TLR4 signaling mediated the activation of NF-κB and AP-1 and thereby led to the Cx43 expression in LPS-treated astrocytes. The presence of AP-1 bands as seen in EMSA reflects a strong LPS induction of Cx43 gene via AP-1. This result is in agreement with the previous observation that Teunissen et al. (2003) reported. Unfortunately, we could not detect NF-κBp65 binding to Cx43 gene promoter region in gel shift assay. However, both PDTC and SR11302 were found to inhibit TLR4-mediated AP-1 DNA binding activity in Cx43 promoter. It appears that both the direct (AP-1) and indirect (NF-κBp65) mechanisms may be involved in TLR4-mediated Cx43 expression in LPS-treated astrocytes.

It is reported that Cx43 phosphorylation at Ser368 induces closure of gap junctional channels and opening of hemichannels in astrocytes (Yin et al., 2018; Shu et al., 2019). We found that the LPS-induced p-Cx43 (Ser368) expression was suppressed by TAK-242, PDTC, or SR11302. It seems that TLR4-mediated NF-κBp65 or AP-1 activation is associated with the closure of gap junctional channels and the opening of hemichannels. LPS-induced the release of MCP-1 and CXCL1 from astrocytes was inhibited by CBX, Gap26 or Gap19, which largely confirmed the role of Cx43 hemichannels in chemokines release from astrocytes. This observation is consistent with our experimental results from dye coupling assays for evaluating the activity of gap junctional channels and hemichannels.

In summary, the cell culture experiments reveal that the TLR4 is expressed in cultured dorsal spinal cord astrocytes, and participates in the LPS-evoked MCP-1 and CXCL1 expression. TLR4-mediated NF-κB and AP-1 activation is required for LPS-induced the expression of MCP-1, CXCL1 and Cx43. The binding of transcription factor NF-κBp65 to the MCP-1 promoter is a critical regulatory step in MCP-1 gene expression. The binding of transcription factor AP-1 to the promoter regions of CXCL1 and Cx43 is necessary for LPS-induced CXCL1 and Cx43 expression. In cultured astrocytes, the detailed mechanisms of the cross-talk between NF-κB and AP-1 families remains to be further explored. At last, we found that TLR4 activation increased Cx43 hemichannel, but not gap-junction activities and induced the release of MCP-1 and CXCL1 from astrocytes. This pathway may be important for physiological as well as pathophysiological events occurring within the spinal cord, where it is implicated in the transduction of the ‘pain’ message.

## Data availability statement

The original contributions presented in this study are included in the article/Supplementary Material, further inquiries can be directed to the corresponding author.

## Ethics statement

The animal study was reviewed and approved by Zunyi Medical University Committee on Ethics in the Care and Use of Laboratory Animals.

## Author contributions

YL and BL drafted the manuscript. YL, YX, and HJ performed the immunofluorescence and analysis. BL, AX, and XG performed the RT-qPCR and analysis. YL, TX, and RW performed the western blot and analysis. XL, YH, and JZ conceived of the study, participated in its design and coordination, and helped to draft the manuscript. XL performed the statistical analysis and drew violin plots. All authors read and approved the final manuscript.

## Conflict of interest

The authors declare that the research was conducted in the absence of any commercial or financial relationships that could be construed as a potential conflict of interest.

## Publisher’s note

All claims expressed in this article are solely those of the authors and do not necessarily represent those of their affiliated organizations, or those of the publisher, the editors and the reviewers. Any product that may be evaluated in this article, or claim that may be made by its manufacturer, is not guaranteed or endorsed by the publisher.

## Abbreviations

TLR4: toll-like receptor 4
Cx43: connexin-43
LPS: lipopolysaccharide
CBX: carbenoxolone
DMSO: dimethyl sulfoxide
DAPI: 4’,6-diamidino-2-phenylindole
RIPA: radioimmunoprecipitation
GAPDH: glyceraldehyde 3-phosphate dehydrogenase
EMSA: electrophoretic mobility shift assay
NF-κB: nuclear factor-κB
AP-1: activator protein 1
TNF-α: tumor necrosis factor-α
MCP-1: macrophage chemoattractant protein-1
CXCL1: [Chemokine (C-X-C motif) ligand 1]
RT-qPCR: real time quantitative polymerase chain reaction
PVDF: polyvinylidene fluoride
TBST: Tween-Tris-buffered saline
EtBr: ethidium bromide
LY: Lucifer Yellow
GFAP: glial fibrillary acidic protein
PDTC: ammonium pyrrolidinedithiocarbamate
Jab1: Jun activation domain-binding protein 1.

## References

[B1] Abg Abd Wahab D. Y. Gau C. H. Zakaria R. Muthu Karuppan M. K. A-Rahbi B. S. Abdullah Z. (2019). Review on cross talk between neurotransmitters and neuroinflammation in striatum and cerebellum in the mediation of motor behaviour. *Biomed Res. Int.* 2019:1767203. 10.1155/2019/1767203 31815123 PMC6877979

[B2] Abudara V. Roux L. Dallérac G. Matias I. Dulong J. Mothet J. P. (2015). Activated microglia impairs neuroglial interaction by opening Cx43 hemichannels in hippocampal astrocytes. *Glia* 63 795–811. 25643695 10.1002/glia.22785

[B3] Akhter N. Hasan A. Shenouda S. Wilson A. Kochumon S. Ali S. (2018). TLR4/MyD88 -mediated CCL2 production by lipopolysaccharide (endotoxin): implications for metabolic inflammation. *J. Diabetes Metab. Disord*. 17 77–84. 10.1007/s40200-018-0341-y 30288388 PMC6154519

[B4] Angeli S. Kousiappa I. Stavrou M. Sargiannidou I. Georgiou E. Papacostas S. S. (2020). Altered expression of glial gap junction proteins Cx43, Cx30, and Cx47 in the 5XFAD model of Alzheimer’s disease. *Front. Neurosci*. 14:582934. 10.3389/fnins.2020.582934 33117125 PMC7575794

[B5] Bennett M. V. Garré J. M. Orellana J. A. Bukauskas F. F. Nedergaard M. Sáez J. C. (2012). Connexin and pannexin hemichannels in inflammatory responses of glia and neurons. *Brain Res*. 1487 3–15. 10.1016/j.brainres.2012.08.042 22975435 PMC3627726

[B6] Bian Z. Peng Y. You Z. Wang Q. Miao Q. Liu Y. (2013). CCN1 expression in hepatocytes contributes to macrophage infiltration in nonalcoholic fatty liver disease in mice. *J. Lipid Res*. 54 44–54. 10.1194/jlr.M026013 23071295 PMC3520538

[B7] Burke S. J. Lu D. Sparer T. E. Masi T. Goff M. R. Karlstad M. D. (2014). NF-κB and STAT1 control CXCL1 and CXCL2 gene transcription. *Am. J. Physiol. Endocrinol. Metab*. 306 E131–E149. 10.1152/ajpendo.00347.2013 24280128 PMC3920007

[B8] Chen G. Park C. K. Xie R. G. Berta T. Nedergaard M. Ji R. R. (2014). Connexin-43 induces chemokine release from spinal cord astrocytes to maintain late-phase neuropathic pain in mice. *Brain* 137 2193–2209. 10.1093/brain/awu140 24919967 PMC4107738

[B9] Chen M. J. Kress B. Han X. Moll K. Peng W. Ji R. R. (2012). Astrocytic CX43 hemichannels and gap junctions play a crucial role in development of chronic neuropathic pain following spinal cord injury. *Glia* 60 1660–1670. 10.1002/glia.22384 22951907 PMC3604747

[B10] Deng X. Zhou X. Deng Y. Liu F. Feng X. Yin Q. (2017). Thrombin induces CCL2 expression in human lung fibroblasts via p300 mediated histone acetylation and NF-KappaB Activation. *J. Cell. Biochem.* 118 4012–4019. 10.1002/jcb.26057 28407300

[B11] Dong W. Li Y. Gao M. Hu M. Li X. Mai S. 2012. IKKα contributes to UVB-induced VEGF expression by regulating AP-1 transactivation. *Nucleic Acids Res.* 40 2940–2955. 10.1093/nar/gkr1216 22169952 PMC3326327

[B12] Du S. H. Qiao D. F. Chen C. X. Chen S. Liu C. Lin Z. (2017). Toll-Like Receptor 4 mediates methamphetamine-induced neuroinflammation through caspase-11 signaling pathway in astrocytes. *Front. Mol. Neurosci.* 10:409. 10.3389/fnmol.2017.00409 29311802 PMC5733023

[B13] Feng J. Li M. Wei Q. Li S. Song S. Hua Z. (2018). Unconjugated bilirubin induces pyroptosis in cultured rat cortical astrocytes. *J. Neuroinflammation* 15:23. 10.1186/s12974-018-1064-1 29357878 PMC5776766

[B14] Gangoso E. Talaverón R. Jaraíz-Rodríguez M. Domínguez-Prieto M. Ezan P. Koulakoff A. (2017). A c-Src inhibitor peptide based on connexin43 exerts neuroprotective effects through the inhibition of glial hemichannel activity. *Front. Mol. Neurosci*. 10:418. 10.3389/fnmol.2017.00418 29326548 PMC5737028

[B15] Gao F. H. Wang Q. Wu Y. L. Li X. Zhao K. W. Chen G. Q. (2007). c-Jun N-terminal kinase mediates AML1-ETO protein-induced connexin-43 expression. *Biochem. Biophys. Res. Commun*. 356 505–511. 10.1016/j.bbrc.2007.03.009 17367753

[B16] Gorina R. Font-Nieves M. Márquez-Kisinousky L. Santalucia T. Planas A. M. (2011). Astrocyte TLR4 activation induces a proinflammatory environment through the interplay between MyD88-dependent NFκB signaling, MAPK, and Jak1/Stat1 pathways. *Glia* 59 242–255. 10.1002/glia.21094 21125645

[B17] Guillebaud F. Barbot M. Barbouche R. Brézun J. M. Poirot K. Vasile F. (2020). Blockade of glial connexin 43 hemichannels reduces food intake. *Cells* 9:2387. 10.3390/cells9112387 33142723 PMC7693394

[B18] Guo H. Peng Z. Yang L. Liu X. Xie Y. Cai Y. (2017). TREK-1 mediates isoflurane-induced cytotoxicity in astrocytes. *BMC Anesthesiol.* 17:124. 10.1186/s12871-017-0420-5 28870160 PMC5584525

[B19] Imaizumi T. Murakami K. Ohta K. Seki H. Matsumiya T. Meng P. (2013). MDA5 and ISG56 mediate CXCL10 expression induced by toll-like receptor 4 activation in U373MG human astrocytoma cells. *Neurosci. Res*. 76 195–206. 10.1016/j.neures.2013.05.002 23684765

[B20] Jung Y. Ahn S. H. Park H. Park S. H. Choi K. Choi C. (2018). MCP-1 and MIP-3α secreted from necrotic cell-treated glioblastoma cells promote migration/infiltration of microglia. *Cell. Physiol. Biochem*. 48 1332–1346. 10.1159/000492092 30048972

[B21] Kobelt D. Zhang C. Clayton-Lucey I. A. Glauben R. Voss C. Siegmund B. (2020). Pro-inflammatory TNF-α and IFN-γ promote tumor growth and metastasis via induction of MACC1. *Front. Immunol.* 11:980. 10.3389/fimmu.2020.00980 32670264 PMC7326113

[B22] Krappmann D. Wegener E. Sunami Y. Esen M. Thiel A. Mordmuller B. (2004). The IkappaB kinase complex and NF-kappaB act as master regulators of lipopolysaccharide-induced gene expression and control subordinate activation of AP-1. *Mol. Cell. Biol.* 24 6488–6500. 10.1128/MCB.24.14.6488-6500.2004 15226448 PMC434242

[B23] Lennard Richard M. L. Nowling T. K. Brandon D. Watson D. K. Zhang X. K. (2015). Fli-1 controls transcription from the MCP-1 gene promoter, which may provide a novel mechanism for chemokine and cytokine activation. *Mol. Immunol*. 63 566–573. 10.1016/j.molimm.2014.07.013 25108845 PMC4254164

[B24] Liddell J. R. Lehtonen S. Duncan C. Keksa-Goldsteine V. Levonen A. L. Goldsteins G. (2016). Pyrrolidine dithiocarbamate activates the Nrf2 pathway in astrocytes. *J. Neuroinflammation* 13:49. 10.1186/s12974-016-0515-9 26920699 PMC4768425

[B25] Listwak S. J. Rathore P. Herkenham M. (2013). Minimal NF-κB activity in neurons. *Neuroscience* 250 282–299. 10.1016/j.neuroscience.2013.07.013 23872390 PMC3785079

[B26] Liu S. Lu C. Liu Y. Zhou X. Sun L. Gu Q. (2018). Hyperbaric oxygen alleviates the inflammatory response induced by LPS through inhibition of NF-κB/MAPKs-CCL2/CXCL1 signaling pathway in cultured astrocytes. *Inflammation* 41 2003–2011. 10.1007/s10753-018-0843-2 30073566

[B27] Liu Y. Quinn M. R. (2002). Chemokine production by rat alveolar macrophages is inhibited by taurine chloramine. *Immunol. Lett*. 80 27–32. 10.1016/s0165-2478(01)00291-7 11716962

[B28] Lu X. Ma L. Ruan L. Kong Y. Mou H. Zhang Z. (2010). Resveratrol differentially modulates inflammatory responses of microglia and astrocytes. *J. Neuroinflammation* 7:46. 10.1186/1742-2094-7-46 20712904 PMC2936301

[B29] Luo W. Wang Y. Hanck T. Stricker R. Reiser G. (2006). Jab1, a novel protease-activated receptor-2 (PAR-2)-interacting protein, is involved in PAR-2-induced activation of activator protein-1. *J. Biol. Chem.* 281 7927–7936. 10.1074/jbc.M510784200 16410250

[B30] Muhammad T. Ikram M. Ullah R. Rehman S. U. Kim M. O. (2019). Hesperetin, a citrus flavonoid, attenuates LPS-induced neuroinflammation, apoptosis and memory impairments by modulating TLR4/NF-κB signaling. *Nutrients* 11:648. 10.3390/nu11030648 30884890 PMC6471991

[B31] Oliveira-Junior M. S. Pereira E. P. de Amorim V. Reis L. do Nascimento R. P. da Silva V. (2019). Lupeol inhibits LPS-induced neuroinflammation in cerebellar cultures and induces neuroprotection associated to the modulation of astrocyte response and expression of neurotrophic and inflammatory factors. *Int. Immunopharmacol*. 70 302–312. 10.1016/j.intimp.2019.02.055 30852286

[B32] Orel L. Neumeier H. Hochrainer K. Binder B. R. Schmid J. A. (2010). Crosstalk between the NF-kappaB activating IKK-complex and the CSN signalosome. *J Cell. Mol. Med.* 14 1555–1568. 10.1111/j.1582-4934.2009.00866.x 19656241 PMC3829021

[B33] Panicker S. R. Sreenivas P. Babu M. S. Karunagaran D. Kartha C. C. (2010). Quercetin attenuates Monocyte Chemoattractant Protein-1 gene expression in glucose primed aortic endothelial cells through NF-kappaB and AP-1. *Pharmacol. Res*. 62 328–336. 10.1016/j.phrs.2010.06.003 20542118

[B34] Qian B. Li F. Zhao L. X. Dong Y. L. Gao Y. J. Zhang Z. J. (2016). Ligustilide ameliorates inflammatory pain and inhibits TLR4 upregulation in spinal astrocytes following complete freund’s adjuvant peripheral injection. *Cell. Mol. Neurobiol*. 36 143–149. 10.1007/s10571-015-0228-0 26115624 PMC11482452

[B35] Shu Y. Zhu C. Zeng M. Zhan Q. Hu Z. Wu X. (2019). The protective effect of carbenoxolone on gap junction damage in the hippocampal CA1 area of a temporal lobe epilepsy rat model. *Ann. Transl. Med.* 7:624. 10.21037/atm.2019.11.04 31930025 PMC6944627

[B36] Stein B. Baldwin A. S. Jr. Ballard D. W. Greene W. C. Angel P. Herrlich P. (1993). Cross-coupling of the NF-kappa B p65 and Fos/Jun transcription factors produces potentiated biological function. *EMBO J.* 12 3879–3891. 10.1002/j.1460-2075.1993.tb06066.x 8404856 PMC413671

[B37] Sun L. Li M. Ma X. Feng H. Song J. Lv C. (2017). Inhibition of HMGB1 reduces rat spinal cord astrocytic swelling and AQP4 expression after oxygen-glucose deprivation and reoxygenation via TLR4 and NF-κB signaling in an IL-6-dependent manner. *J. Neuroinflammation* 14:231. 10.1186/s12974-017-1008-1 29178911 PMC5702193

[B38] Sutcliffe A. M. Clarke D. L. Bradbury D. A. Corbett L. M. Patel J. A. Knox A. J. (2009). Transcriptional regulation of monocyte chemotactic protein-1 release by endothelin-1 in human airway smooth muscle cells involves NF-kappaB and AP-1. *Br. J. Pharmacol*. 157 436–450. 10.1111/j.1476-5381.2009.00143.x 19371341 PMC2707990

[B39] Takaki J. Fujimori K. Miura M. Suzuki T. Sekino Y. Sato K. (2012). L-glutamate released from activated microglia downregulates astrocytic L-glutamate transporter expression in neuroinflammation: the ‘collusion’ hypothesis for increased extracellular L-glutamate concentration in neuroinflammation. *J. Neuroinflammation* 9:275. 10.1186/1742-2094-9-275 23259598 PMC3575281

[B40] Teunissen B. E. Jansen A. T. van Amersfoorth S. C. O’Brien T. X. Jongsma H. J. Bierhuizen M. F. (2003). Analysis of the rat connexin 43 proximal promoter in neonatal cardiomyocytes. *Gene* 322 123–136. 10.1016/j.gene.2003.08.011 14644504

[B41] Wang L. Yang J. W. Lin L. T. Huang J. Wang X. R. Su X. T. (2020). Acupuncture attenuates inflammation in microglia of vascular dementia rats by inhibiting miR-93-mediated TLR4/MyD88/NF-κB signaling pathway. *Oxid. Med. Cell. Longev*. 2020:8253904. 10.1155/2020/8253904 32850002 PMC7441436

[B42] Wang X. Sonenshein G. E. (2005). Induction of the RelB NF-kappaB subunit by the cytomegalovirus IE1 protein is mediated via Jun kinase and c-Jun/Fra-2 AP-1 complexes. *J. Virol.* 79 95–105. 10.1128/JVI.79.1.95-105.2005 15596805 PMC538727

[B43] Wang Y. P. Wu Y. Li L. Y. Zheng J. Liu R. G. Zhou J. P. (2011). Aspirin-triggered lipoxin A4 attenuates LPS-induced pro-inflammatory responses by inhibiting activation of NF-κB and MAPKs in BV-2 microglial cells. *J. Neuroinflammation* 8:95. 10.1186/1742-2094-8-95 21831303 PMC3162900

[B44] Xian P. Hei Y. Wang R. Wang T. Yang J. Li J. (2019). Mesenchymal stem cell-derived exosomes as a nanotherapeutic agent for amelioration of inflammation-induced astrocyte alterations in mice. *Theranostics* 9 5956–5975. 10.7150/thno.33872 31534531 PMC6735367

[B45] Yang C. C. Hsiao L. D. Yang C. M. (2020). Galangin inhibits LPS-induced MMP-9 expression via suppressing protein kinase-dependent AP-1 and FoxO1 activation in rat brain astrocytes. *J. Inflamm. Res*. 13 945–960. 10.2147/JIR.S276925 33244253 PMC7685391

[B46] Yang H. Magilnick N. Ou X. Lu S. C. (2005). Tumour necrosis factor alpha induces co-ordinated activation of rat GSH synthetic enzymes via nuclear factor kappaB and activator protein-1. *Biochem. J*. 391 399–408. 10.1042/BJ20050795 16011481 PMC1276939

[B47] Ye Z. C. Oberheim N. Kettenmann H. Ransom B. R. (2009). Pharmacological “cross-inhibition” of connexin hemichannels and swelling activated anion channels. *Glia* 57 258–269. 10.1002/glia.20754 18837047 PMC2676787

[B48] Yin X. Feng L. Ma D. Yin P. Wang X. Hou S. (2018). Roles of astrocytic connexin-43, hemichannels, and gap junctions in oxygen-glucose deprivation/reperfusion injury induced neuroinflammation and the possible regulatory mechanisms of salvianolic acid B and carbenoxolone. *J. Neuroinflammation* 15:97. 10.1186/s12974-018-1127-3 29587860 PMC5872583

[B49] Zeng J. W. Liu X. H. Zhang J. H. Wu X. G. Ruan H. Z. (2008). P2Y1 receptor-mediated glutamate release from cultured dorsal spinal cord astrocytes. *J. Neurochem*. 106 2106–2118. 10.1111/j.1471-4159.2008.05560.x 18627435

[B50] Zhao L. X. Jiang B. C. Wu X. B. Cao D. L. Gao Y. J. (2014). Ligustilide attenuates inflammatory pain via inhibition of NFκB-mediated chemokines production in spinal astrocytes. *Eur. J. Neurosci*. 39 1391–1402. 10.1111/ejn.12502 24521480

[B51] Zhao W. Beers D. R. Bell S. Wang J. Wen S. Baloh R. H. (2015). TDP-43 activates microglia through NF-κB and NLRP3 inflammasome. *Exp. Neurol.* 273 24–35. 10.1016/j.expneurol.2015.07.019 26222336

[B52] Zhong C. Chang H. Wu Y. Zhou L. Wang Y. Wang M. (2018). Up-regulated Cx43 phosphorylation at Ser368 prolongs QRS duration in myocarditis. *J. Cell. Mol. Med*. 22 3537–3547. 10.1111/jcmm.13631 29664174 PMC6010859

