# Supplementary material for: NF-κB and AP-1 are required for the lipopolysaccharide-induced expression of MCP-1, CXCL1, and Cx43 in cultured rat dorsal spinal cord astrocytes
Source: Front Mol Neurosci. 2022 Jul 28;15:859558. doi: 10.3389/fnmol.2022.859558 (PMC9368326; doi:10.3389/fnmol.2022.859558)
Supplement: Supplementary file 1 [file Data_Sheet_1.PDF]

Rat mcp-1 Sequence 181-900

CCACCTGCCT TGTATATCCC TGACACACCT GGGGAAAGGG GCAGGGCCTG GGGTGGCAGT
5′-TATATCCCTGACACACCTGGGG-3′(AP-1-2)

TGGTACTTCA TAGGTTGAGG GTGGAGAGAA CTGAGATGCC CTCCACCTCC ATGGAATGCG
5′-AGTTGAGGGGGACTTTCCCAGGC-3′(NF-κBp65-1)

TCCGTTCCCC ATCTCTTTCC TCTTCACTAA AATATTTTGT TTAATCAGTG TTAACGAGGT

GGTTATCAGT AAGTGTGGTG GCTGTGAGGT GACATCCCCA GATTCAGTCT GGAGATAAAC
5′-TGTGAGGTGACATCCCCAGATT-3′(NF-κBp50-1)

TGGTTGGTGG AGGAGTCCAT GCTCCTCACC TCCTCCTGCT GGGTCAGTTC TCCGGGAGCT
5′-CCTCCTGCTGGGTCAGTTCTCCG-3′(AP-1-3)

CCGAGCTCCG TTTGATTTCT CACTTCTGGC CACCACTTGT TCAGCATCAG CTTCCTCCAG

TCTGACCTCC CCTTAGGGAG GGGAGGTAGG ATTGTGTGGT CAAAGAGATG GATCCCTTCC

CCCAGAGAGA TAAGTTGGAG ATGTGAGCAC AAGGGGCAGA ATTAACAGCT TCAGGCCTCT

GCCTGCTAGG CTAGCTTCCT GCTCCCAGGA TGGGAGGGCC AGCCCCCTGC CTCTCCTTTT

GAGCCAGGCA AAGGCAGGGC TCTCCAGTTT ACTTAAGAGT GGTCCCATCT GCTCAAACTG

GCCTTTCATA AGGGGAGGGA TTTATGCAGT GTCCTTGTGC TTAGACCTGG GATTTGGCTG

AAAACCTTGG GCCTCTGAGA ATATTACCTA ATGCTGGGAT GCTCAGTCAT AGAGATGATT
5′-TGGGATGCTCAGTCATAGAGATG-3′(AP-1-4)

Rat mcp-1 Sequence 1620-1680

CAACCTACAA GTGTCATTTA GTTATCTTCT CTAGGATAAA ATGTGATTTC CCTTCAGTGT

5′-TAAAATGTGATTTCCCTTCAGT-3′(NF-κBp65/50-2)

Rat cxcl1 Sequence 181-720

CATCTCAAGT GTTGGTCCTG ACACAGAGTC ACTGTTTAAC TGCCATATAC ATTAGGCTAC

5′-GGTCCTGACACAGAGTCACTGT-3′(AP-1-5)

CTGGCCCATG AGTTTCTGAA CATTTCTTTG CCCCTGACTC CAATCTGGCT ATAGGAGTGC

TGGGATTACA GATGTTGGGA TATGACTCTG GGGACAGGCA TGGGCTCTGG GGTTAGAACA

5′-TTGGGATATGACTCTGGGGACA-3′(AP-1-6)

TGAGTCTTCA TGTGTGTGTG GTTAGTATAA TGGCACTGAA CCATCATCCA GCTCCCAGCT

CTTTTCTTAC TCTAACTGAA AACAGTTCCA AGATGCACTT TCTCTGTGAG TTATCCTCGC

ACAGACCCTG TGGACCTGCT TTGTGGTAAA GCTCTTCTCC TCACTATTAA AGCCTTGGCC

CCCCTTCCAT TCCTACCCTC CCATGGGAAC TCATTGTCTC TCCCTTCCTG GGCTGTACTG

CTCAGCTCGA TTTTTTCCTG TACTGTGTCC CGATCTGTGA TCCACAATAC ACAGAGAGAA

ATCTTTTGAC TTATTCCATT TCTGTGCTTA ATATCCTCTC ATAGCTCCCT ATAGCTTTCA

5′-AAATCTTTTGACTTATTCCATT-3′(AP-1-7)

Rat cxcl1 Sequence 1331-1630

TCCCACAACT GCCTGTCCTG GAATGTCCTT GTCCTCTGGG CTTAGATTAG TGTACCCTAG
5′-CTGTCCTGGAATGTCCTTGTCC-3′(NF-κBp65-3)
GATTTGTCAC CCTAGTTTCT CTCTGCATAT TTTCTTGGCA AGCCATGAAA TTAACAGGAG

CTTCTTACTA CCATTAAATC TAATCAACCA AATTCACCGT TCACTGAAAT AACCCTTGGA

GATTTGACAT GGGCTAGCCA GTTGATGAGG ACAATGTGCT TCATGATCAG ATGGGCTGAA

GAATTAACTA AGTGCCTGTC ATGCCCTTTC TTCCCTTTAC CTGGGATGTC CTCTCCTTGT
5′-TTTACCTGGGATGTCCTCTCCT-3′(NF-κBp65-4)

Rat cx43 Sequence 1-300

AGGATGCTGA CATCAACATT TAATCATCTC CTCACCAATC CAGGAAGAAG GGGAGATCAG

5′-ATCAACATTTAATCATCTCCTCA-3′(AP-1-9)

TTACTACTGT ACTTTATTGT GTTCAACCAA ATCACCATGT TACAAAAATA GCAAGCTGCC

ATAATAAAAA ATAAGGCTCC TCTATCCAGC ACCAGATAGC ATCATTTTAC TTTCAAGGCT

AGAAATTGCA CACTTGTATA TAAACCAACC GAAGATGAGG ATTGAGAGTT CATCTTGGGG

5′-TCTTGGGGGATTTTTCCTTTGA-3′(NF-κBp65-6)

GATTTTTCCT TTGATGAATA TGAAGTGTCC TTCTTTATCT TTTTTGATGA CTTTTAATTG

5′-CGCTTGATGAGTCAGCCGGAA-3′(AP-1-1)

Rat cx43 Sequence 1226-2065

CTTTGACAGT TGAGTCAATG ATTTCTATGG AATCTTCTAC TCCCGAGATT CTCTCTTCCA
5′-TTGACAGTTGAGTCAATGATTTC-3′(AP-1-8)
TCTCTTGTAT TCTGTTGGTG AAGCTTGTAT CTACAGCTCC TTGTCTTTTC TTTTGATTTT

CTATGTCCAG GGTTGTTTCC ATGTGTTCTT TCTTGATTGC TTCTATTTCC ATTTTTAATT

CCTTCAACTG TTTGATTGTG TTTTCCTGGA AATCTTTCAG GGATTTTTGC GATTCCTCTC

TGTAGGCTTC TACTTGTTCT CTAAGGGAGT TCTTTATGTC TTTCTTGAAG TCCTCCAGCA

TCATGATCAA ATATGATTTT GAAACTAGGT CTTGCTTTTC TGGTGTGTTT GGATATTCCG
5′-TGTGTTTGGATATTCCGTGTTT-3′(NF-κBp65-5)
TGTTTGCTTT GGTGGGAGAA TTAGACTCCG ATGATGCCAT GTAGTCTTGG TTTCTGTTGC

TTGGGTTCCT GCGCTTGCCT CTCGCCATCA GATTATCTCT AGTGTTACTT TGTTCTGCTA

TTTCTGACAG TGGCTAGACT GTCCTATAAG CCTGTGTGTC AGGAGTGCTG TAGTCCTGTT

TTCCTGTTTT CTTTCAGCCA GTTATGGGGA CAGAGTGTTC TGCTTTCGGG CGTGTAGTTT

TTCCTCTGTA CAGGTCTTCA GCTGTTCCTG TGGGCCTGTG TCTTGAGTTC ACCAAGCAGG

TTTCTTGCAG GGGAAAATTT GGTCCTACCG GTGGTTCCAA GGCTCAAGTT TGCTCGTGGG

GTACTGCCTA AGTCCTCCCC GCGGCGGCAG CAACCGGGTA GATCTGTGCT GCTCTTTCCG

GGAGCCTCCA TGCACCAGGG TTCCAGATGA CGTTTGGTGT TTTCCTCTGG CATCTGGATG
5′-ACGTTTGGTGTTTTCCTCTGGC-3′(NF-κBp65-7)
