# Supplementary figures and images for: NF-κB and AP-1 are required for the lipopolysaccharide-induced expression of MCP-1, CXCL1, and Cx43 in cultured rat dorsal spinal cord astrocytes (part 1 of 2)
Source: Front Mol Neurosci. 2022 Jul 28;15:859558. doi: 10.3389/fnmol.2022.859558 (PMC9368326; doi:10.3389/fnmol.2022.859558)

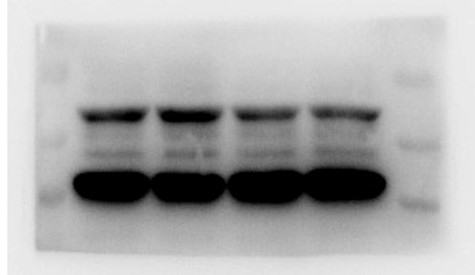

Supplement: Supplementary file 2 [file Data_Sheet_2.ZIP › WB original image/Fig.10/Fig.10D/Cx43/Cx43.jpg]

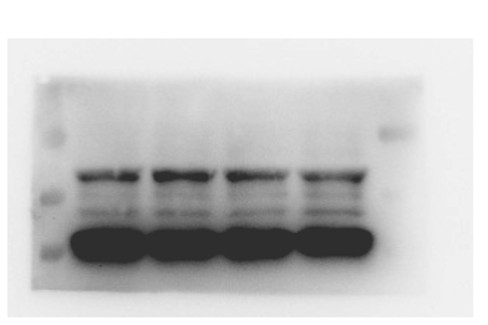

Supplement: Supplementary file 2 [file Data_Sheet_2.ZIP › WB original image/Fig.10/Fig.10D/p-Cx43/p-Cx43.jpg]

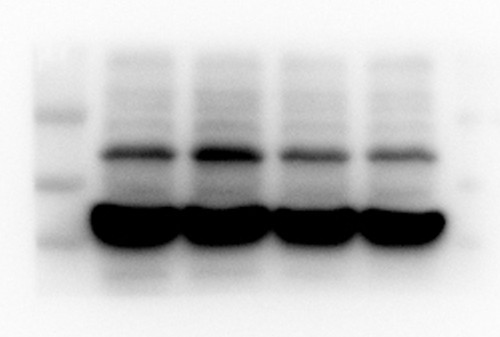

Supplement: Supplementary file 2 [file Data_Sheet_2.ZIP › WB original image/Fig.10/Fig.10E/Cx43/Cx43.jpg]

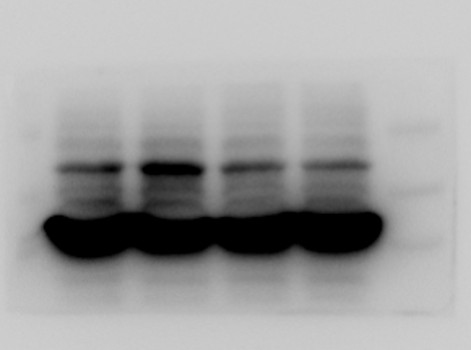

Supplement: Supplementary file 2 [file Data_Sheet_2.ZIP › WB original image/Fig.10/Fig.10E/p-Cx43/p-Cx43.jpg]

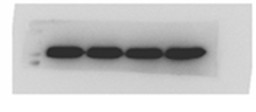

Supplement: Supplementary file 2 [file Data_Sheet_2.ZIP › WB original image/Fig.2/CXCL1/beta-actin.jpg]

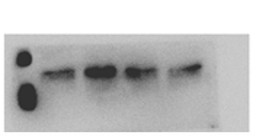

Supplement: Supplementary file 2 [file Data_Sheet_2.ZIP › WB original image/Fig.2/CXCL1/CXCL1.jpg]

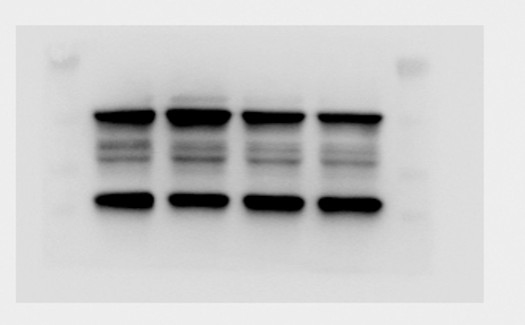

Supplement: Supplementary file 2 [file Data_Sheet_2.ZIP › WB original image/Fig.2/GFAP/GFAP.jpg]

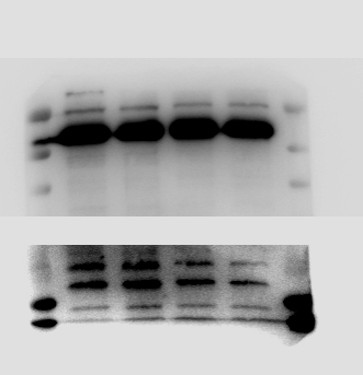

Supplement: Supplementary file 2 [file Data_Sheet_2.ZIP › WB original image/Fig.2/MCP-1/MCP-1.jpg]

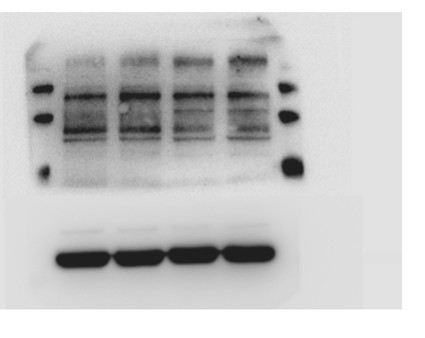

Supplement: Supplementary file 2 [file Data_Sheet_2.ZIP › WB original image/Fig.2/TLR4/TLR4.jpg]

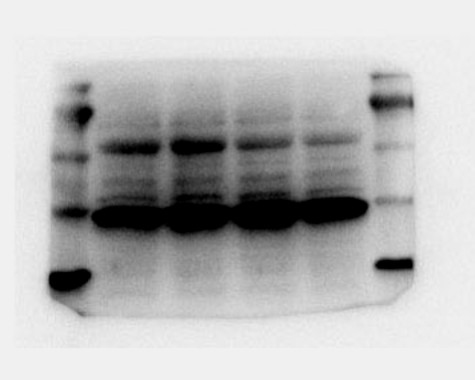

Supplement: Supplementary file 2 [file Data_Sheet_2.ZIP › WB original image/Fig.3/NF-a╩Bp65/NF-a╩Bp65.jpg]

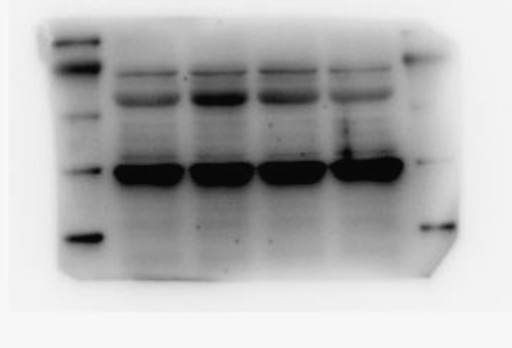

Supplement: Supplementary file 2 [file Data_Sheet_2.ZIP › WB original image/Fig.3/p-NF-a╩Bp65/p-NF-a╩Bp65.jpg]

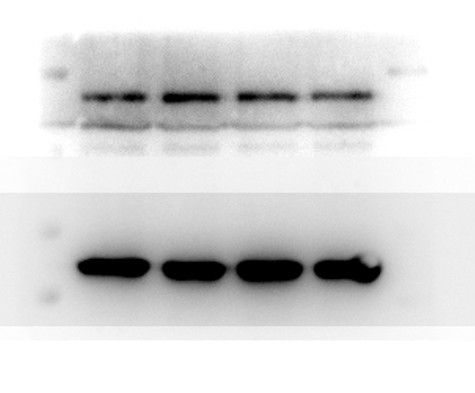

Supplement: Supplementary file 2 [file Data_Sheet_2.ZIP › WB original image/Fig.4/IKKa┴/IKKa┴.jpg]

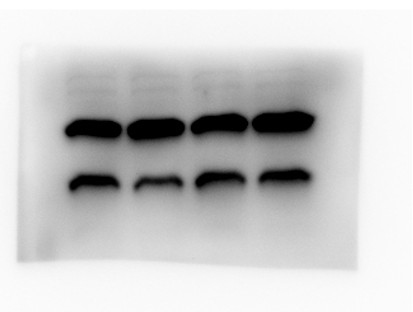

Supplement: Supplementary file 2 [file Data_Sheet_2.ZIP › WB original image/Fig.4/Ia╩B-a┴/═╝╞1⁄41.jpg]

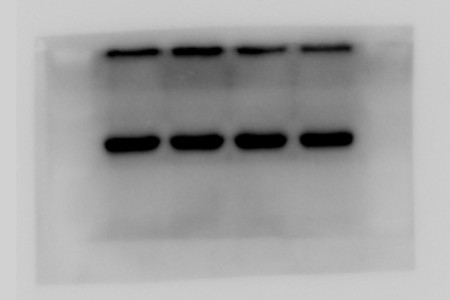

Supplement: Supplementary file 2 [file Data_Sheet_2.ZIP › WB original image/Fig.4/NF-a╩Bp50/NF-a╩Bp50.jpg]

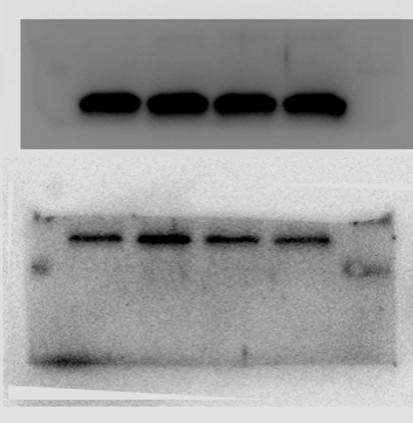

Supplement: Supplementary file 2 [file Data_Sheet_2.ZIP › WB original image/Fig.5/AP-1/AP-1.jpg]

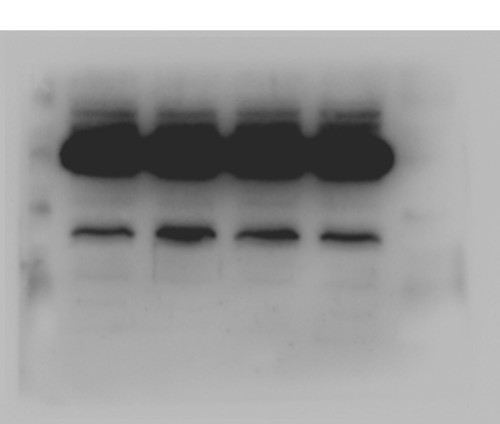

Supplement: Supplementary file 2 [file Data_Sheet_2.ZIP › WB original image/Fig.5/p-AP-1/p-AP-1.jpg]

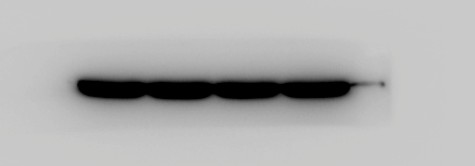

Supplement: Supplementary file 2 [file Data_Sheet_2.ZIP › WB original image/Fig.6/CXCL1/beta-actin.jpg]

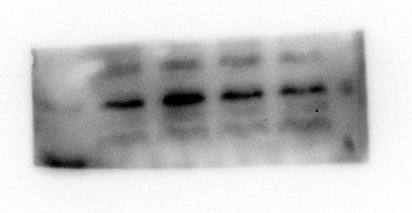

Supplement: Supplementary file 2 [file Data_Sheet_2.ZIP › WB original image/Fig.6/CXCL1/CXCL1.jpg]

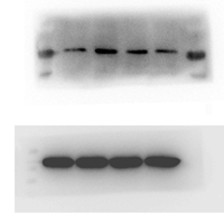

Supplement: Supplementary file 2 [file Data_Sheet_2.ZIP › WB original image/Fig.6/MCP-1/MCP-1.jpg]

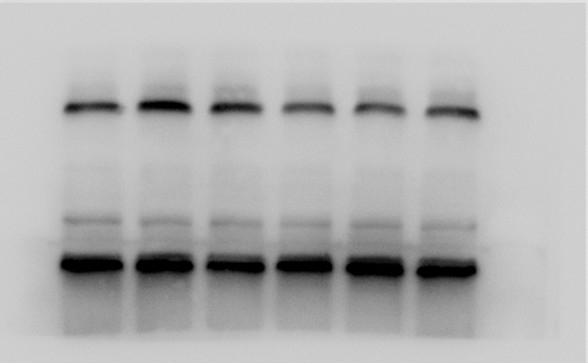

Supplement: Supplementary file 2 [file Data_Sheet_2.ZIP › WB original image/Fig.8/IKKa┴/IKKa┴.jpg]

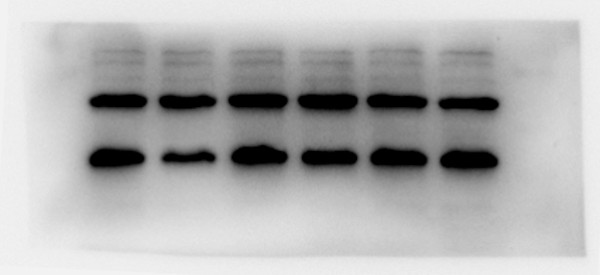

Supplement: Supplementary file 2 [file Data_Sheet_2.ZIP › WB original image/Fig.8/Ia╩B-a┴/Ia╩B-a┴.jpg]

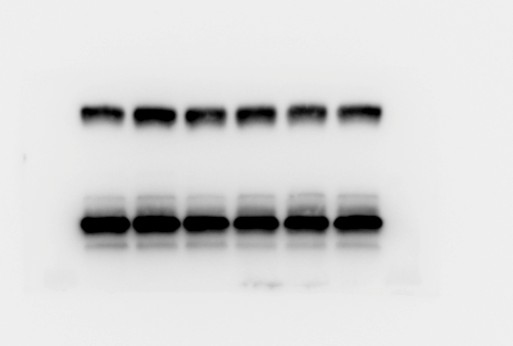

Supplement: Supplementary file 2 [file Data_Sheet_2.ZIP › WB original image/Fig.8/NF-a╩Bp50/NF-a╩Bp50.jpg]

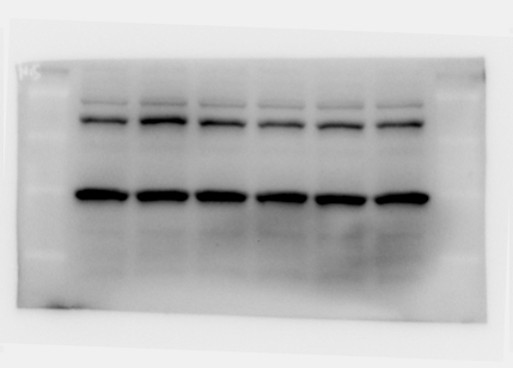

Supplement: Supplementary file 2 [file Data_Sheet_2.ZIP › WB original image/Fig.8/NF-a╩Bp65/NF-a╩Bp65.jpg]

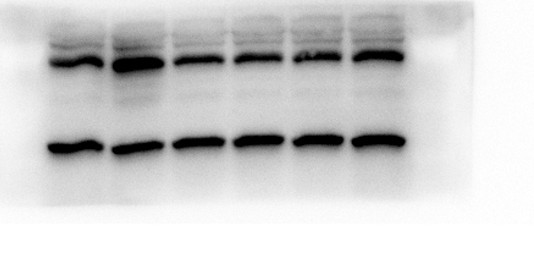

Supplement: Supplementary file 2 [file Data_Sheet_2.ZIP › WB original image/Fig.8/p-NF-a╩Bp65/p-NF-a╩Bp65.jpg]

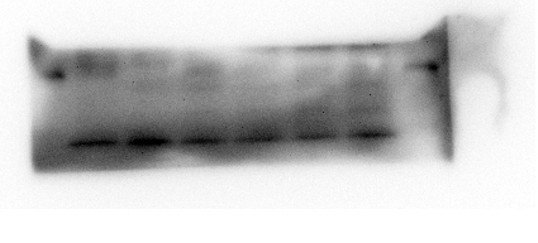

Supplement: Supplementary file 2 [file Data_Sheet_2.ZIP › WB original image/Fig.9/AP-1/AP-1.jpg]

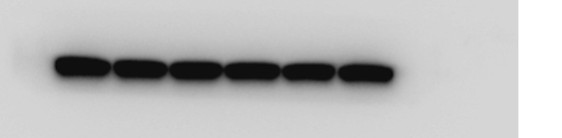

Supplement: Supplementary file 2 [file Data_Sheet_2.ZIP › WB original image/Fig.9/AP-1/beta-actin.jpg]

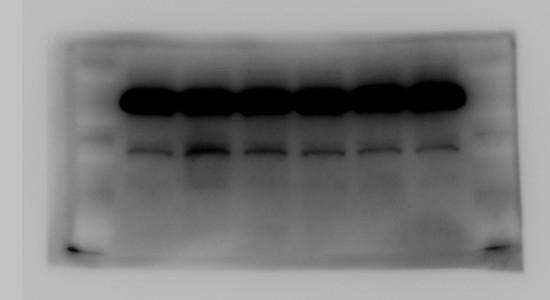

Supplement: Supplementary file 2 [file Data_Sheet_2.ZIP › WB original image/Fig.9/p-AP-1/p-AP-1.jpg]

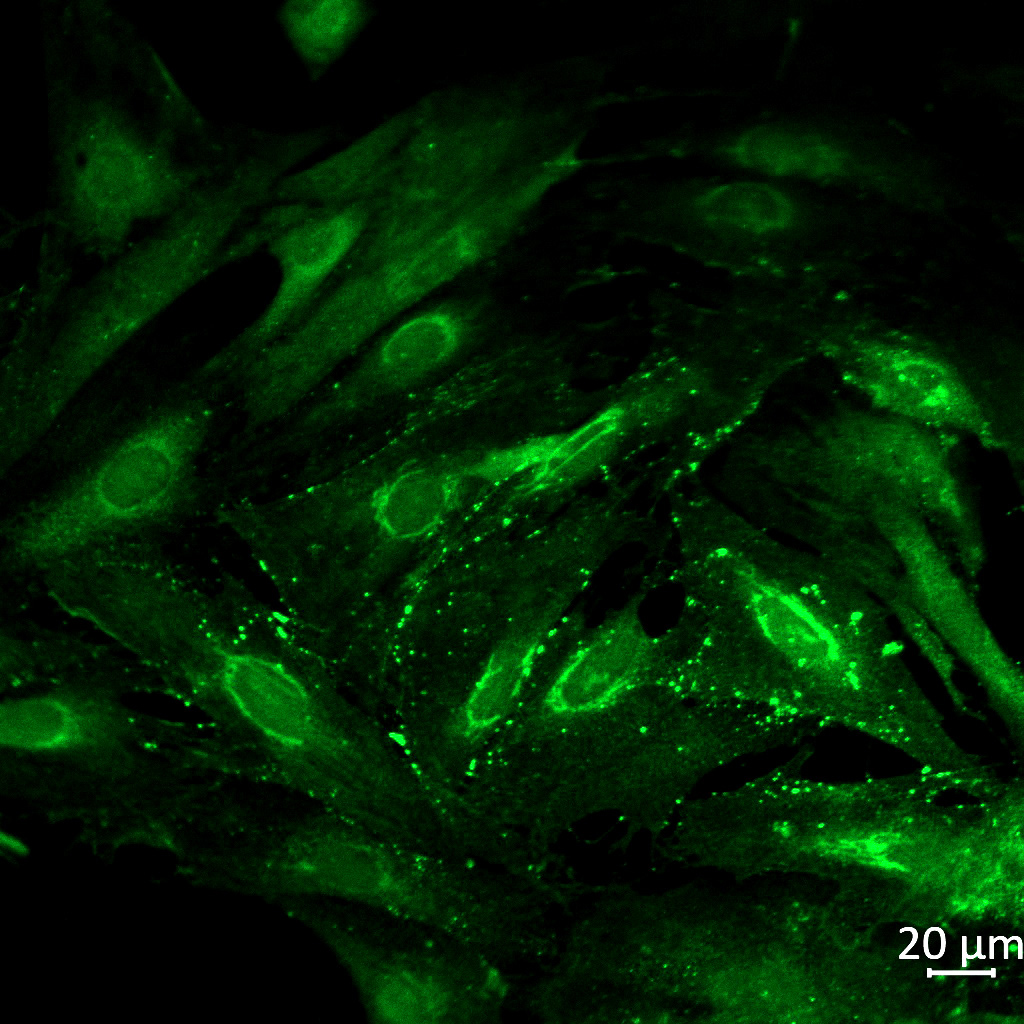

Supplement: Supplementary file 3 [file Data_Sheet_3.ZIP › Immunofluorescence original image/Fig.10 (Double immunofluorescence of TLR4 and Cx43)/Cx43 (green).jpg]

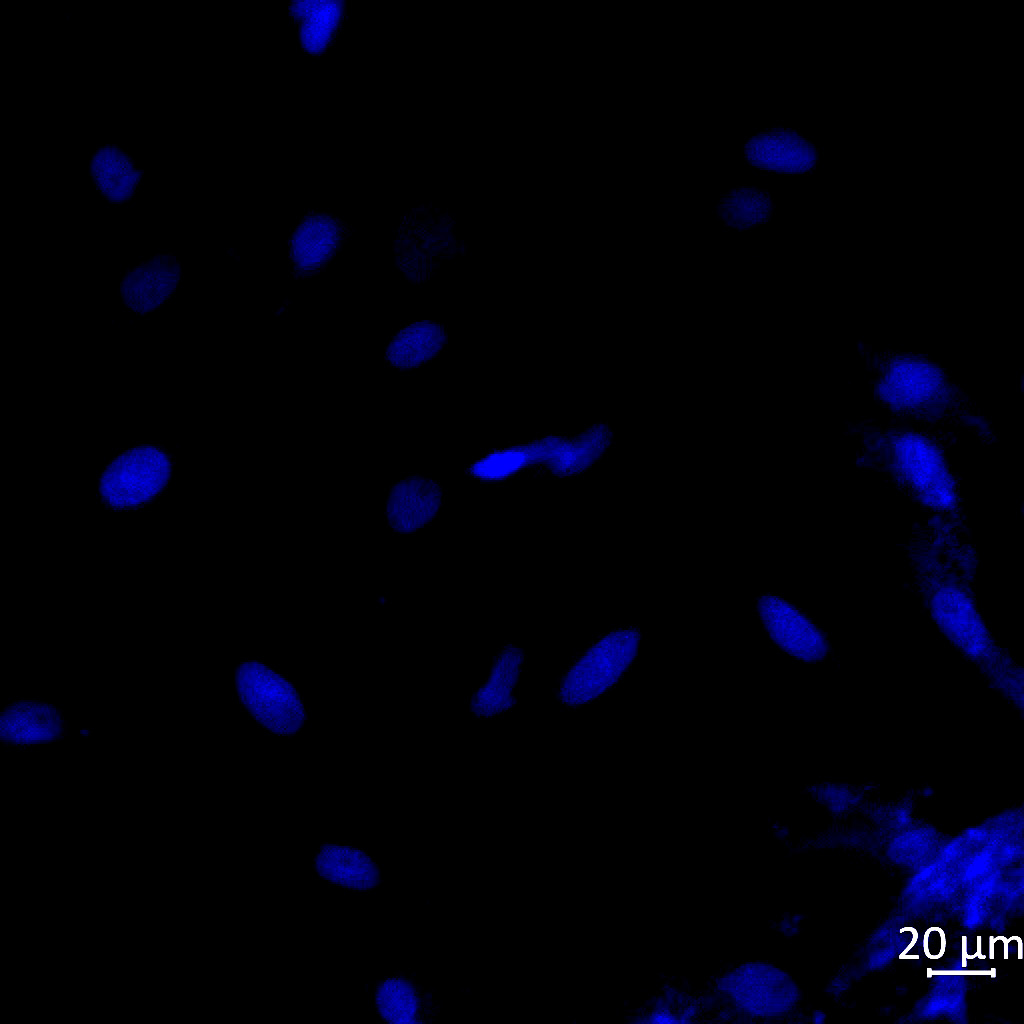

Supplement: Supplementary file 3 [file Data_Sheet_3.ZIP › Immunofluorescence original image/Fig.10 (Double immunofluorescence of TLR4 and Cx43)/DAPI (blue).jpg]

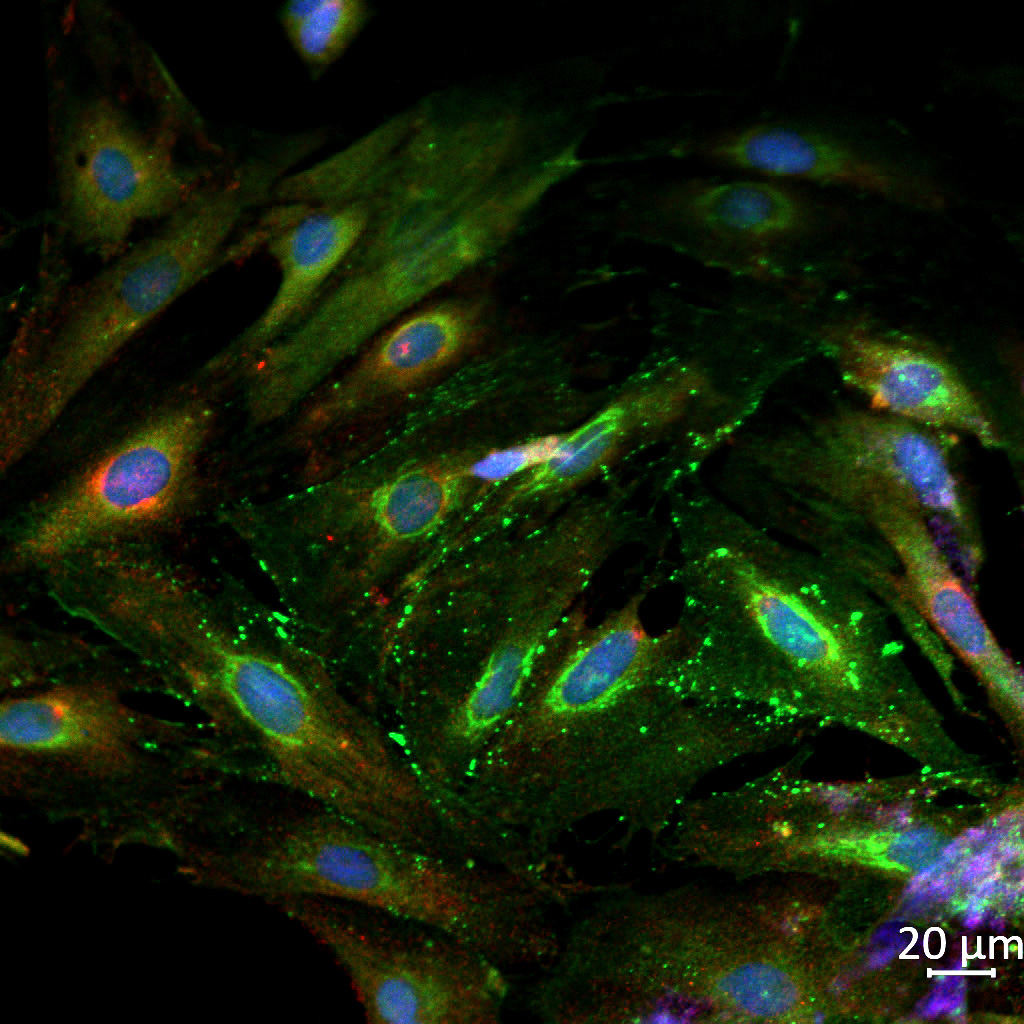

Supplement: Supplementary file 3 [file Data_Sheet_3.ZIP › Immunofluorescence original image/Fig.10 (Double immunofluorescence of TLR4 and Cx43)/merge.jpg]

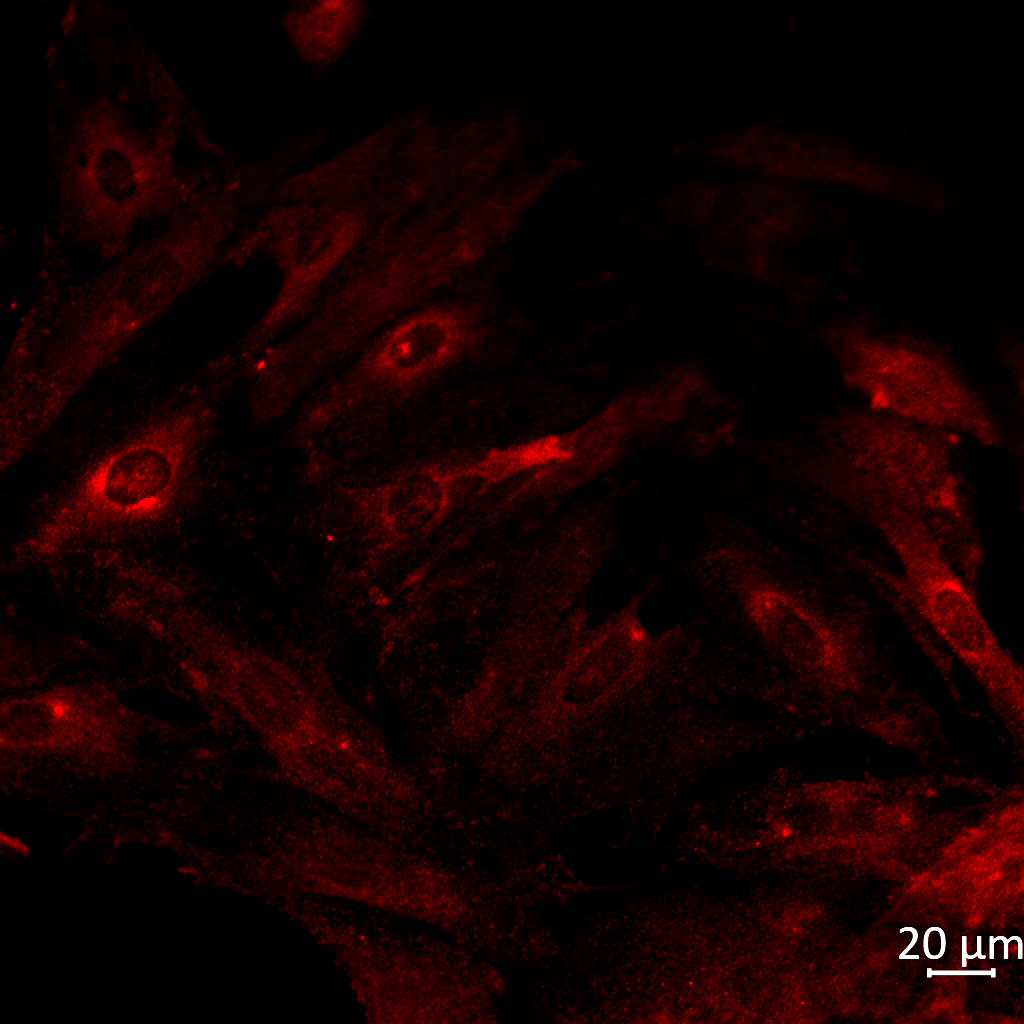

Supplement: Supplementary file 3 [file Data_Sheet_3.ZIP › Immunofluorescence original image/Fig.10 (Double immunofluorescence of TLR4 and Cx43)/TLR4 (red).jpg]

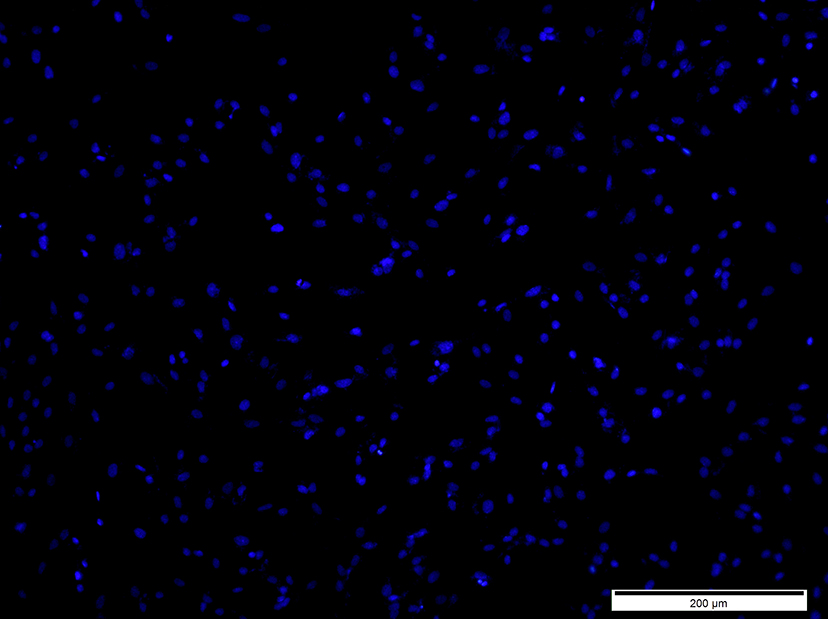

Supplement: Supplementary file 3 [file Data_Sheet_3.ZIP › Immunofluorescence original image/Fig.2 GFAP/DAPI (blue).jpg]

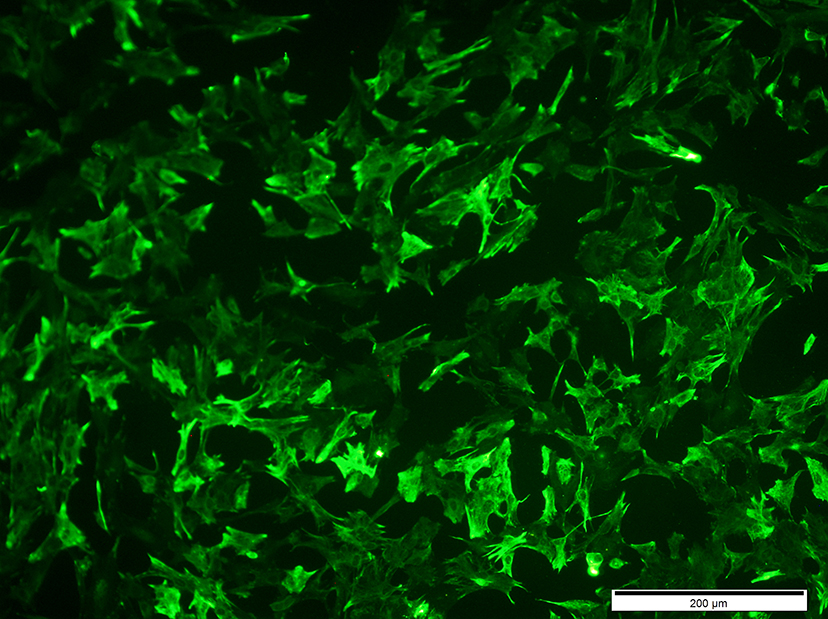

Supplement: Supplementary file 3 [file Data_Sheet_3.ZIP › Immunofluorescence original image/Fig.2 GFAP/GFAP(green).jpg]

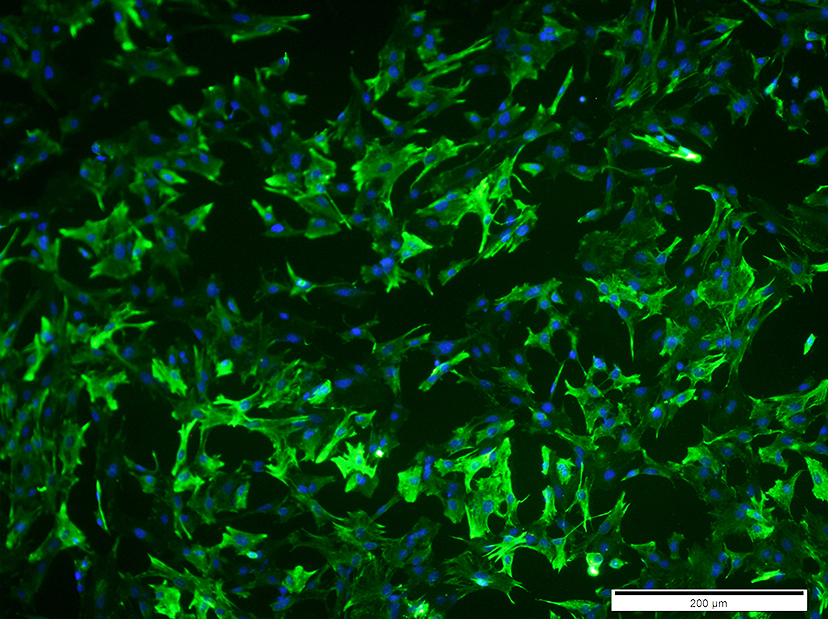

Supplement: Supplementary file 3 [file Data_Sheet_3.ZIP › Immunofluorescence original image/Fig.2 GFAP/merge.jpg]

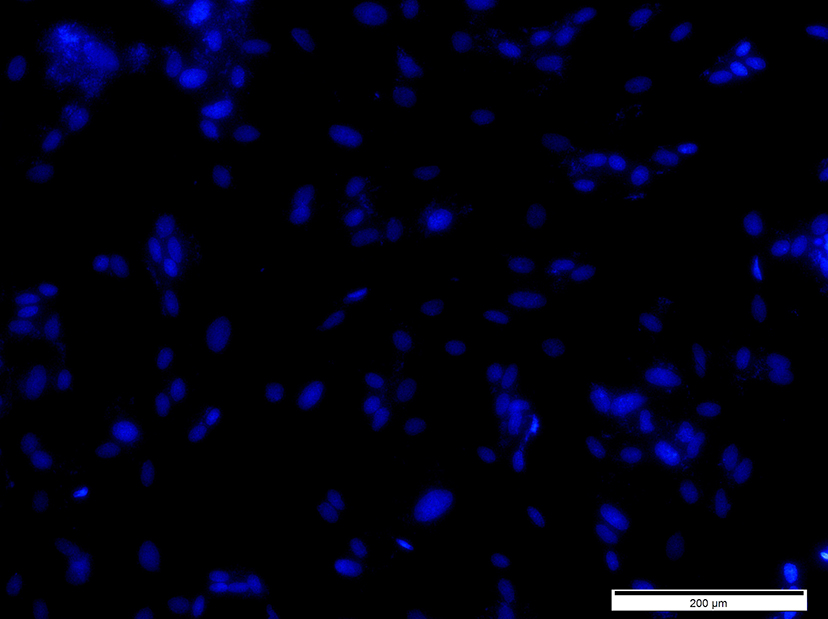

Supplement: Supplementary file 3 [file Data_Sheet_3.ZIP › Immunofluorescence original image/Fig.3 (nuclear translocation of NF-a╩Bp65)/con group/DAPI (blue).jpg]

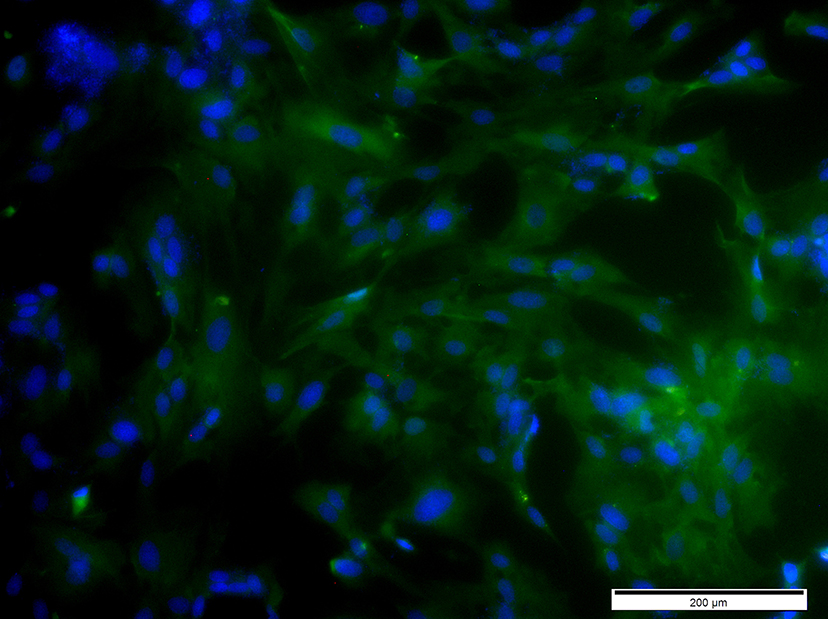

Supplement: Supplementary file 3 [file Data_Sheet_3.ZIP › Immunofluorescence original image/Fig.3 (nuclear translocation of NF-a╩Bp65)/con group/merge.jpg]

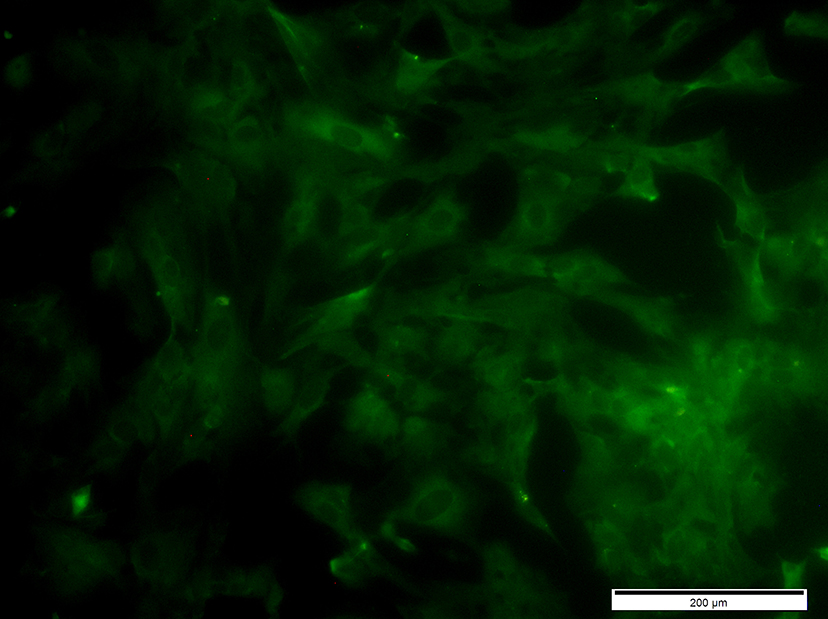

Supplement: Supplementary file 3 [file Data_Sheet_3.ZIP › Immunofluorescence original image/Fig.3 (nuclear translocation of NF-a╩Bp65)/con group/NF-a╩Bp65 (green).jpg]

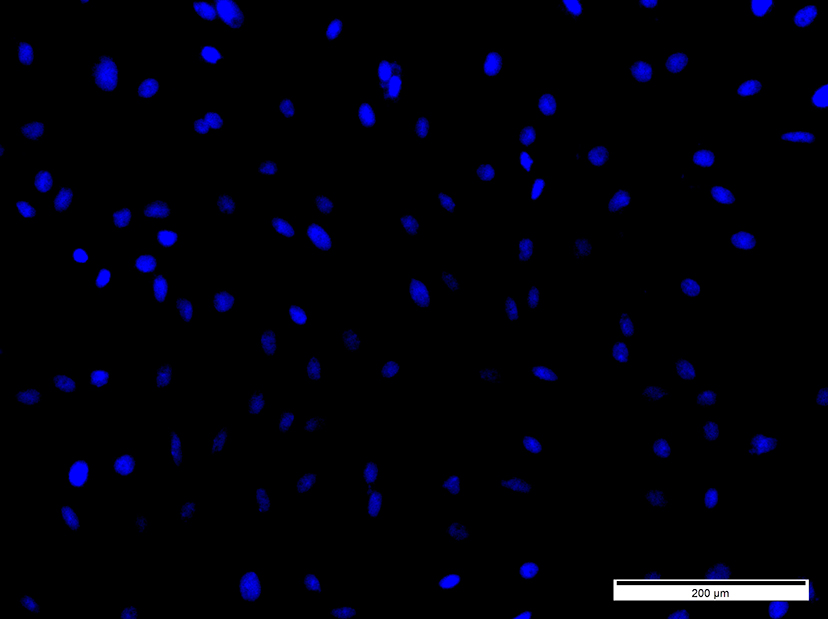

Supplement: Supplementary file 3 [file Data_Sheet_3.ZIP › Immunofluorescence original image/Fig.3 (nuclear translocation of NF-a╩Bp65)/LPS group/DAPI (blue).jpg]

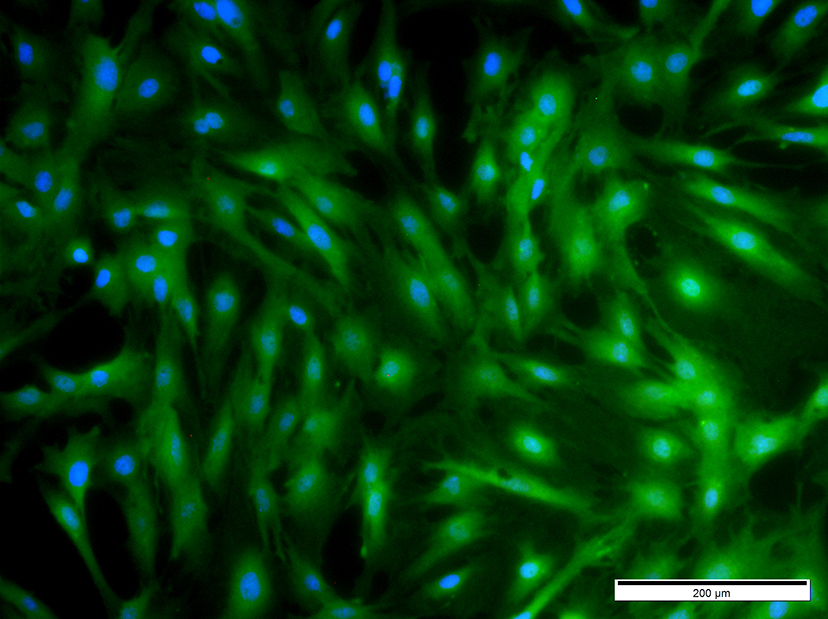

Supplement: Supplementary file 3 [file Data_Sheet_3.ZIP › Immunofluorescence original image/Fig.3 (nuclear translocation of NF-a╩Bp65)/LPS group/merge.jpg]

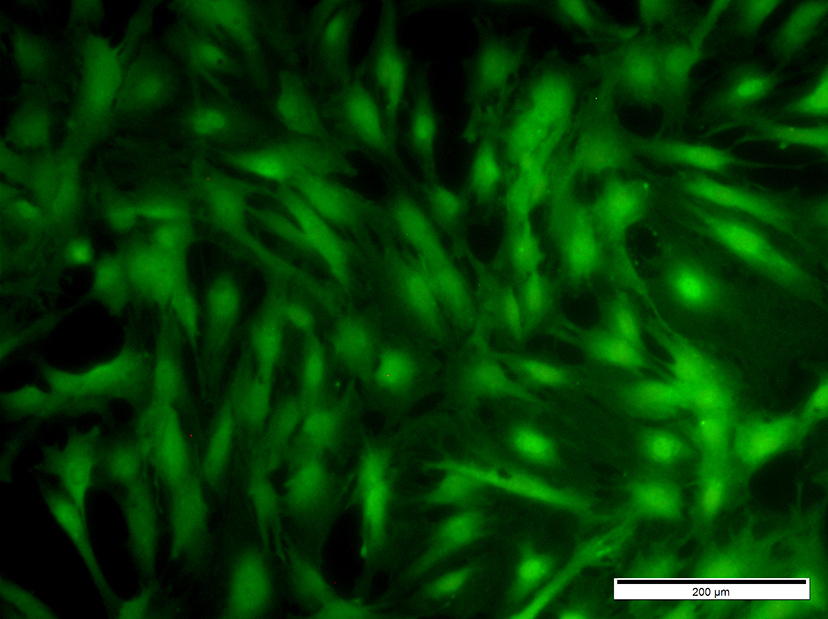

Supplement: Supplementary file 3 [file Data_Sheet_3.ZIP › Immunofluorescence original image/Fig.3 (nuclear translocation of NF-a╩Bp65)/LPS group/NF-a╩Bp65 (green).jpg]

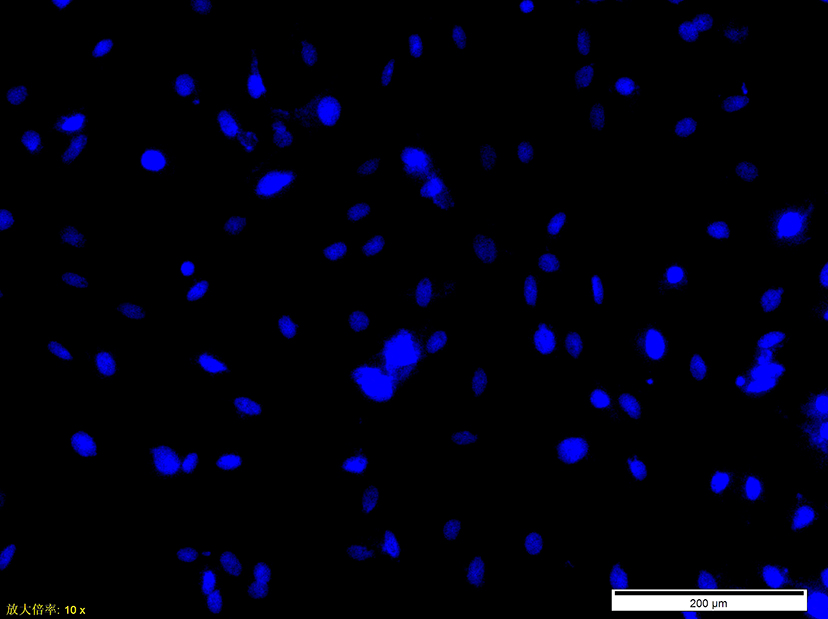

Supplement: Supplementary file 3 [file Data_Sheet_3.ZIP › Immunofluorescence original image/Fig.3 (nuclear translocation of NF-a╩Bp65)/LPS+TAK-242 group/DAPI (blue).jpg]

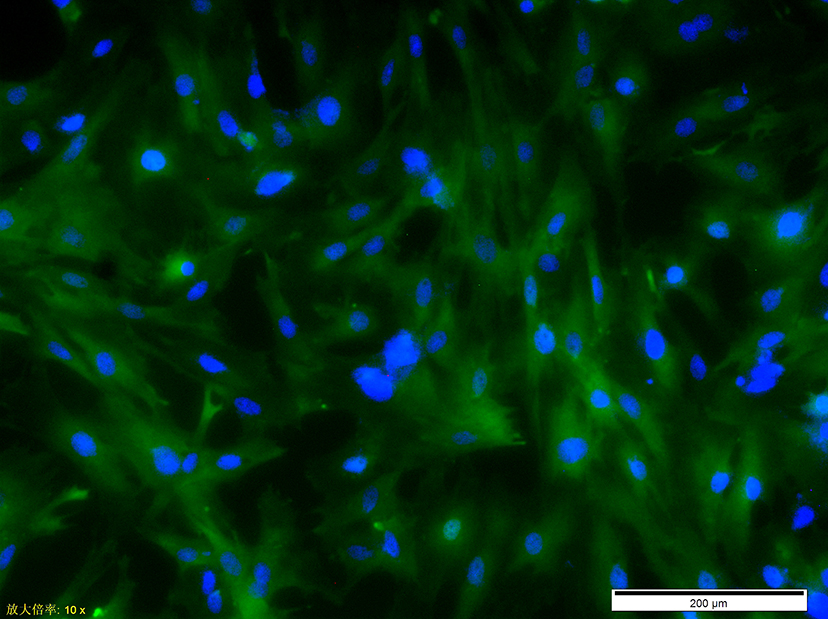

Supplement: Supplementary file 3 [file Data_Sheet_3.ZIP › Immunofluorescence original image/Fig.3 (nuclear translocation of NF-a╩Bp65)/LPS+TAK-242 group/merge.jpg]

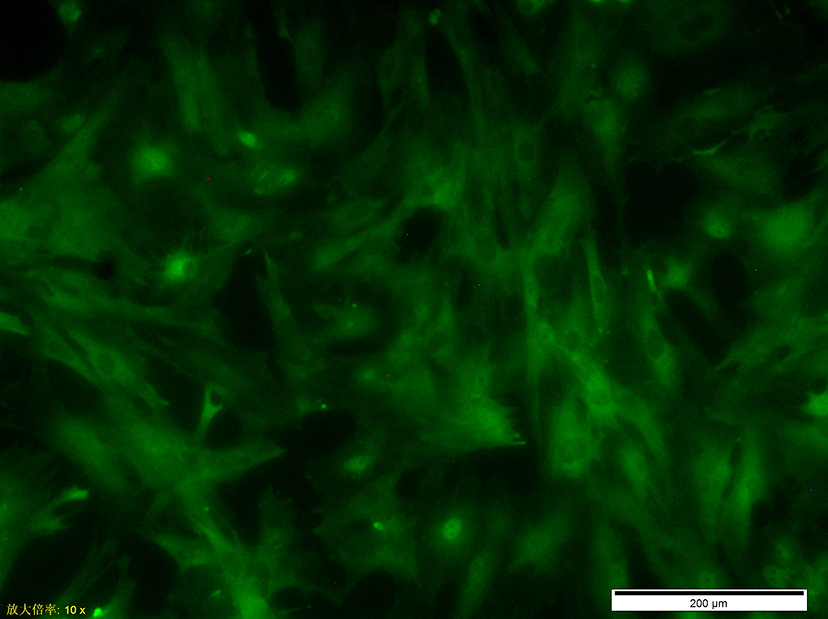

Supplement: Supplementary file 3 [file Data_Sheet_3.ZIP › Immunofluorescence original image/Fig.3 (nuclear translocation of NF-a╩Bp65)/LPS+TAK-242 group/NF-a╩Bp65 (green).jpg]

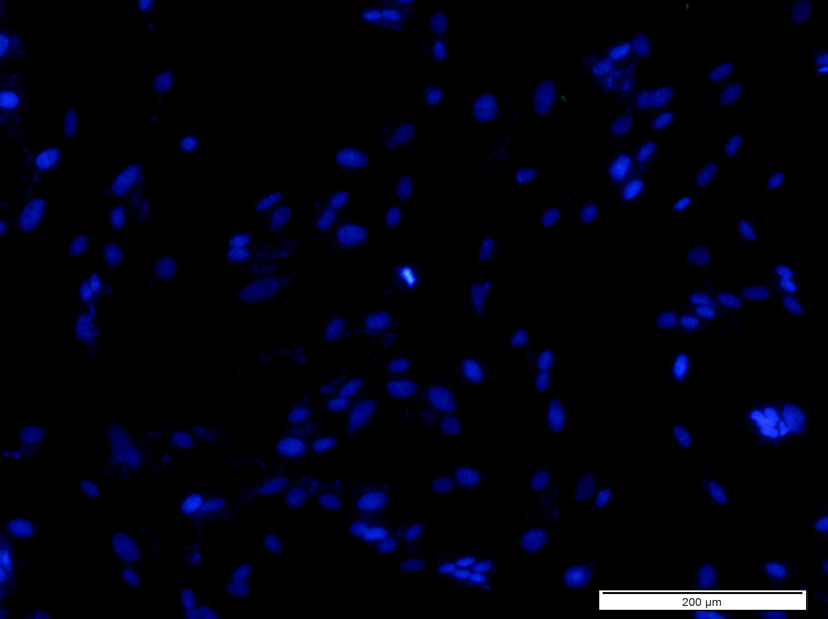

Supplement: Supplementary file 3 [file Data_Sheet_3.ZIP › Immunofluorescence original image/Fig.4 (nuclear translocation of NF-a╩Bp50)/con group/DAPI (blue).jpg]

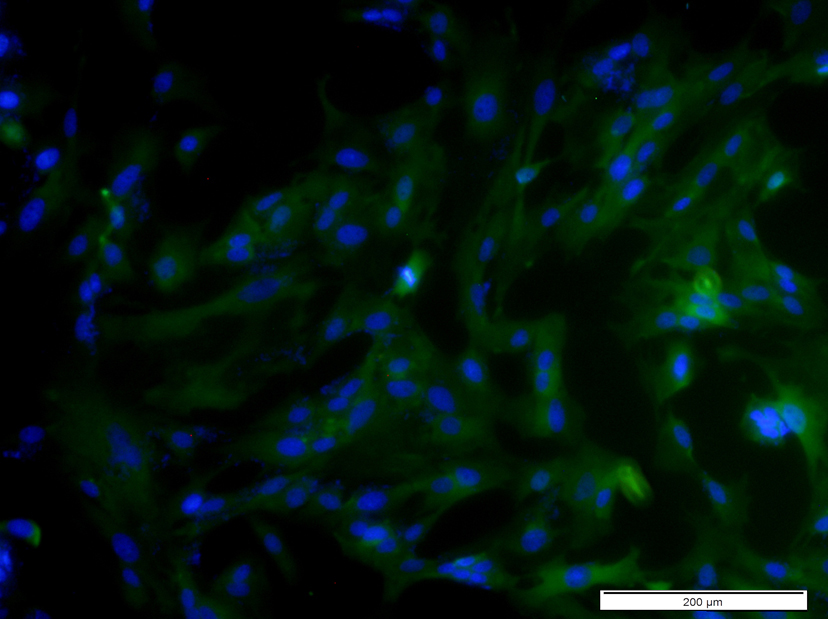

Supplement: Supplementary file 3 [file Data_Sheet_3.ZIP › Immunofluorescence original image/Fig.4 (nuclear translocation of NF-a╩Bp50)/con group/merge.jpg]

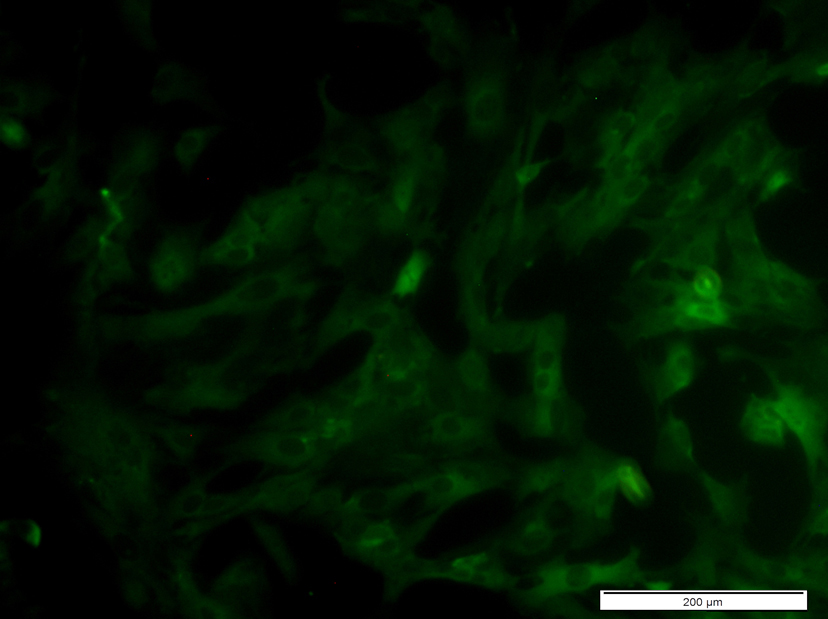

Supplement: Supplementary file 3 [file Data_Sheet_3.ZIP › Immunofluorescence original image/Fig.4 (nuclear translocation of NF-a╩Bp50)/con group/NF-a╩Bp50 (green).jpg]

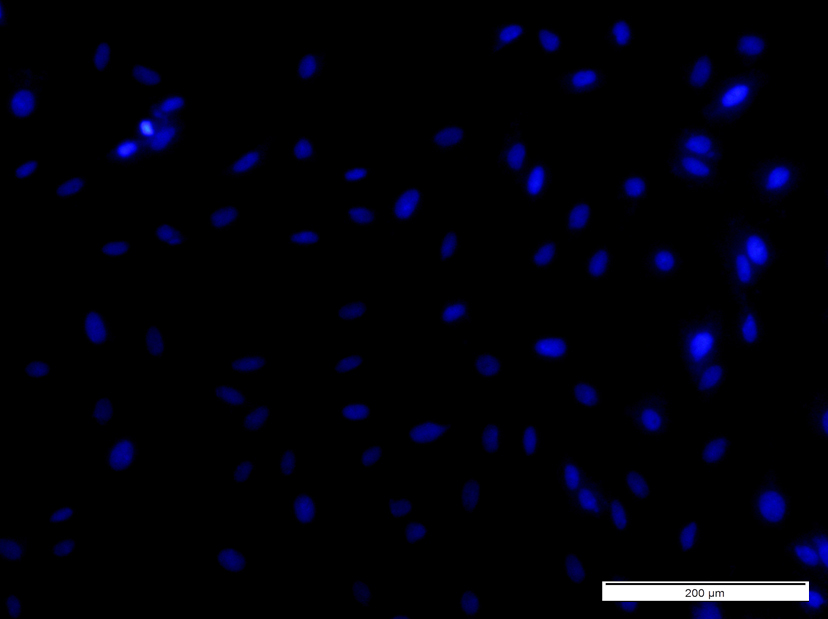

Supplement: Supplementary file 3 [file Data_Sheet_3.ZIP › Immunofluorescence original image/Fig.4 (nuclear translocation of NF-a╩Bp50)/LPS group/DAPI (blue).jpg]

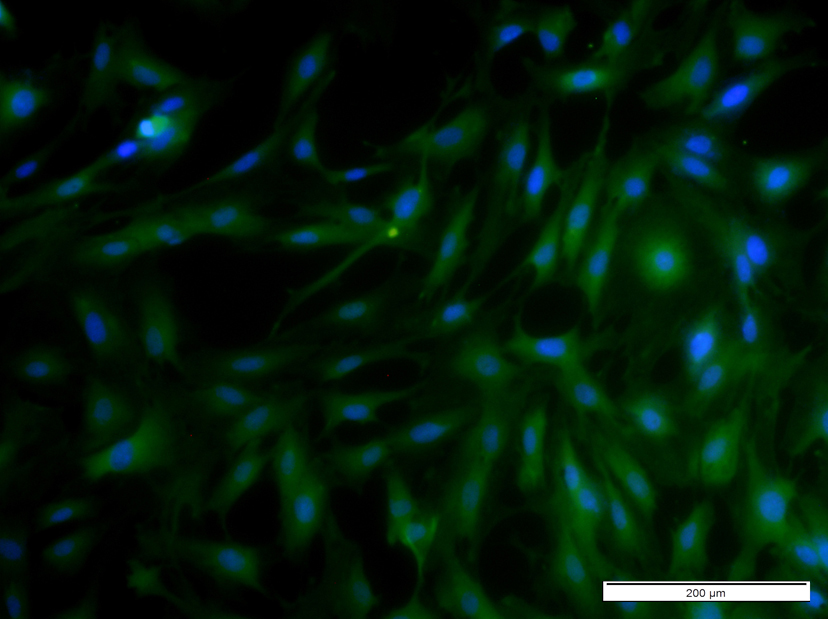

Supplement: Supplementary file 3 [file Data_Sheet_3.ZIP › Immunofluorescence original image/Fig.4 (nuclear translocation of NF-a╩Bp50)/LPS group/merge.jpg]

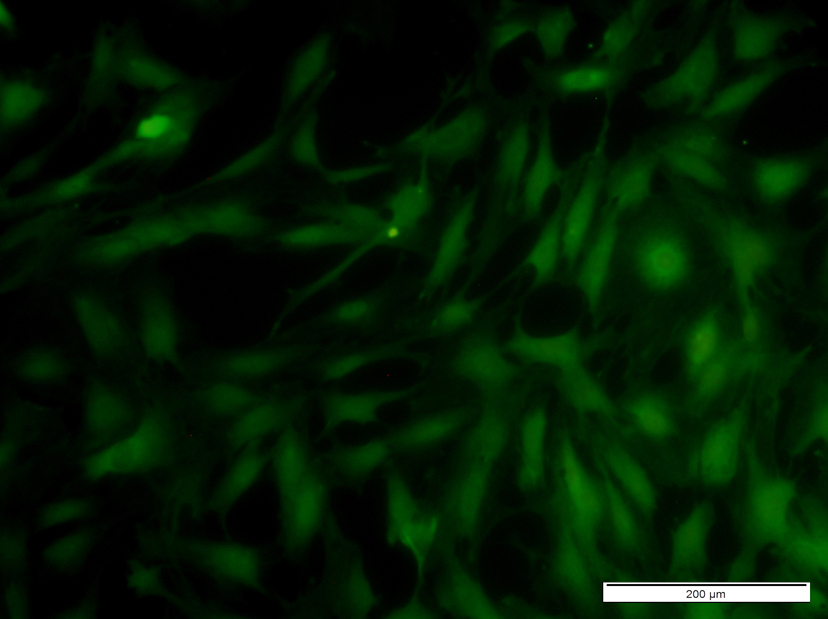

Supplement: Supplementary file 3 [file Data_Sheet_3.ZIP › Immunofluorescence original image/Fig.4 (nuclear translocation of NF-a╩Bp50)/LPS group/NF-a╩Bp50 (green).jpg]

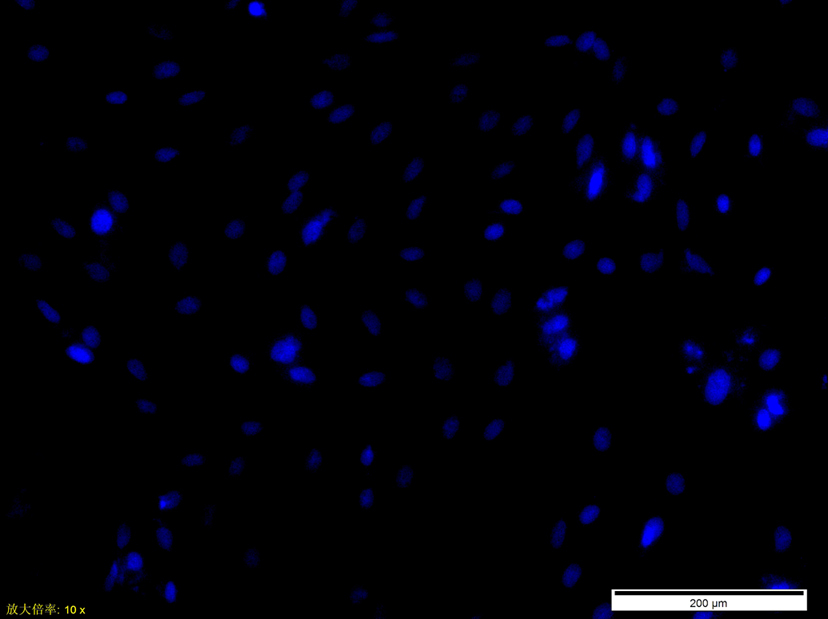

Supplement: Supplementary file 3 [file Data_Sheet_3.ZIP › Immunofluorescence original image/Fig.4 (nuclear translocation of NF-a╩Bp50)/LPS+TAK-242 group/DAPI (blue).jpg]

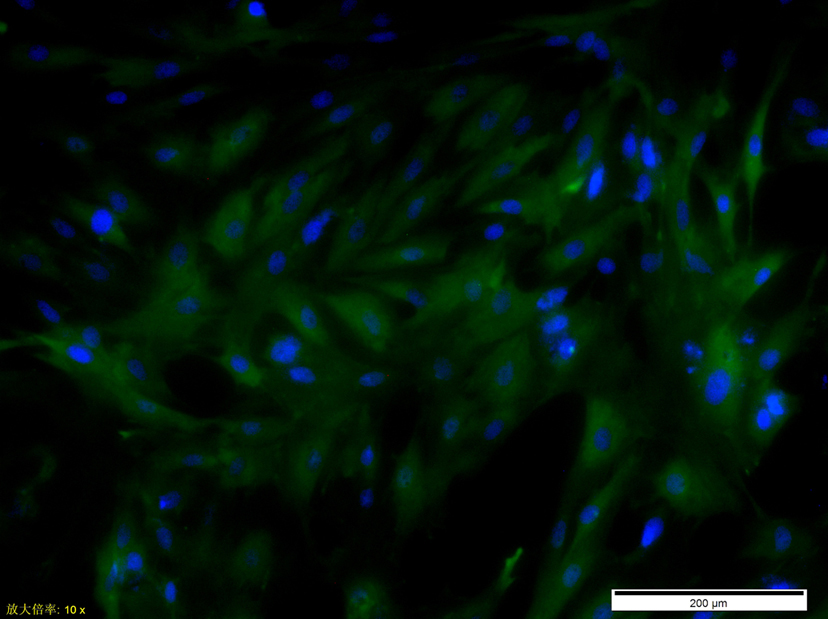

Supplement: Supplementary file 3 [file Data_Sheet_3.ZIP › Immunofluorescence original image/Fig.4 (nuclear translocation of NF-a╩Bp50)/LPS+TAK-242 group/merge.jpg]

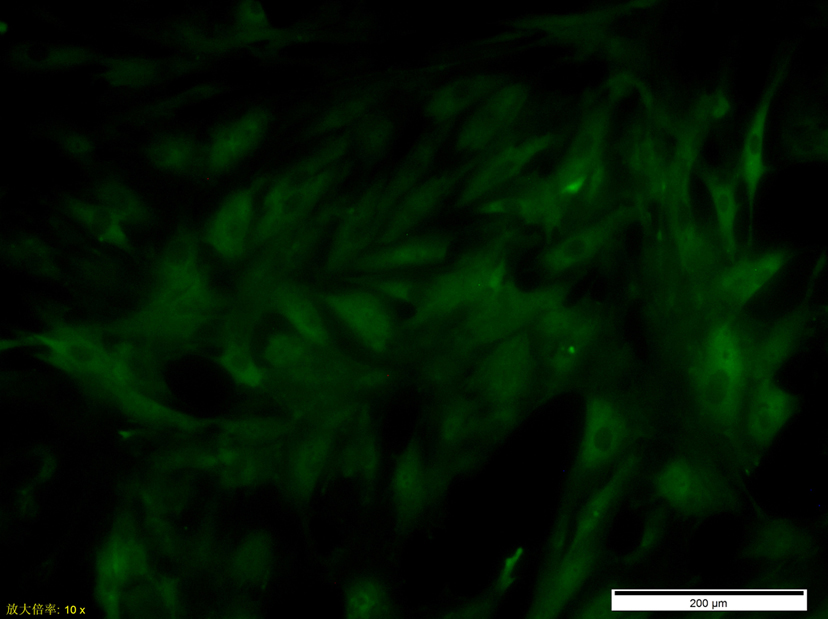

Supplement: Supplementary file 3 [file Data_Sheet_3.ZIP › Immunofluorescence original image/Fig.4 (nuclear translocation of NF-a╩Bp50)/LPS+TAK-242 group/NF-a╩Bp50 (green).jpg]

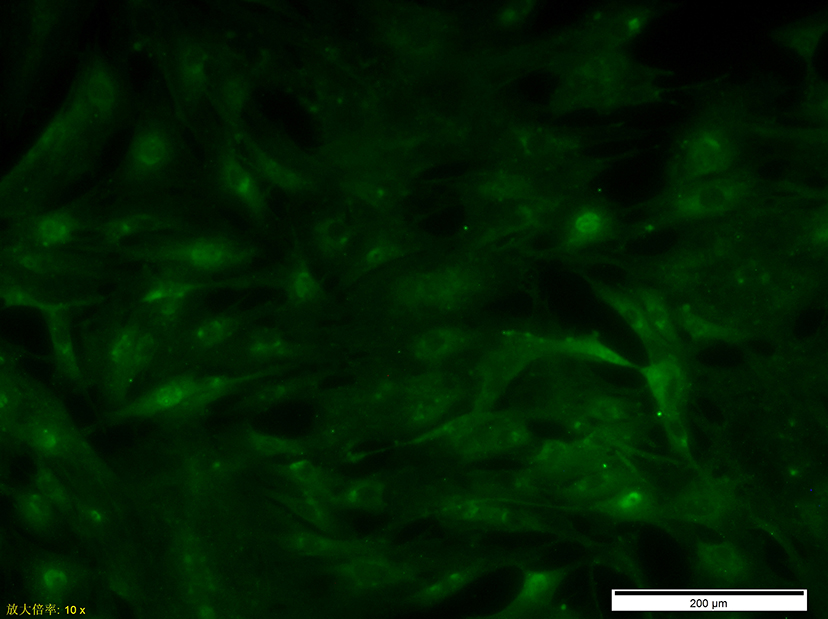

Supplement: Supplementary file 3 [file Data_Sheet_3.ZIP › Immunofluorescence original image/Fig.5 (nuclear translocation of AP-1)/con group/AP-1 (green).jpg]

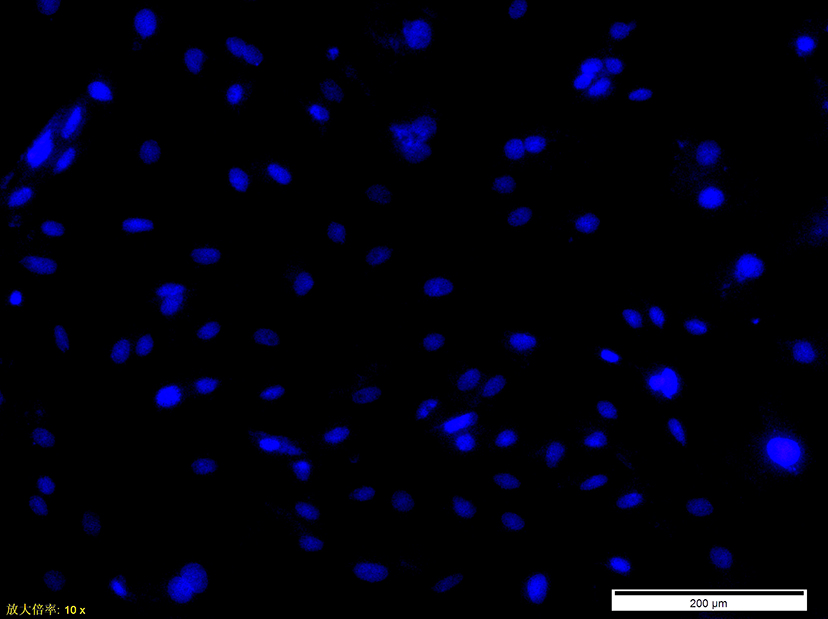

Supplement: Supplementary file 3 [file Data_Sheet_3.ZIP › Immunofluorescence original image/Fig.5 (nuclear translocation of AP-1)/con group/DAPI (blue).jpg]

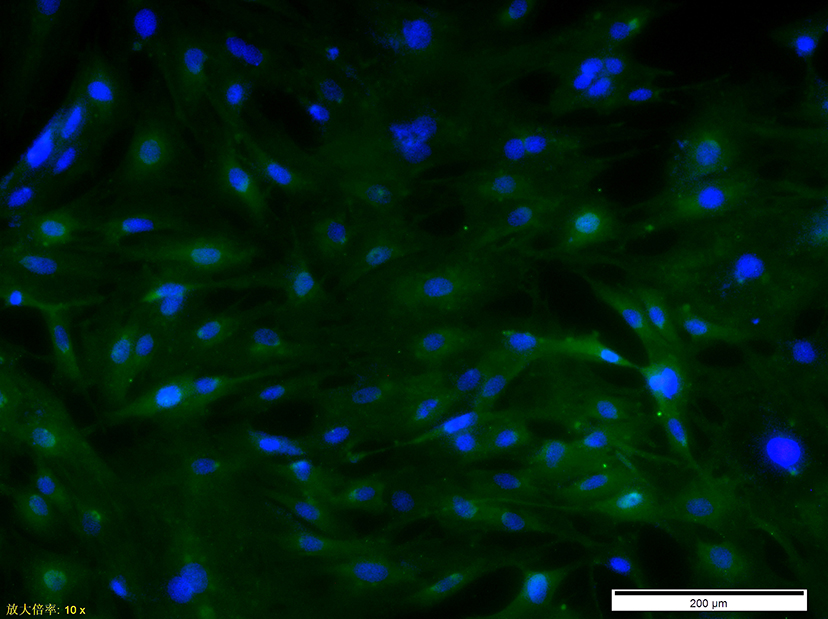

Supplement: Supplementary file 3 [file Data_Sheet_3.ZIP › Immunofluorescence original image/Fig.5 (nuclear translocation of AP-1)/con group/merge.jpg]

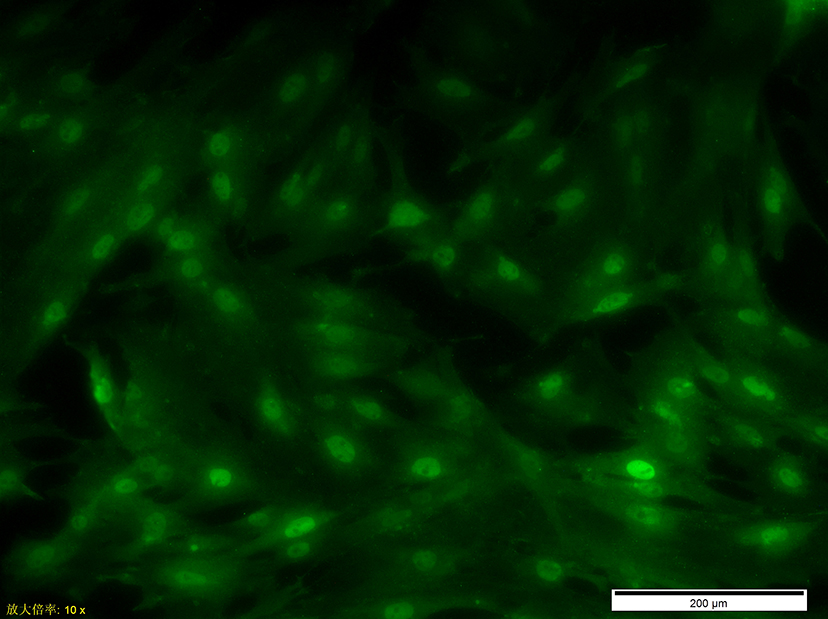

Supplement: Supplementary file 3 [file Data_Sheet_3.ZIP › Immunofluorescence original image/Fig.5 (nuclear translocation of AP-1)/LPS group/AP-1 (green).jpg]

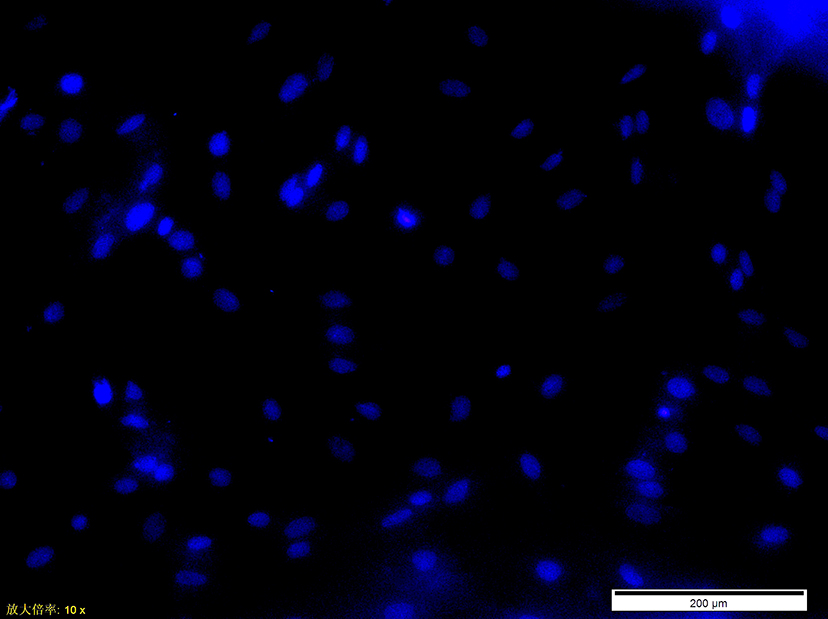

Supplement: Supplementary file 3 [file Data_Sheet_3.ZIP › Immunofluorescence original image/Fig.5 (nuclear translocation of AP-1)/LPS group/DAPI (blue).jpg]

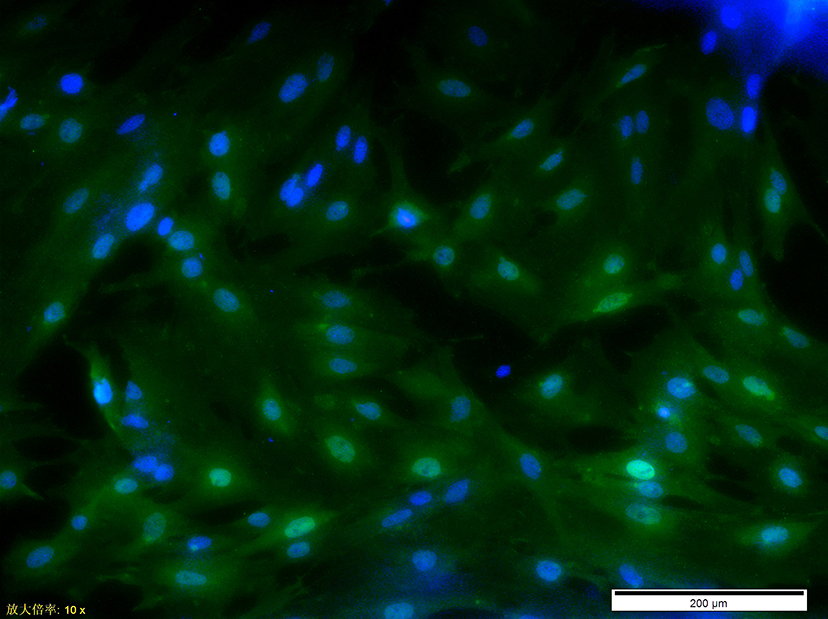

Supplement: Supplementary file 3 [file Data_Sheet_3.ZIP › Immunofluorescence original image/Fig.5 (nuclear translocation of AP-1)/LPS group/merge.jpg]

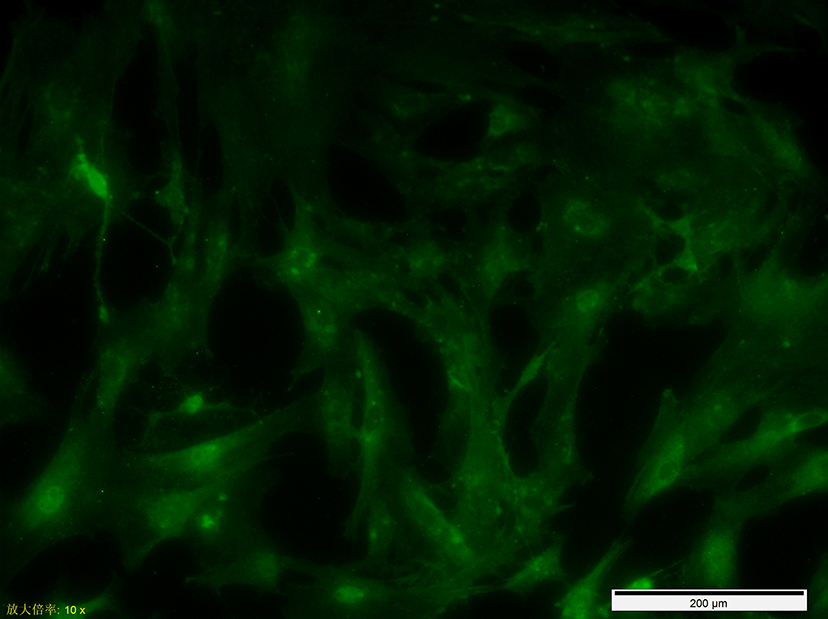

Supplement: Supplementary file 3 [file Data_Sheet_3.ZIP › Immunofluorescence original image/Fig.5 (nuclear translocation of AP-1)/LPS+TAK-242 group/AP-1 (green).jpg]

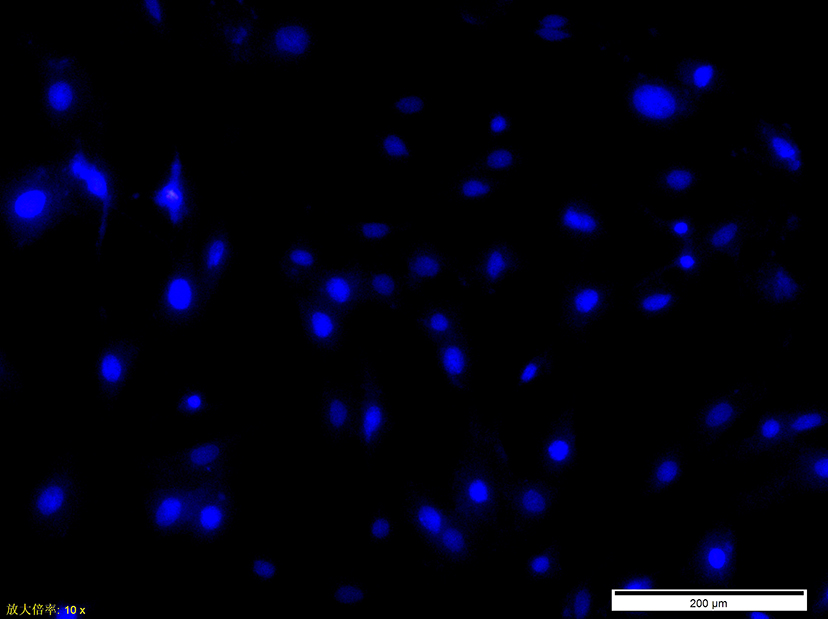

Supplement: Supplementary file 3 [file Data_Sheet_3.ZIP › Immunofluorescence original image/Fig.5 (nuclear translocation of AP-1)/LPS+TAK-242 group/DAPI (blue).jpg]

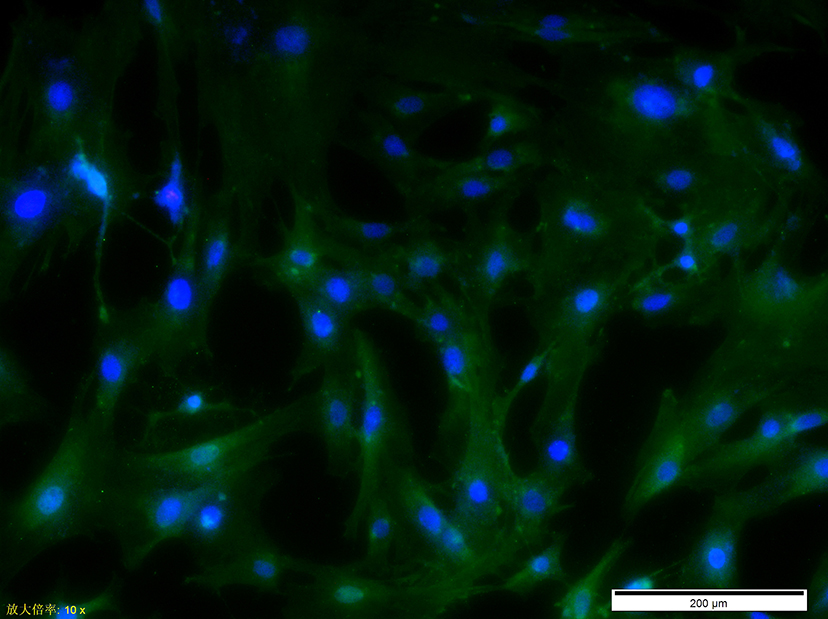

Supplement: Supplementary file 3 [file Data_Sheet_3.ZIP › Immunofluorescence original image/Fig.5 (nuclear translocation of AP-1)/LPS+TAK-242 group/merge.jpg]

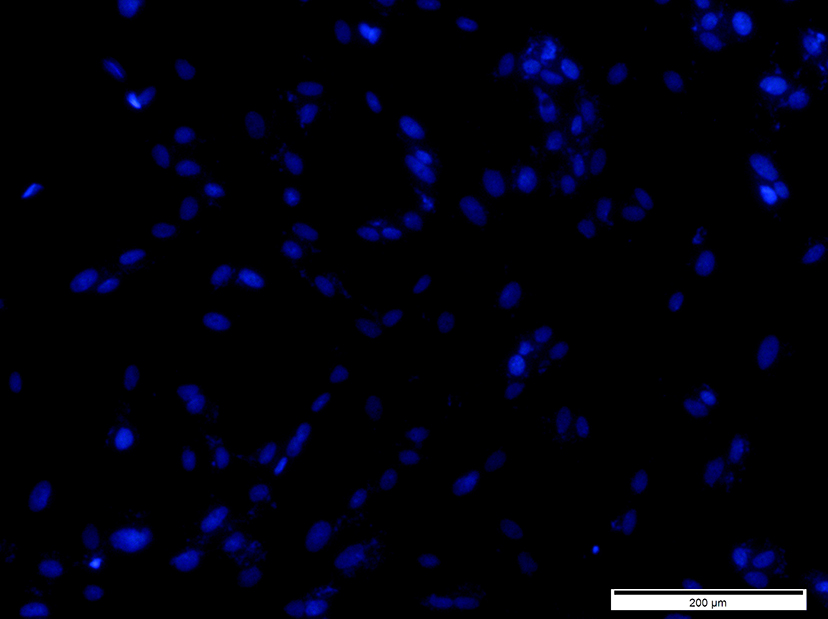

Supplement: Supplementary file 3 [file Data_Sheet_3.ZIP › Immunofluorescence original image/Fig.8 (nuclear translocation of NF-a╩Bp65)/con group/DAPI (blue).jpg]

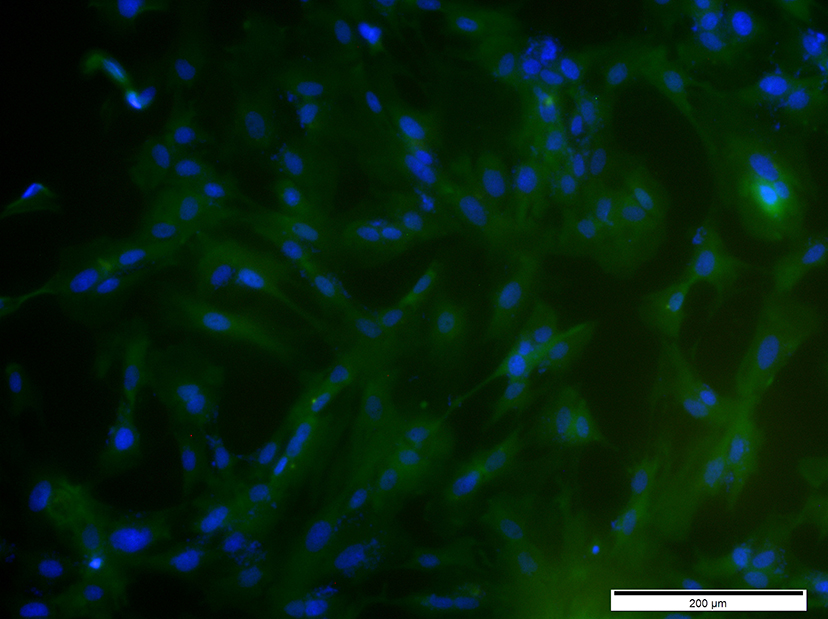

Supplement: Supplementary file 3 [file Data_Sheet_3.ZIP › Immunofluorescence original image/Fig.8 (nuclear translocation of NF-a╩Bp65)/con group/merge.jpg]

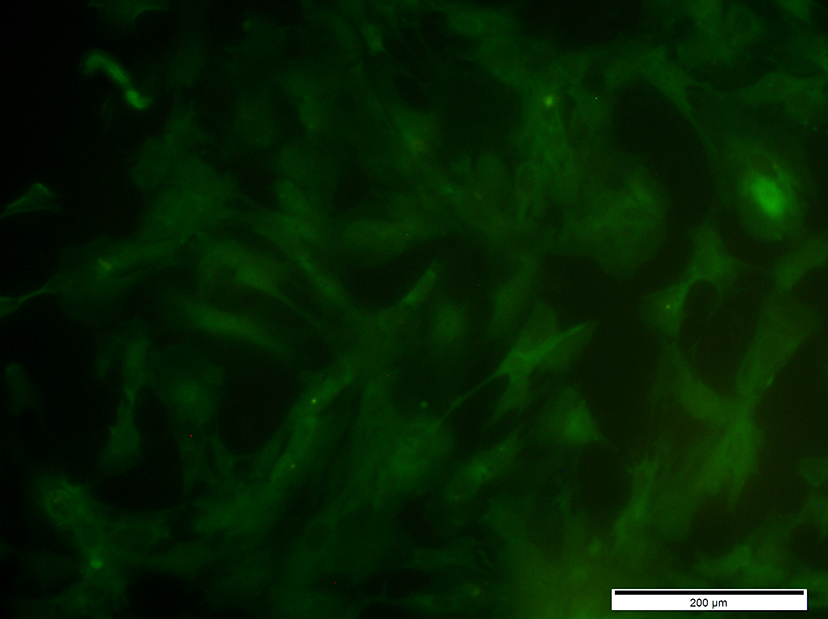

Supplement: Supplementary file 3 [file Data_Sheet_3.ZIP › Immunofluorescence original image/Fig.8 (nuclear translocation of NF-a╩Bp65)/con group/NF-a╩Bp65 (green).jpg]

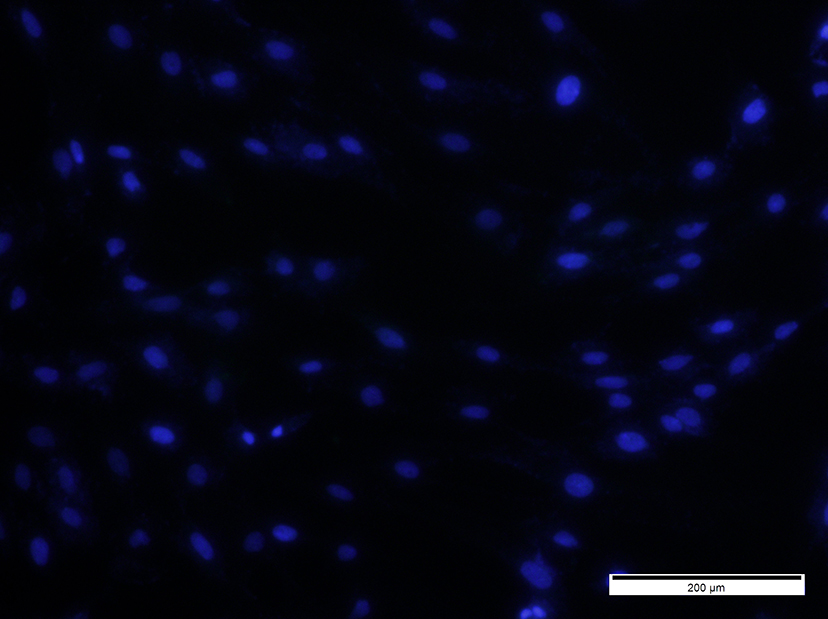

Supplement: Supplementary file 3 [file Data_Sheet_3.ZIP › Immunofluorescence original image/Fig.8 (nuclear translocation of NF-a╩Bp65)/LPS group/DAPI (blue).jpg]

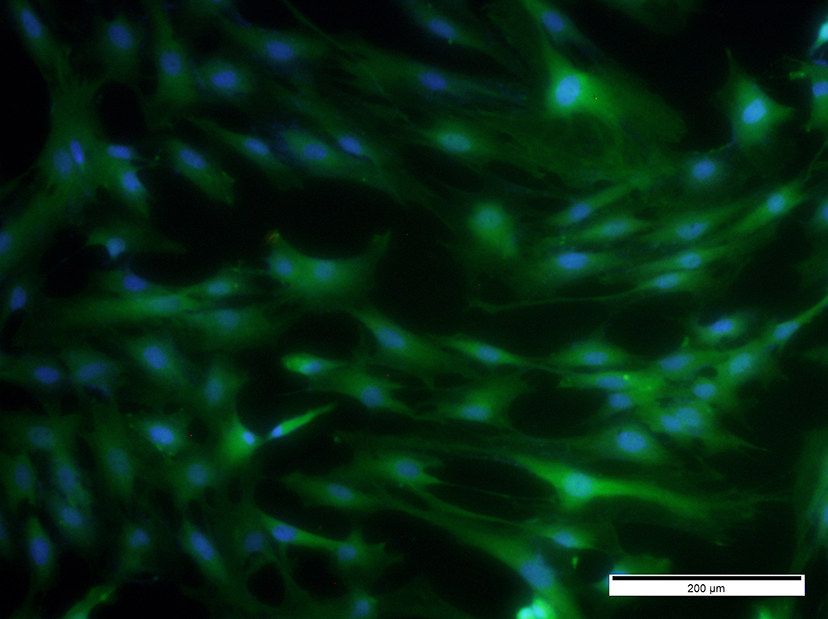

Supplement: Supplementary file 3 [file Data_Sheet_3.ZIP › Immunofluorescence original image/Fig.8 (nuclear translocation of NF-a╩Bp65)/LPS group/merge.jpg]

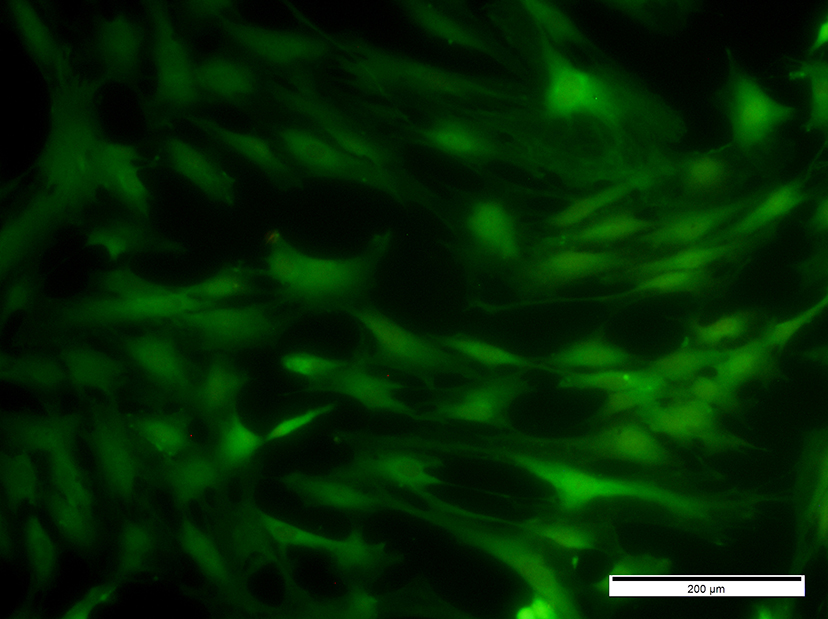

Supplement: Supplementary file 3 [file Data_Sheet_3.ZIP › Immunofluorescence original image/Fig.8 (nuclear translocation of NF-a╩Bp65)/LPS group/NF-a╩Bp65 (green).jpg]

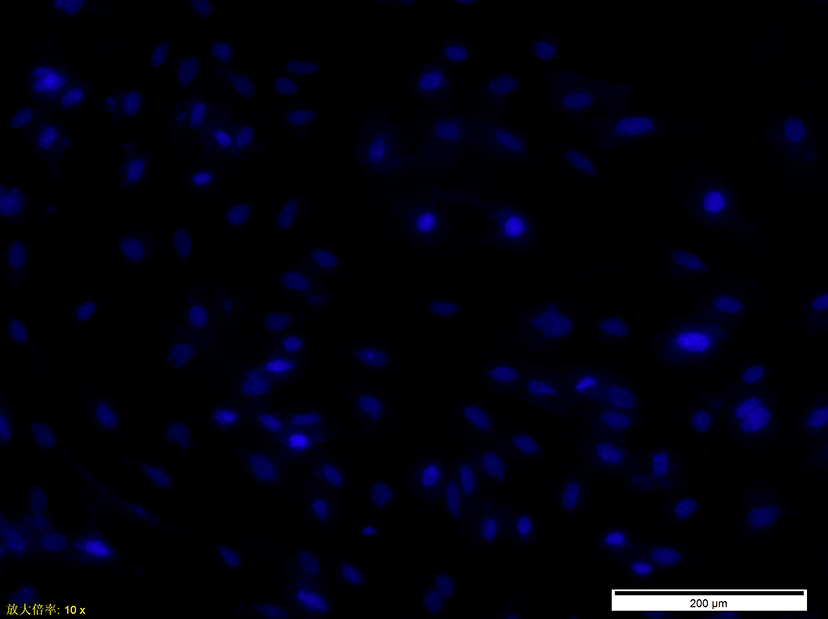

Supplement: Supplementary file 3 [file Data_Sheet_3.ZIP › Immunofluorescence original image/Fig.8 (nuclear translocation of NF-a╩Bp65)/LPS+SR11302 group/DAPI (blue).jpg]

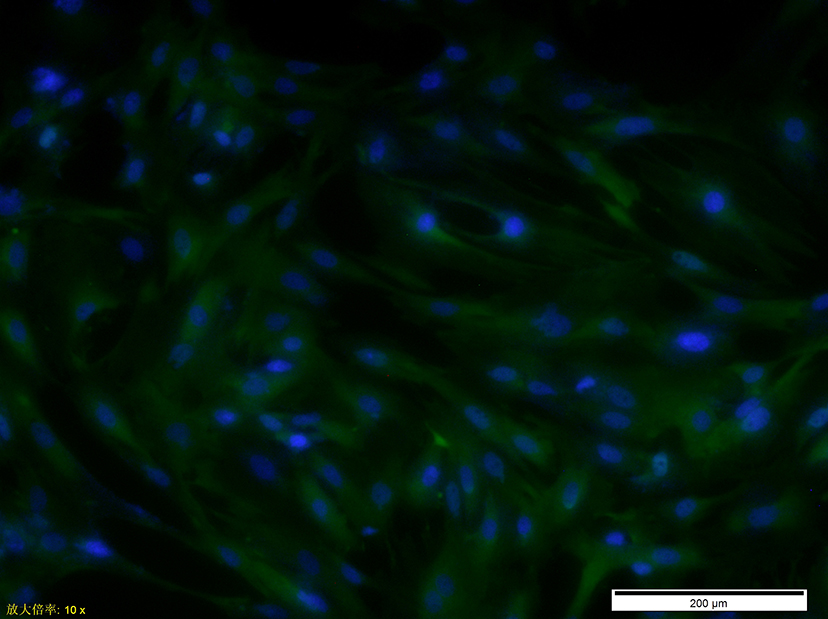

Supplement: Supplementary file 3 [file Data_Sheet_3.ZIP › Immunofluorescence original image/Fig.8 (nuclear translocation of NF-a╩Bp65)/LPS+SR11302 group/merge.jpg]

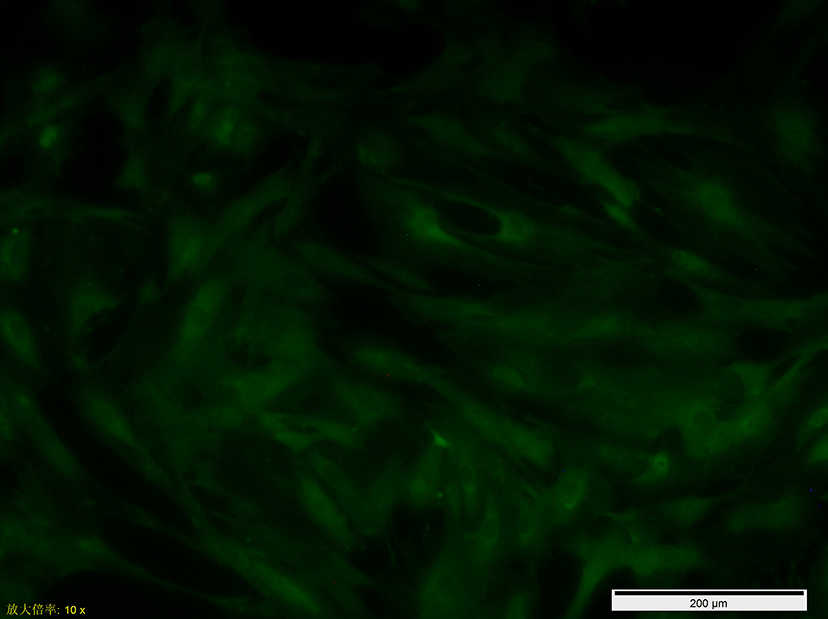

Supplement: Supplementary file 3 [file Data_Sheet_3.ZIP › Immunofluorescence original image/Fig.8 (nuclear translocation of NF-a╩Bp65)/LPS+SR11302 group/NF-a╩Bp65 (green).jpg]

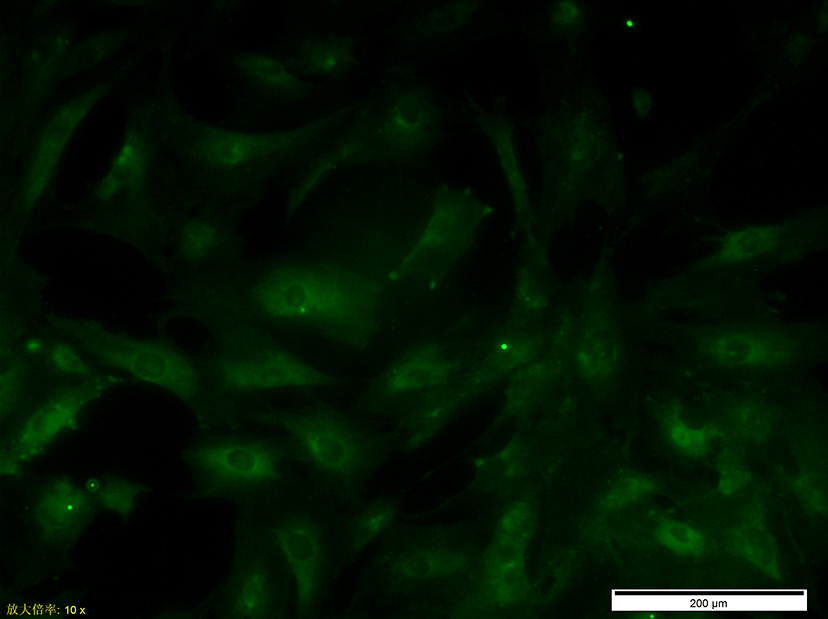

Supplement: Supplementary file 3 [file Data_Sheet_3.ZIP › Immunofluorescence original image/Fig.9 (nuclear translocation of AP-1)/con group/AP-1 (green).jpg]

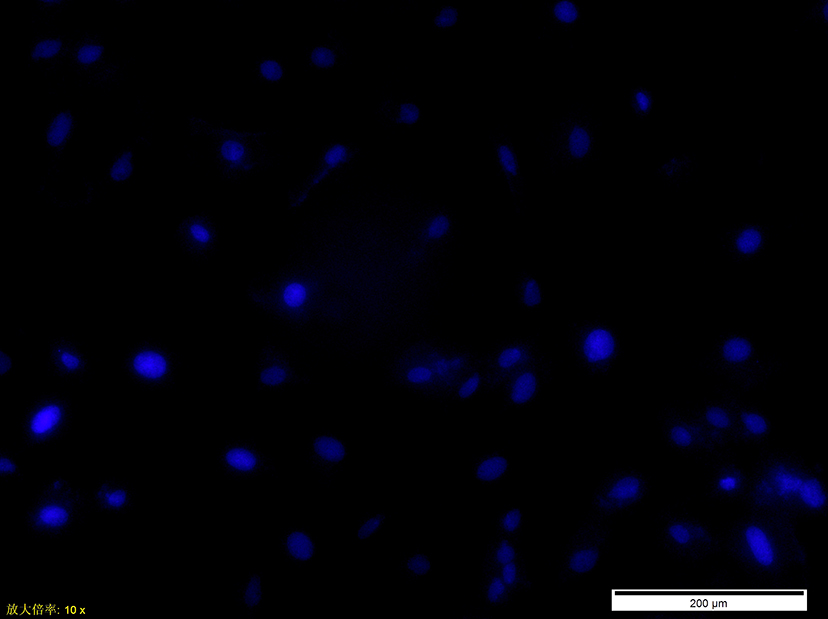

Supplement: Supplementary file 3 [file Data_Sheet_3.ZIP › Immunofluorescence original image/Fig.9 (nuclear translocation of AP-1)/con group/DAPI (blue).jpg]

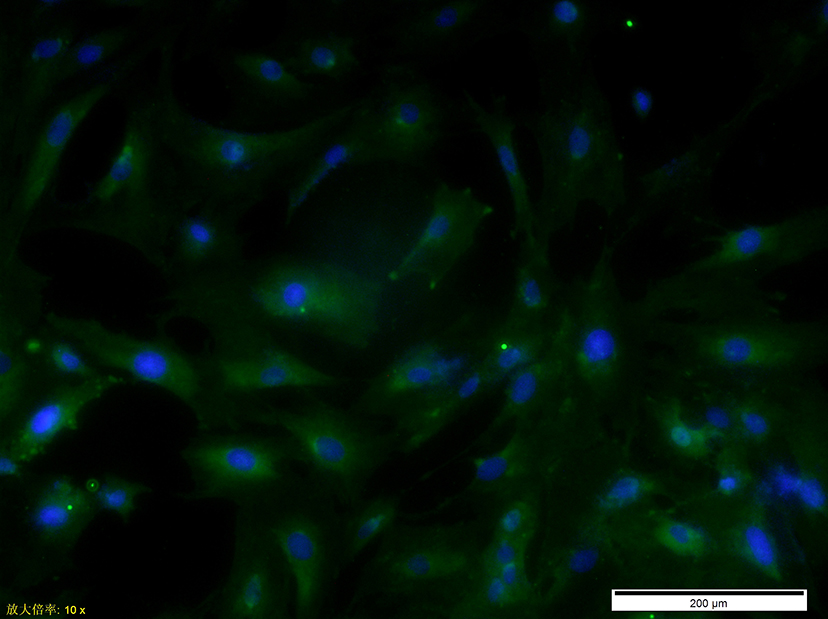

Supplement: Supplementary file 3 [file Data_Sheet_3.ZIP › Immunofluorescence original image/Fig.9 (nuclear translocation of AP-1)/con group/merge.jpg]

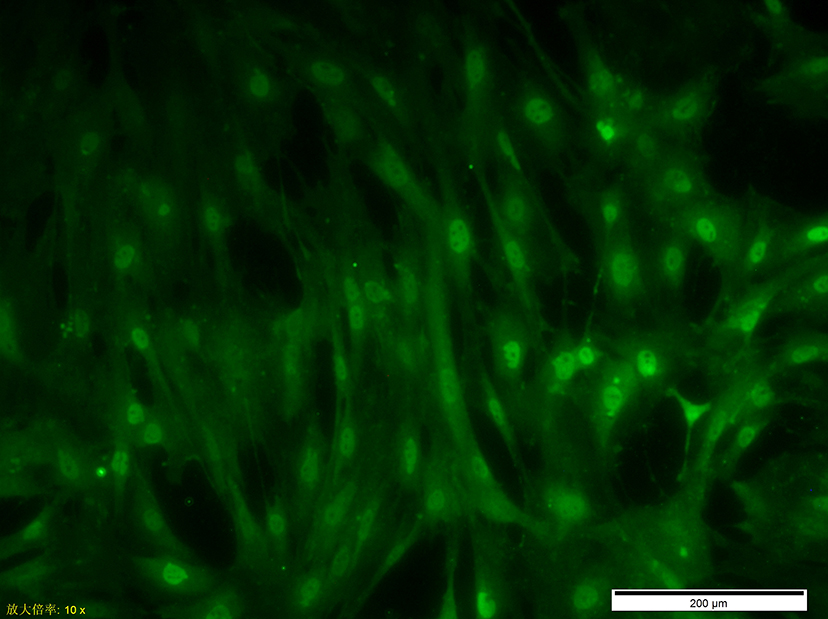

Supplement: Supplementary file 3 [file Data_Sheet_3.ZIP › Immunofluorescence original image/Fig.9 (nuclear translocation of AP-1)/LPS group/AP-1 (green).jpg]

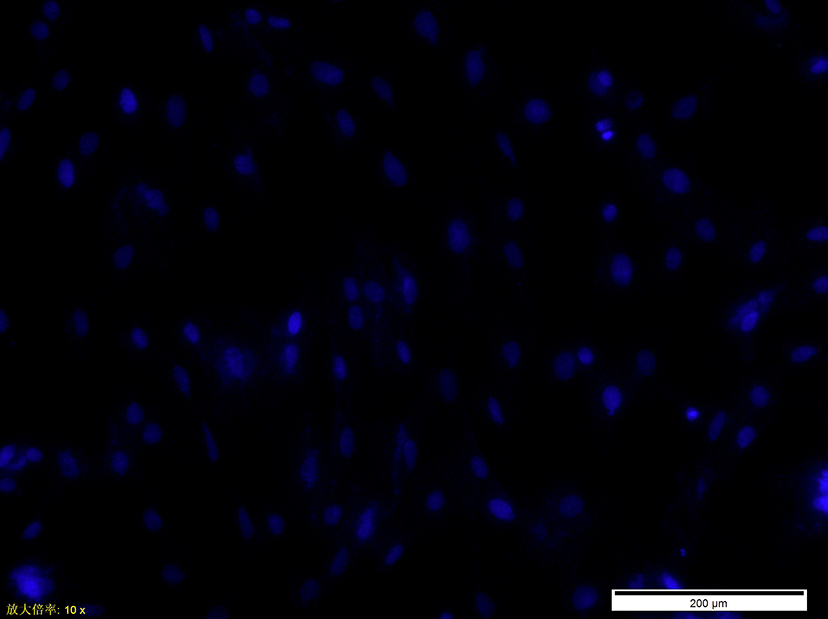

Supplement: Supplementary file 3 [file Data_Sheet_3.ZIP › Immunofluorescence original image/Fig.9 (nuclear translocation of AP-1)/LPS group/DAPI (blue).jpg]

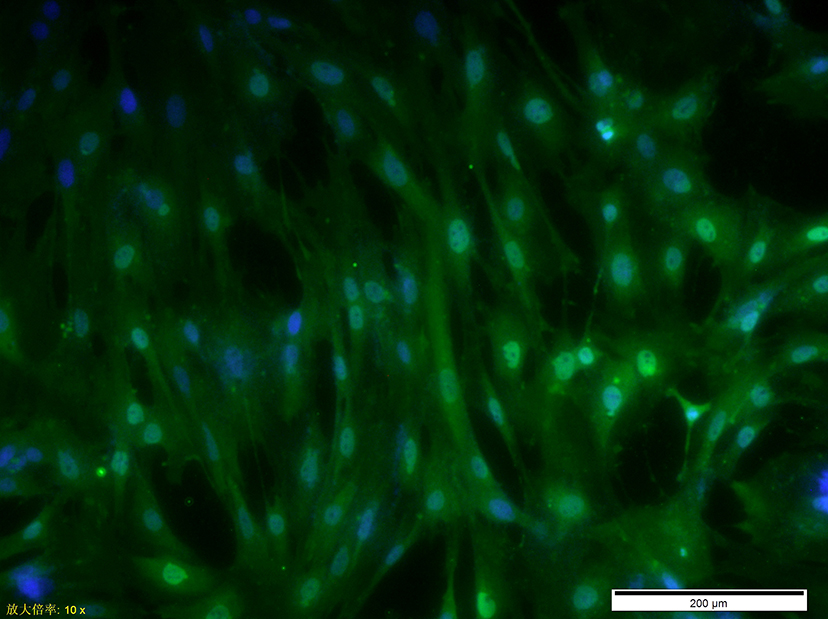

Supplement: Supplementary file 3 [file Data_Sheet_3.ZIP › Immunofluorescence original image/Fig.9 (nuclear translocation of AP-1)/LPS group/merge.jpg]

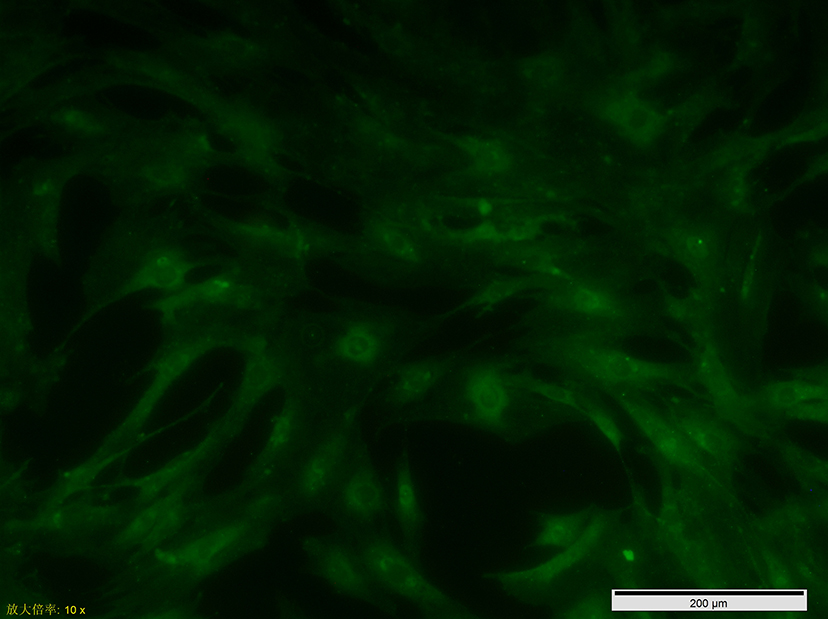

Supplement: Supplementary file 3 [file Data_Sheet_3.ZIP › Immunofluorescence original image/Fig.9 (nuclear translocation of AP-1)/LPS+PDTC group/AP-1 (green).jpg]

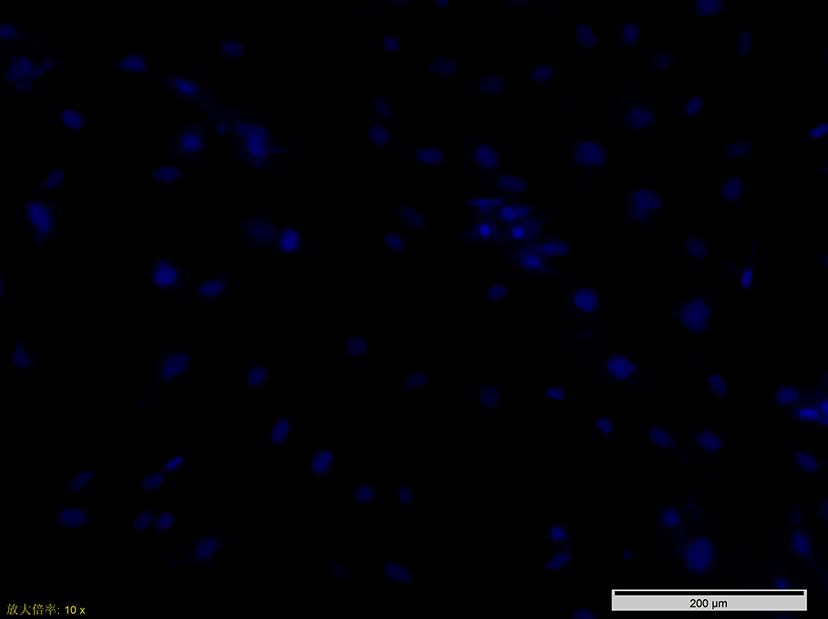

Supplement: Supplementary file 3 [file Data_Sheet_3.ZIP › Immunofluorescence original image/Fig.9 (nuclear translocation of AP-1)/LPS+PDTC group/DAPI (blue).jpg]

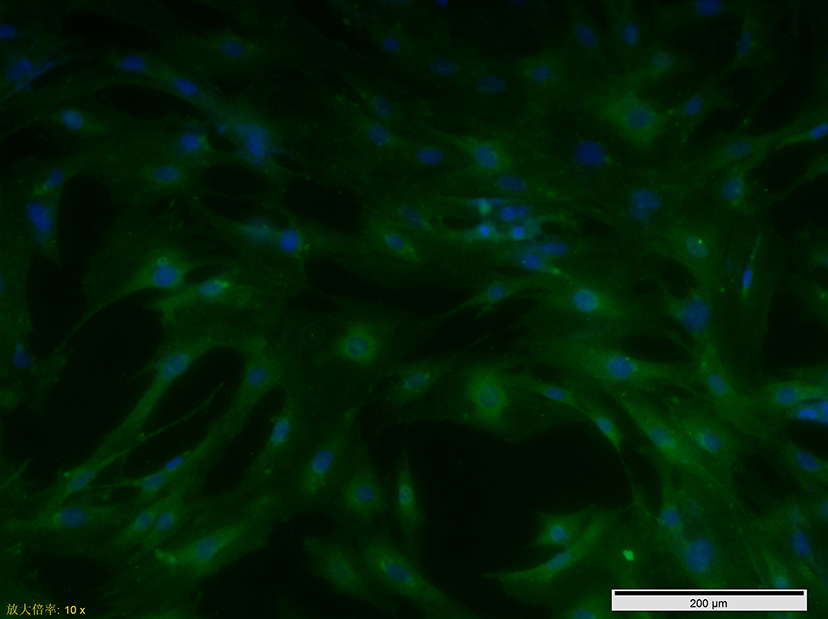

Supplement: Supplementary file 3 [file Data_Sheet_3.ZIP › Immunofluorescence original image/Fig.9 (nuclear translocation of AP-1)/LPS+PDTC group/merge.jpg]

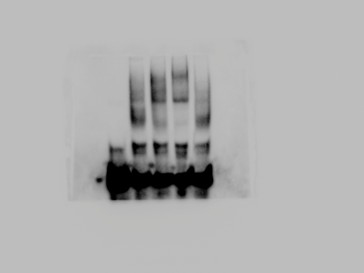

Supplement: Supplementary file 4 [file Data_Sheet_4.ZIP › EMSA original image/Fig. 7/Fig.7A.jpg]

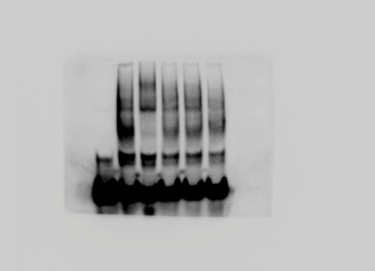

Supplement: Supplementary file 4 [file Data_Sheet_4.ZIP › EMSA original image/Fig. 7/Fig.7B.jpg]

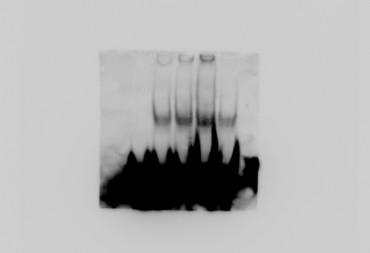

Supplement: Supplementary file 4 [file Data_Sheet_4.ZIP › EMSA original image/Fig. 7/Fig.7C.jpg]

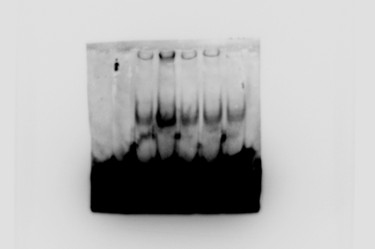

Supplement: Supplementary file 4 [file Data_Sheet_4.ZIP › EMSA original image/Fig. 7/Fig.7D.jpg]

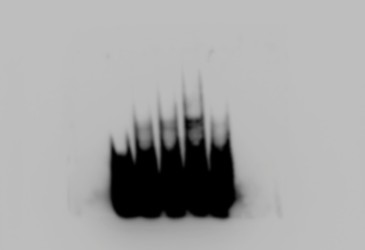

Supplement: Supplementary file 4 [file Data_Sheet_4.ZIP › EMSA original image/Fig. 7/Fig.7E.jpg]

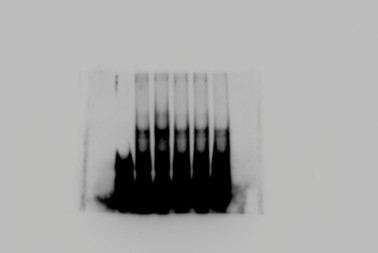

Supplement: Supplementary file 4 [file Data_Sheet_4.ZIP › EMSA original image/Fig. 7/Fig.7F.jpg]

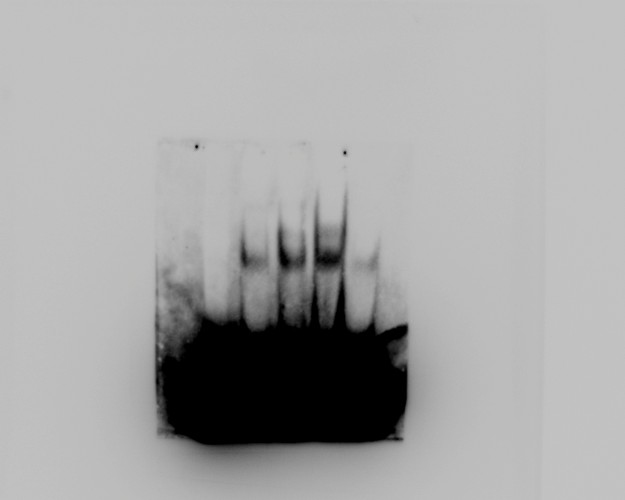

Supplement: Supplementary file 4 [file Data_Sheet_4.ZIP › EMSA original image/Fig. 10/Fig.10H.jpg]

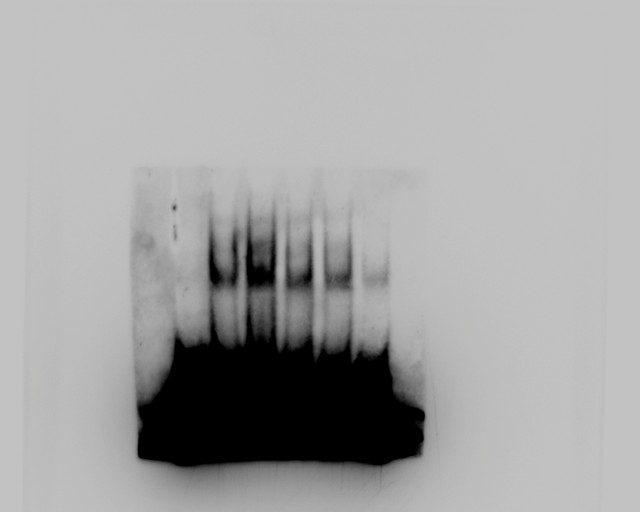

Supplement: Supplementary file 4 [file Data_Sheet_4.ZIP › EMSA original image/Fig. 10/Fig.10I.jpg]

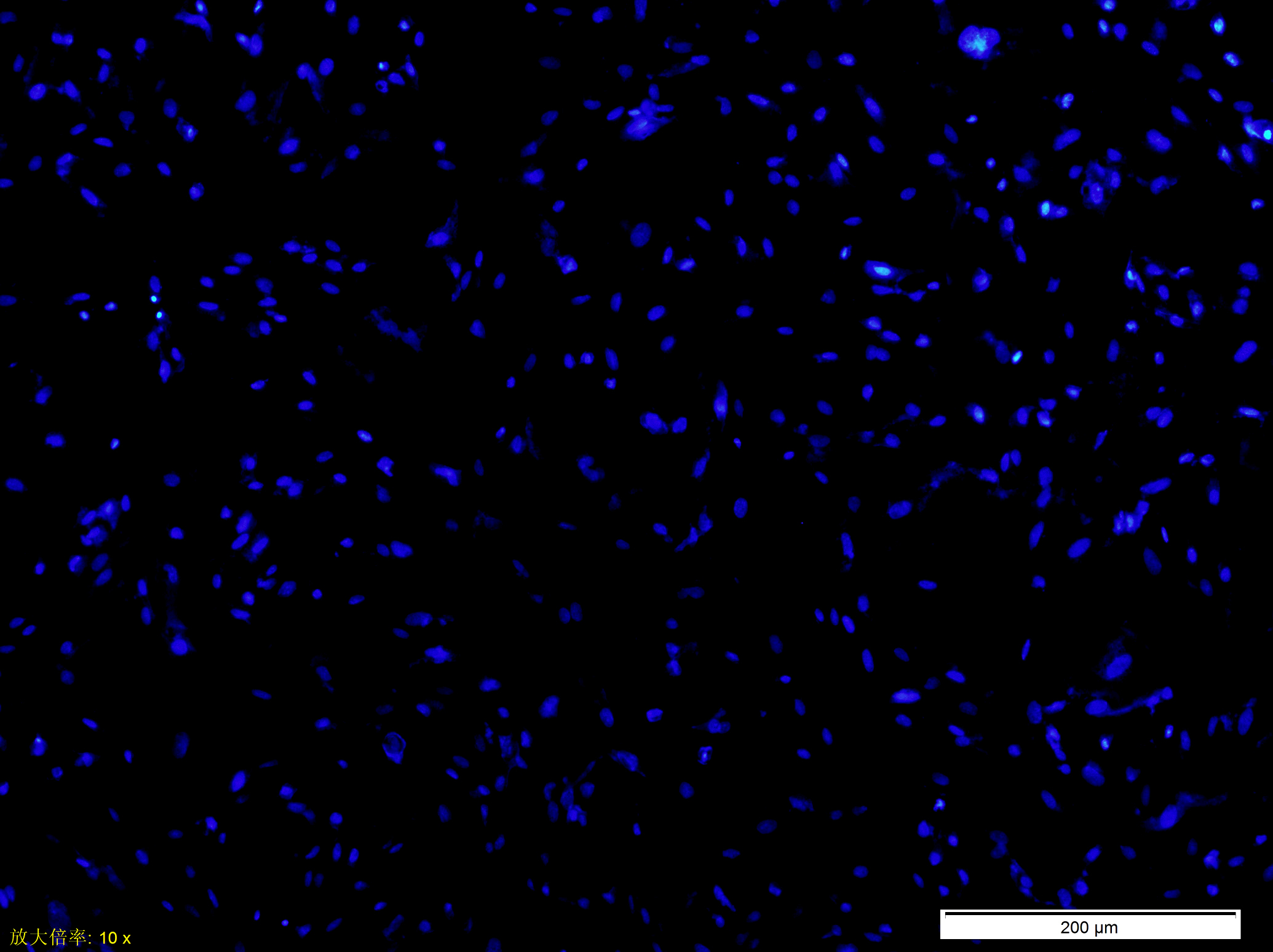

Supplement: Supplementary file 5 [file Data_Sheet_5.ZIP › Fig.11 EtBr uptake original image/con group/DAPI (blue).jpg]

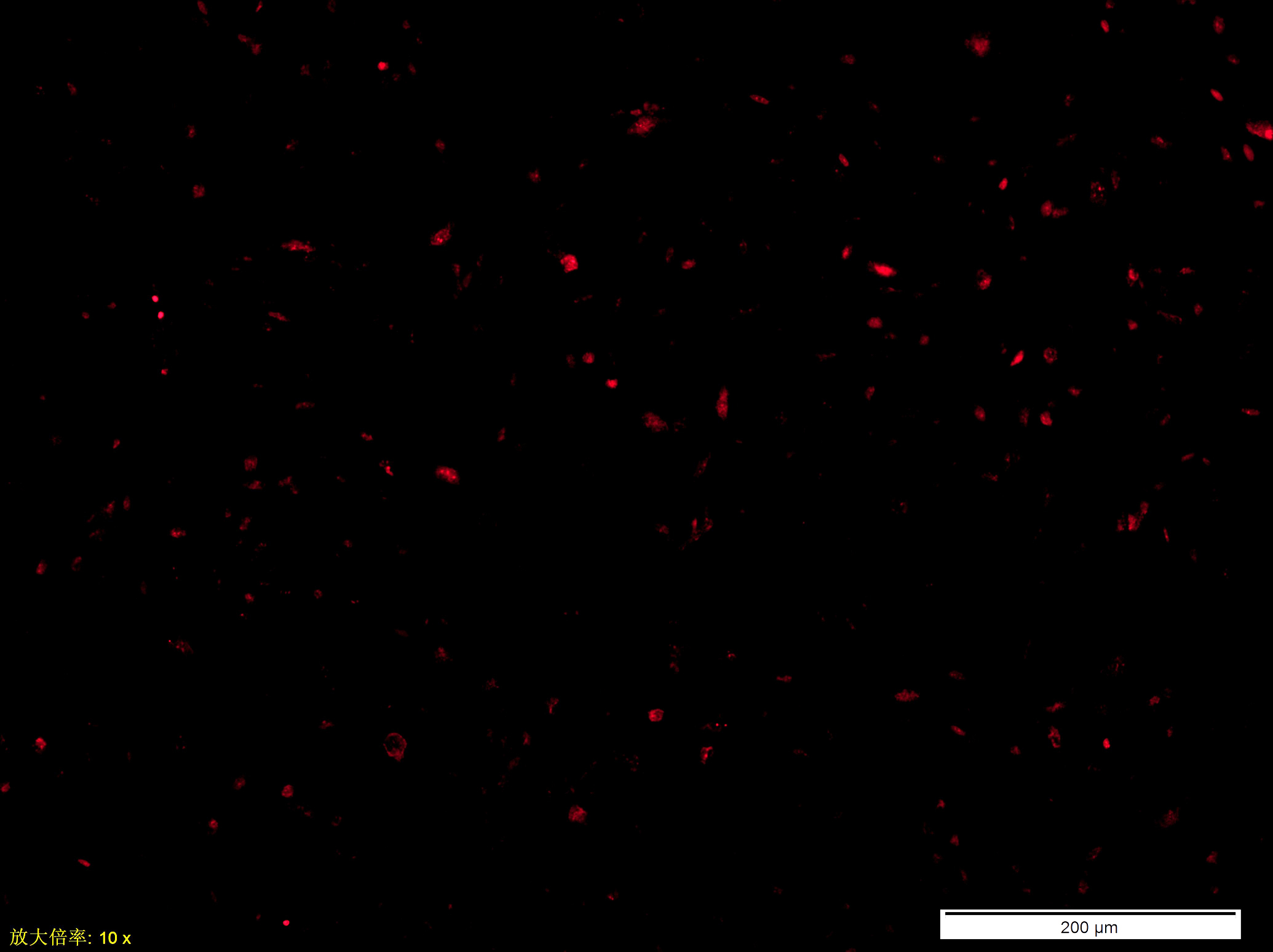

Supplement: Supplementary file 5 [file Data_Sheet_5.ZIP › Fig.11 EtBr uptake original image/con group/Etbr (Red).jpg]

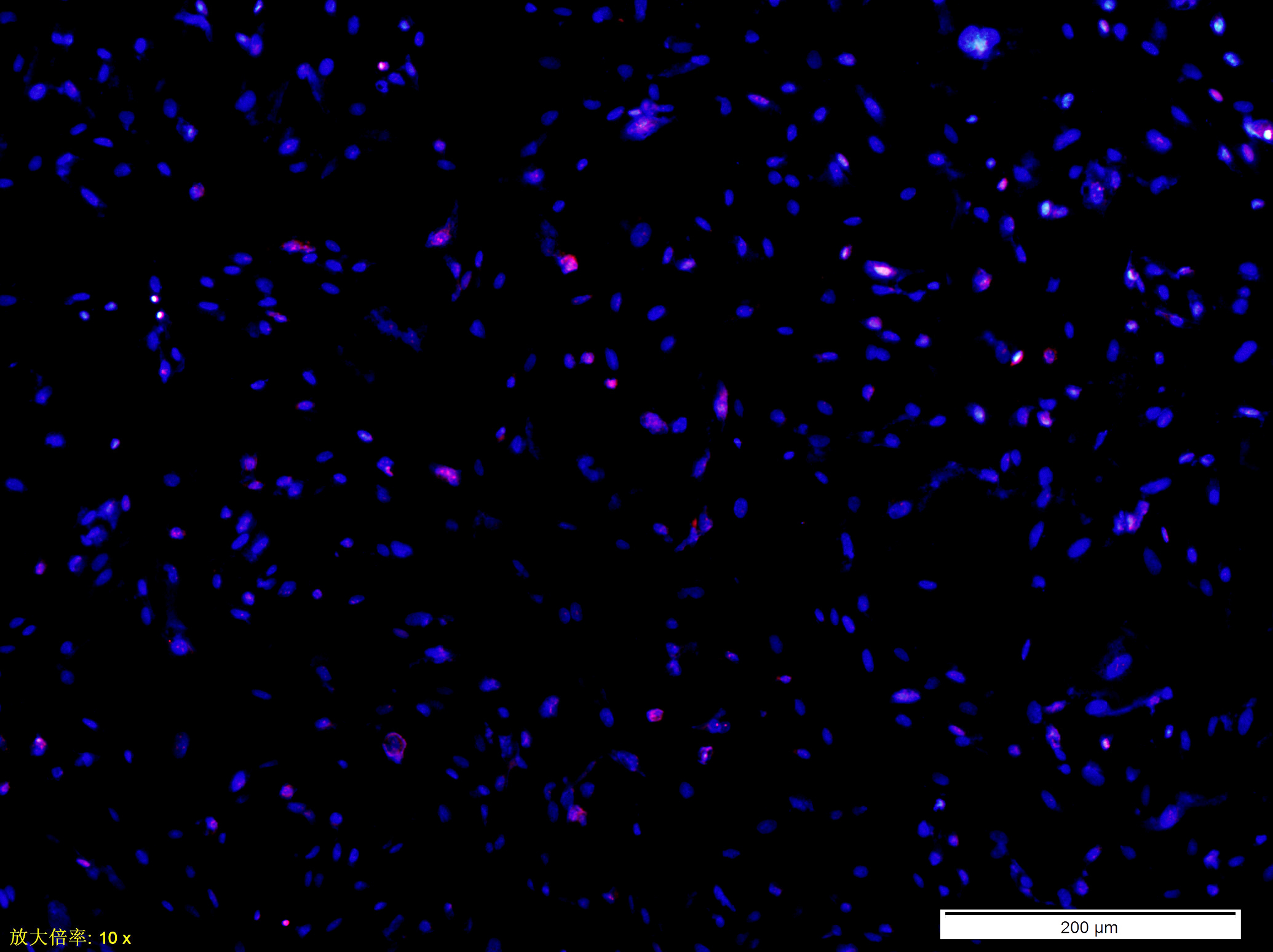

Supplement: Supplementary file 5 [file Data_Sheet_5.ZIP › Fig.11 EtBr uptake original image/con group/merge.jpg]

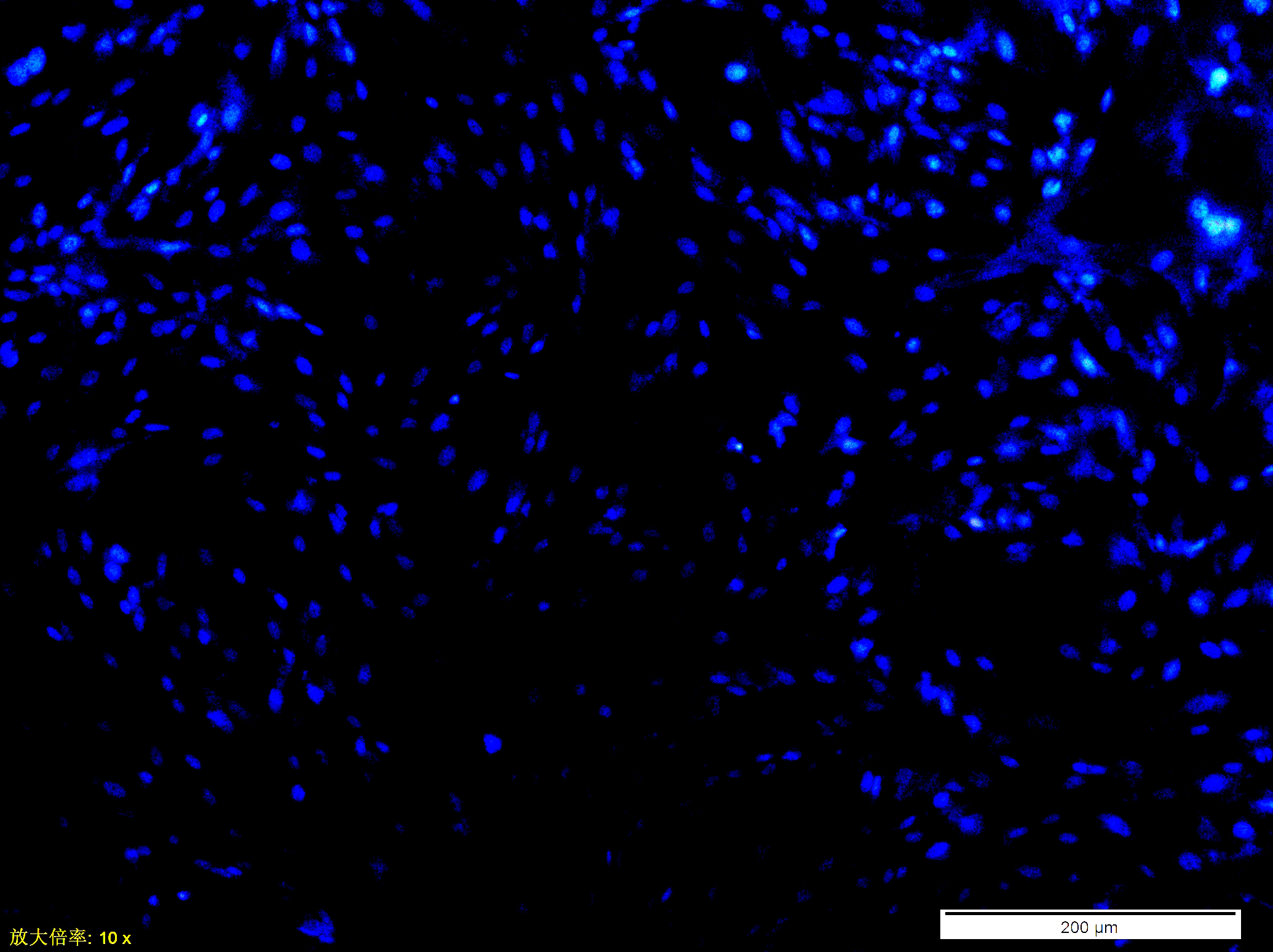

Supplement: Supplementary file 5 [file Data_Sheet_5.ZIP › Fig.11 EtBr uptake original image/LPS group/DAPI (blue).jpg]

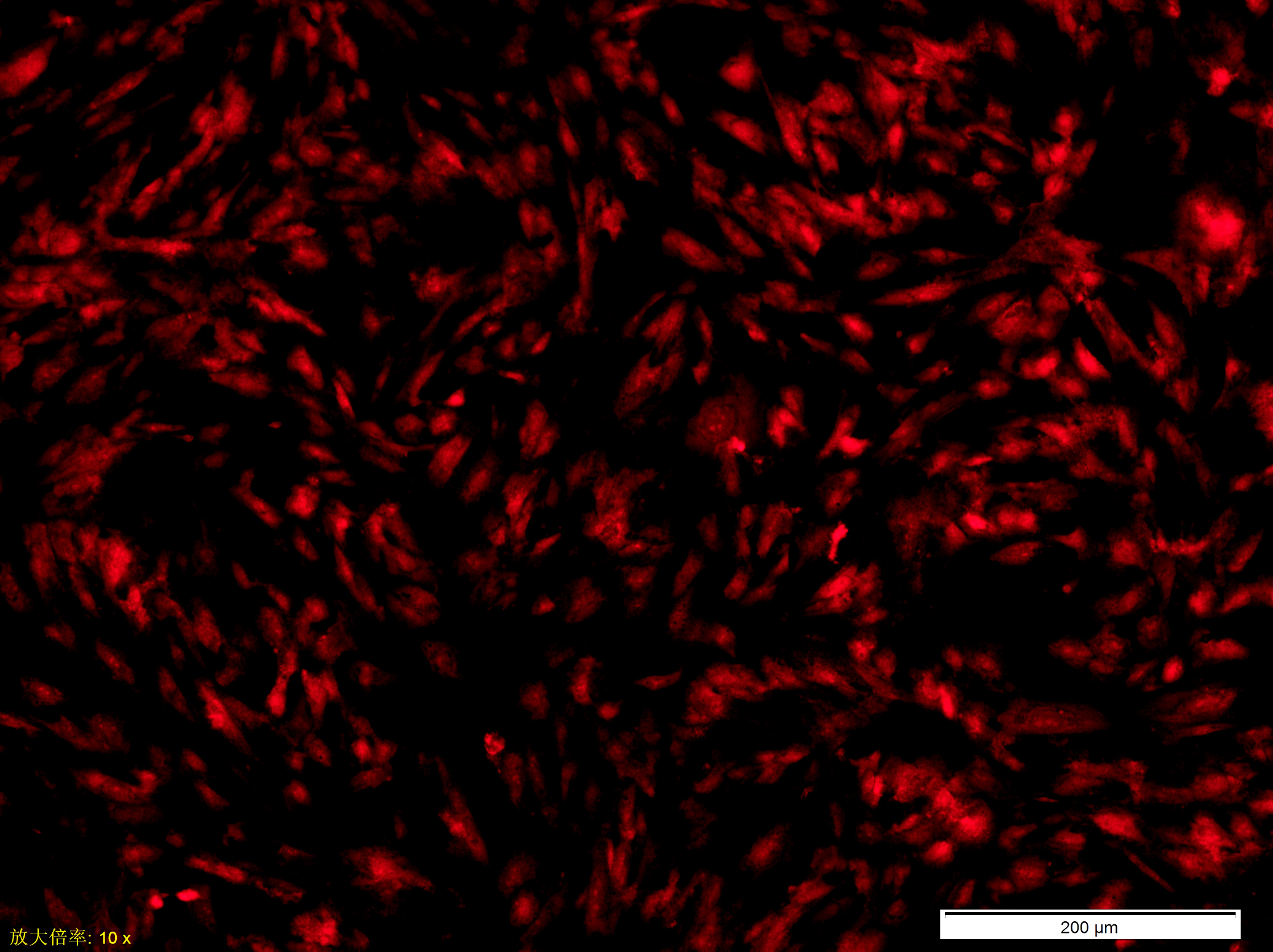

Supplement: Supplementary file 5 [file Data_Sheet_5.ZIP › Fig.11 EtBr uptake original image/LPS group/Etbr (Red).jpg]

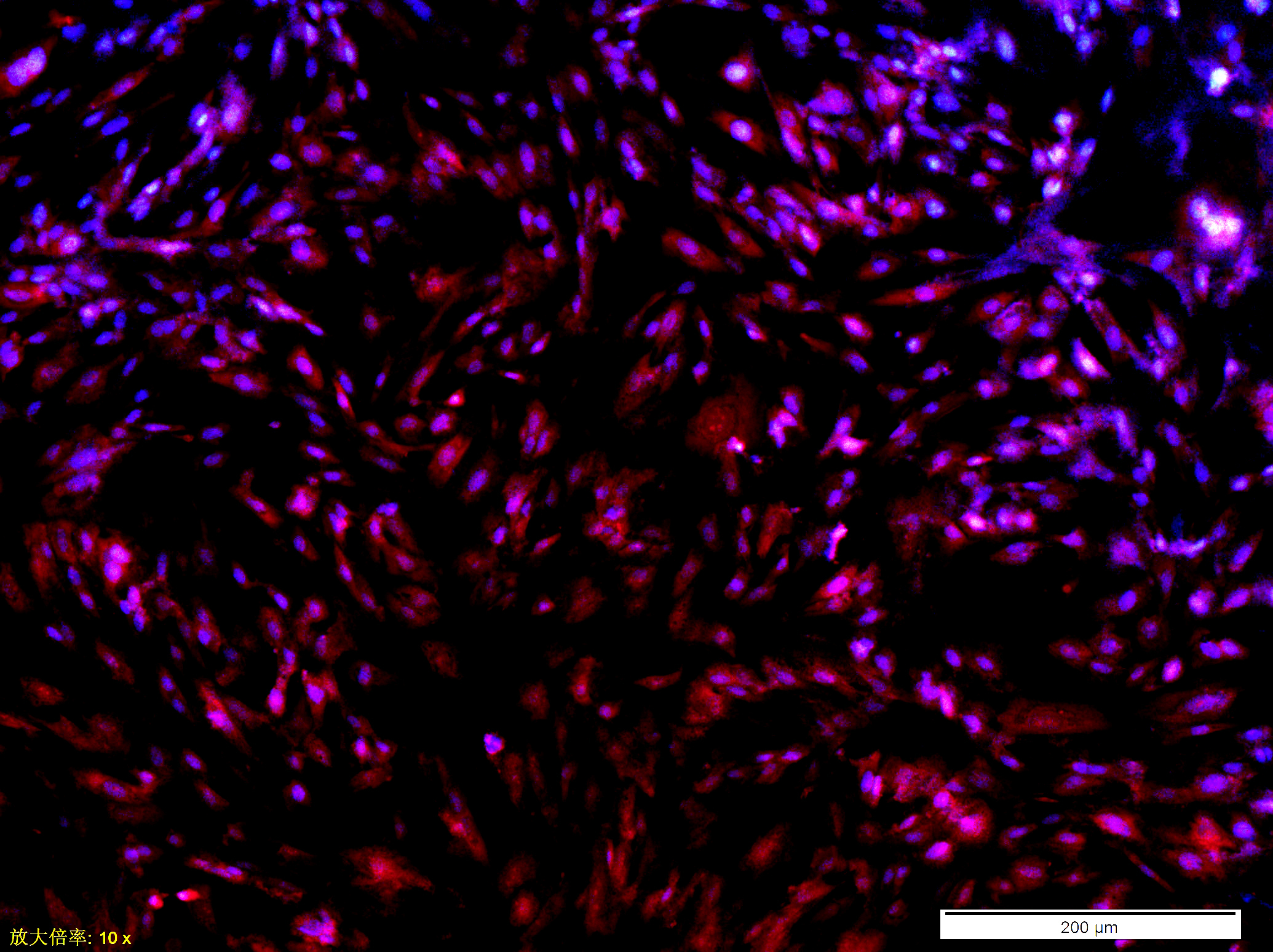

Supplement: Supplementary file 5 [file Data_Sheet_5.ZIP › Fig.11 EtBr uptake original image/LPS group/merge.jpg]

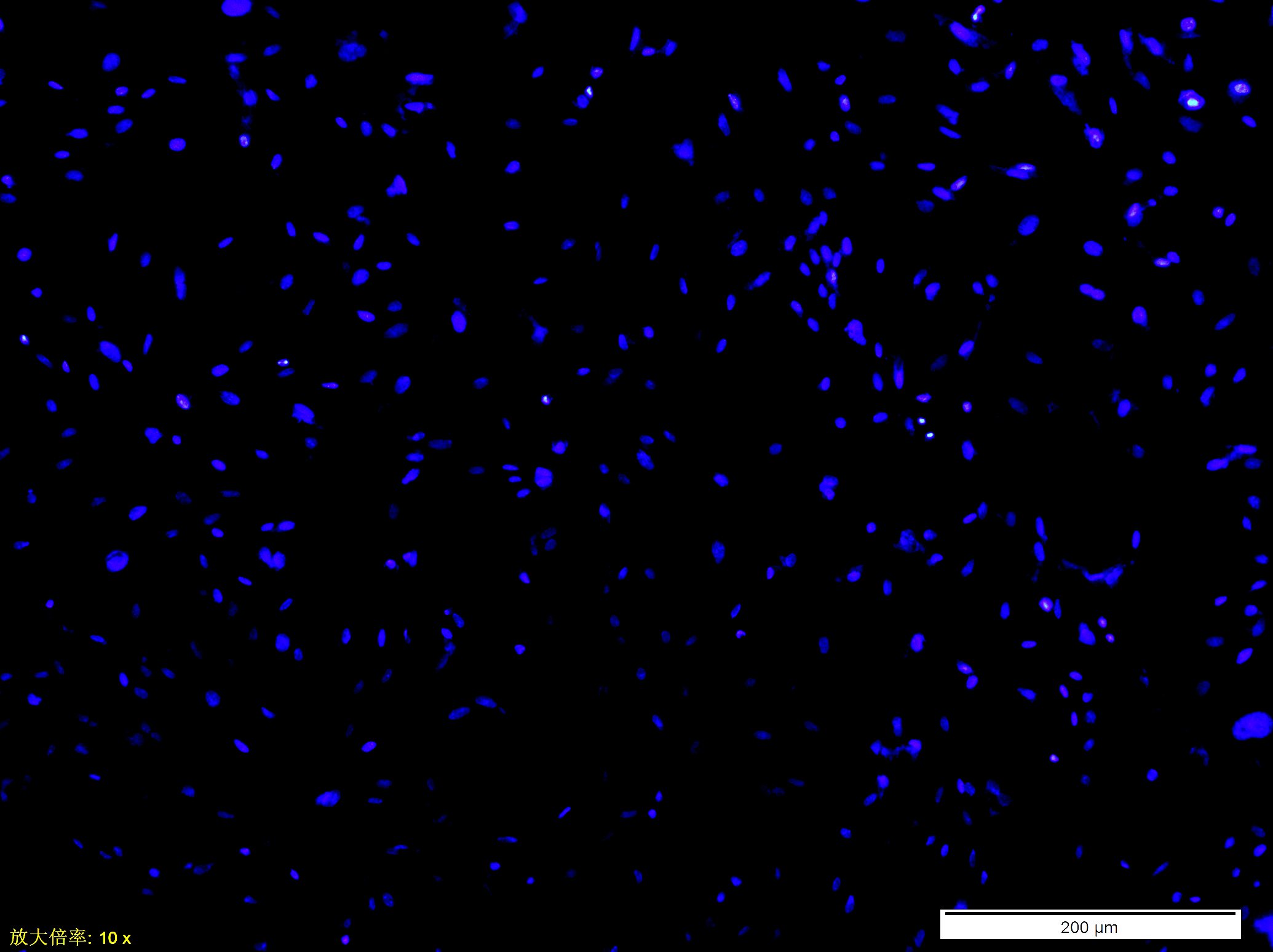

Supplement: Supplementary file 5 [file Data_Sheet_5.ZIP › Fig.11 EtBr uptake original image/LPS+CBX group/DAPI (blue).jpg]

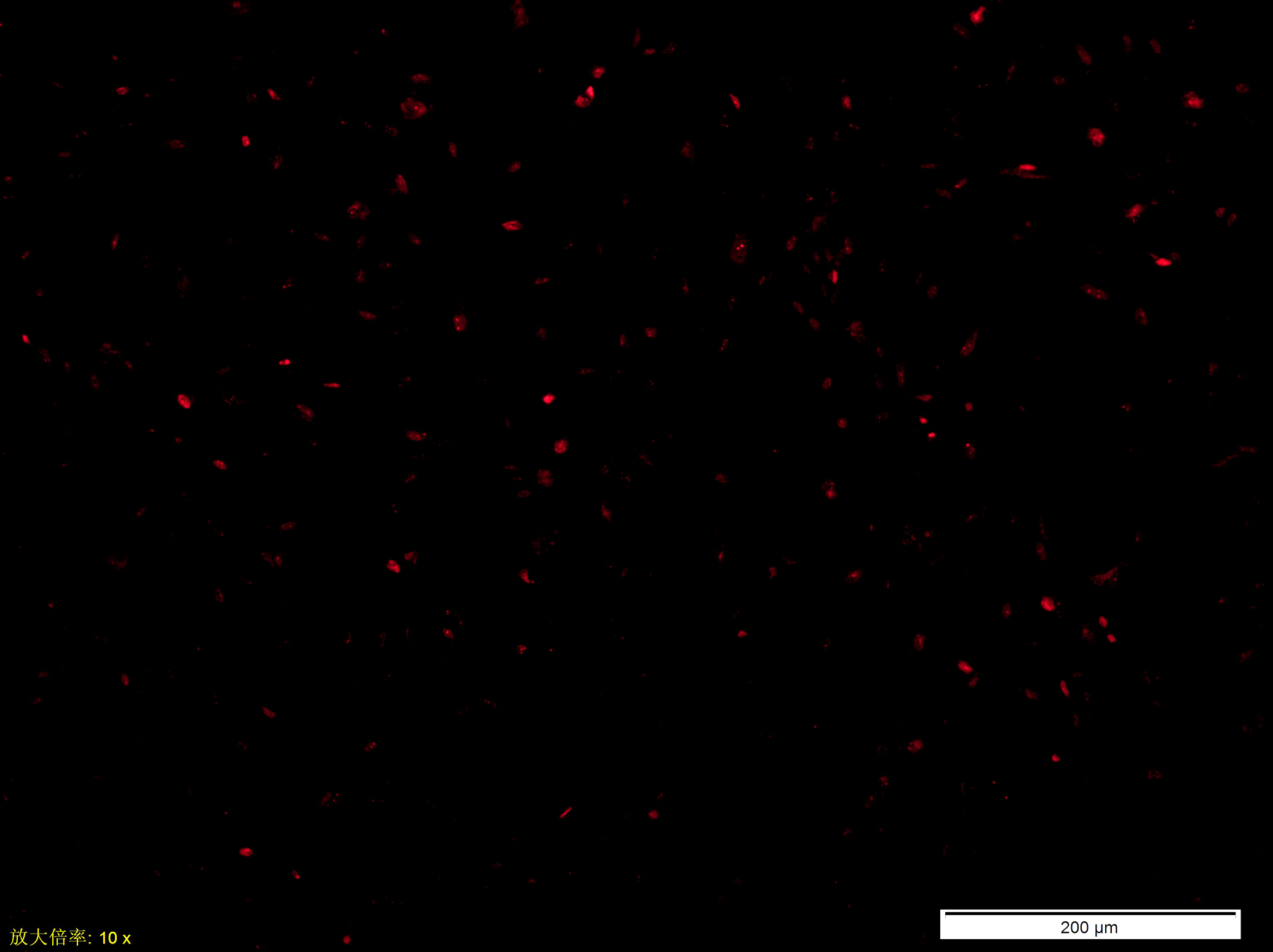

Supplement: Supplementary file 5 [file Data_Sheet_5.ZIP › Fig.11 EtBr uptake original image/LPS+CBX group/Etbr (Red).jpg]

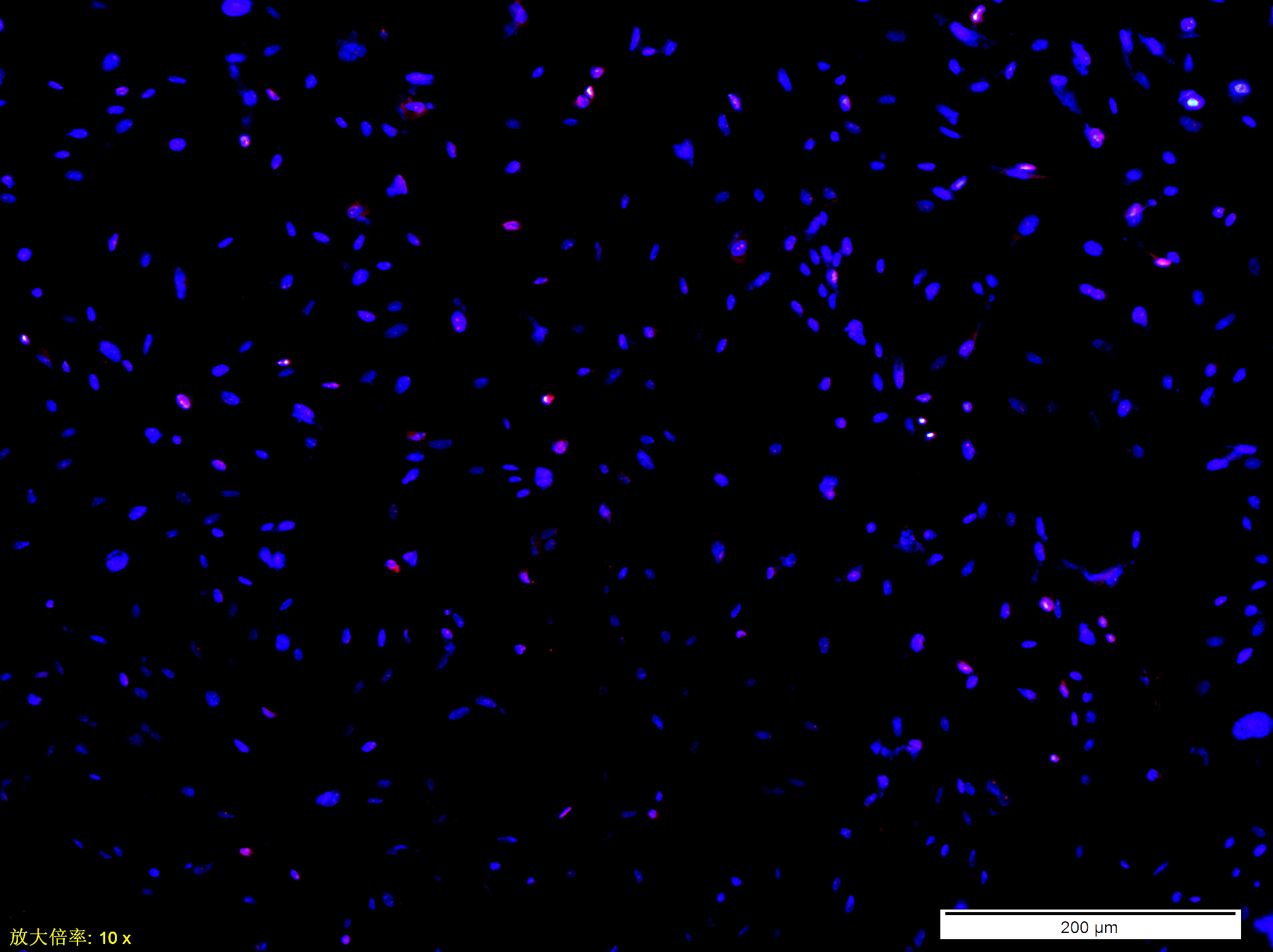

Supplement: Supplementary file 5 [file Data_Sheet_5.ZIP › Fig.11 EtBr uptake original image/LPS+CBX group/merge.jpg]

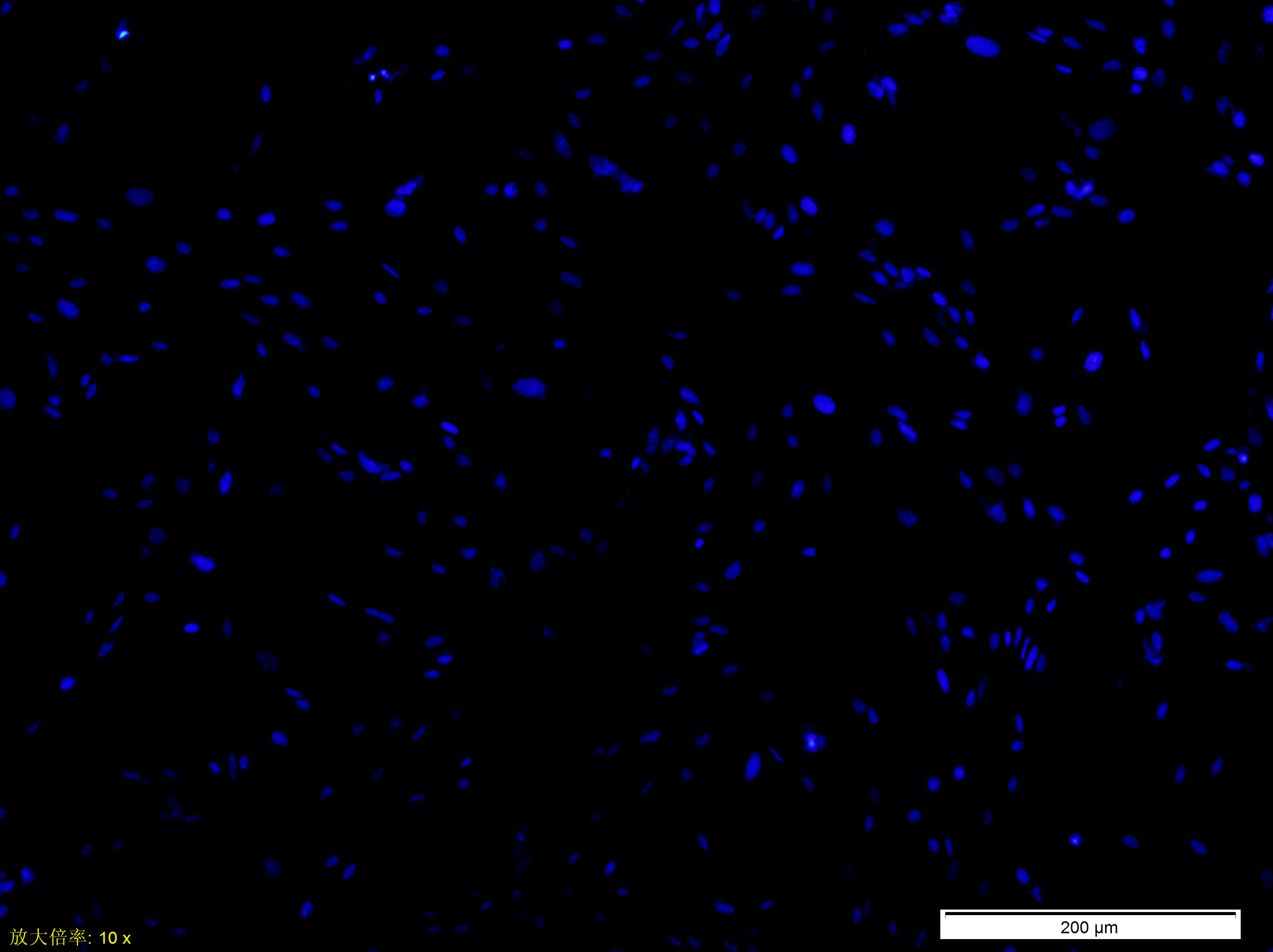

Supplement: Supplementary file 5 [file Data_Sheet_5.ZIP › Fig.11 EtBr uptake original image/LPS+GAP19 group/DAPI (blue).jpg]

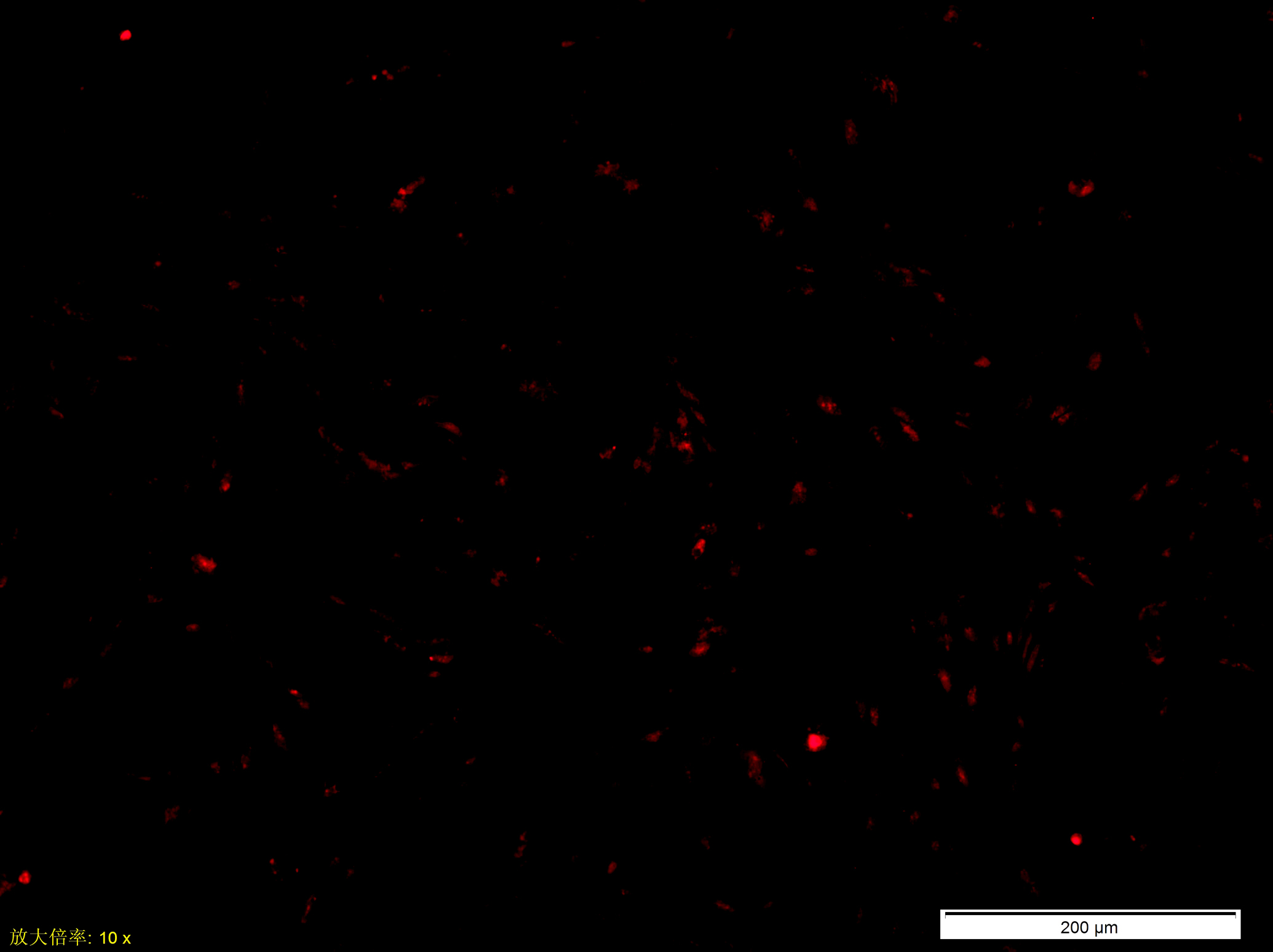

Supplement: Supplementary file 5 [file Data_Sheet_5.ZIP › Fig.11 EtBr uptake original image/LPS+GAP19 group/Etbr (Red).jpg]

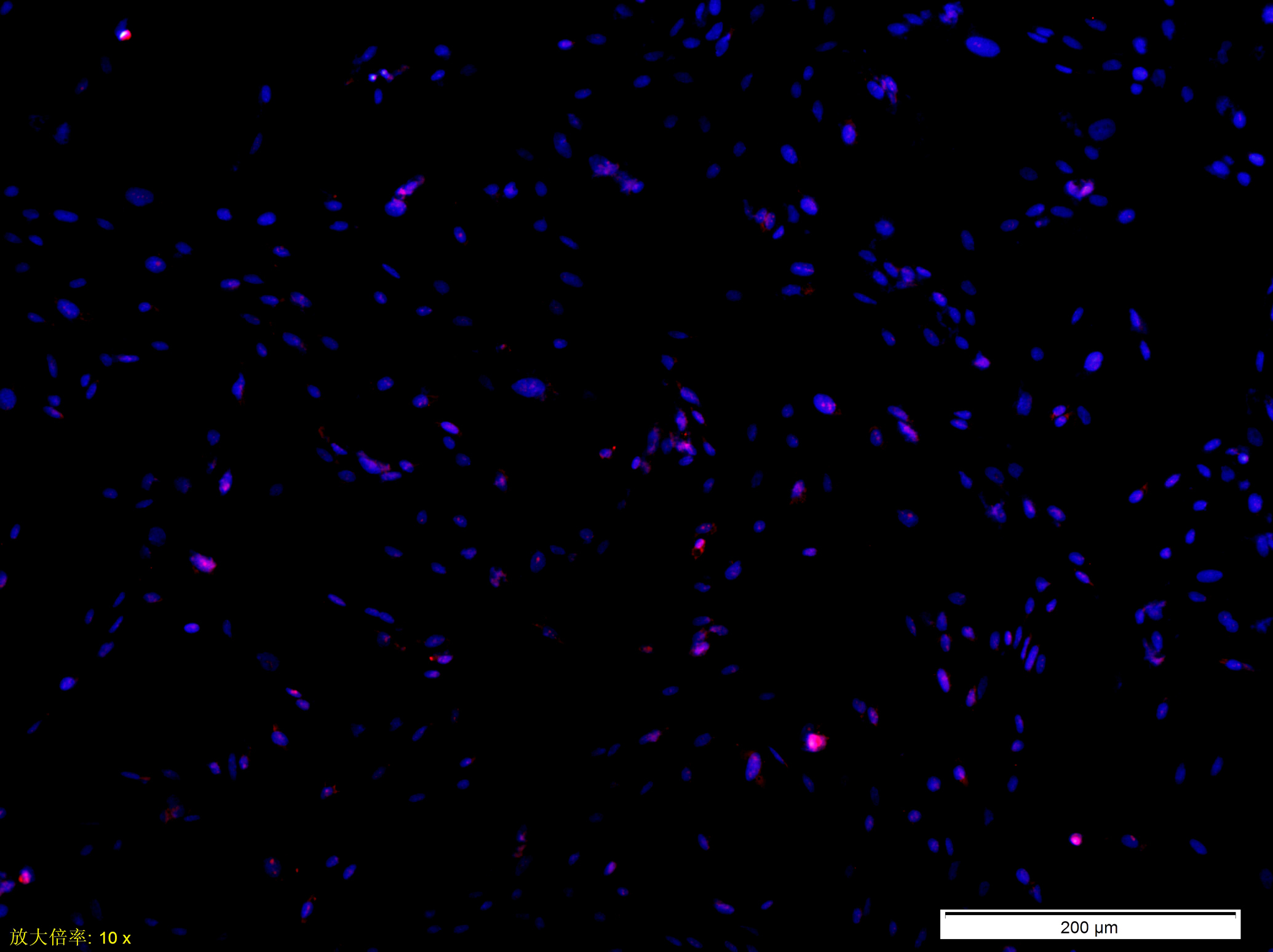

Supplement: Supplementary file 5 [file Data_Sheet_5.ZIP › Fig.11 EtBr uptake original image/LPS+GAP19 group/merge.jpg]

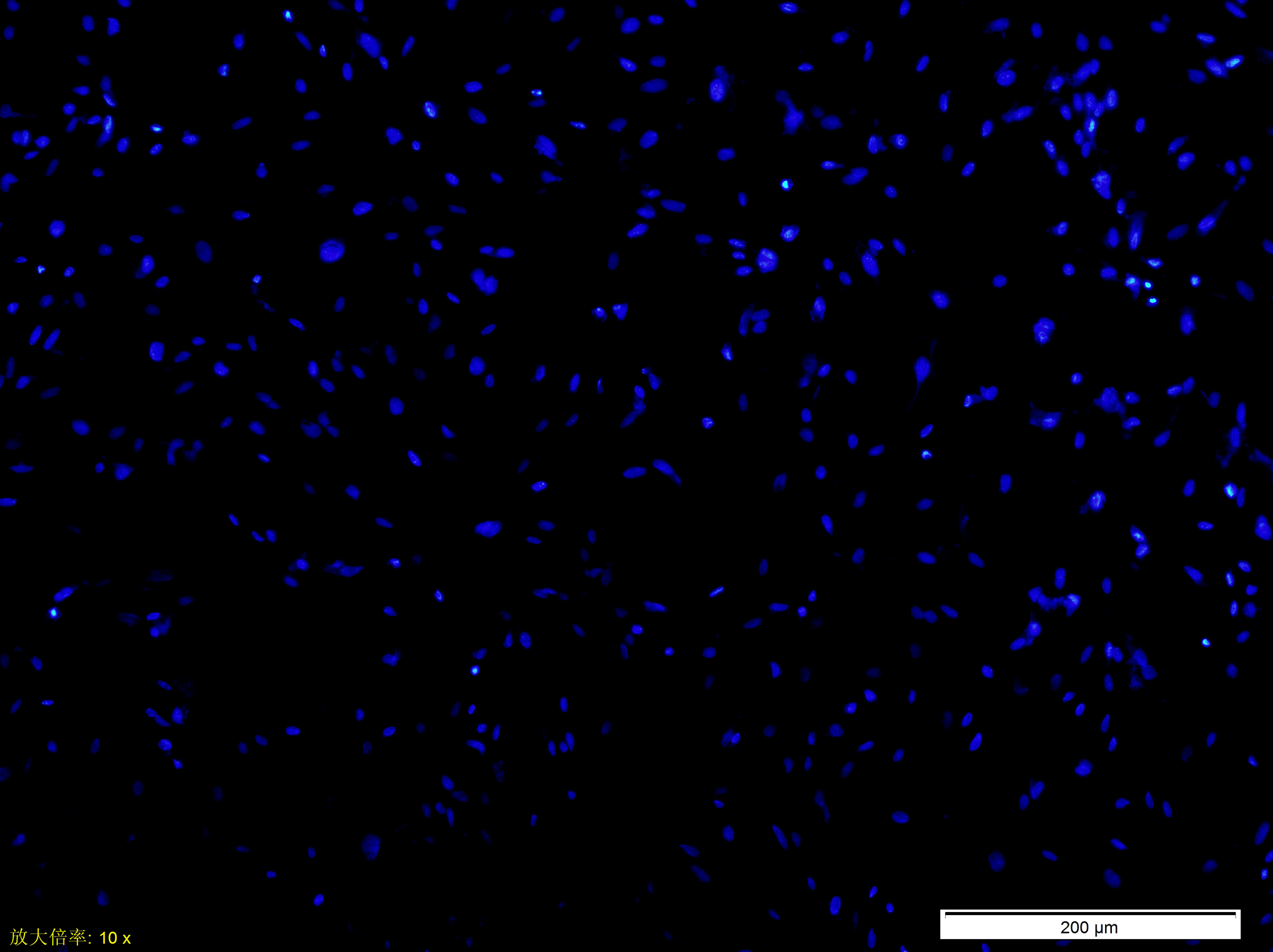

Supplement: Supplementary file 5 [file Data_Sheet_5.ZIP › Fig.11 EtBr uptake original image/LPS+GAP26 group/DAPI (blue).jpg]
