# Supplementary figures and images for: NF-κB and AP-1 are required for the lipopolysaccharide-induced expression of MCP-1, CXCL1, and Cx43 in cultured rat dorsal spinal cord astrocytes (part 2 of 2)
Source: Front Mol Neurosci. 2022 Jul 28;15:859558. doi: 10.3389/fnmol.2022.859558 (PMC9368326; doi:10.3389/fnmol.2022.859558)

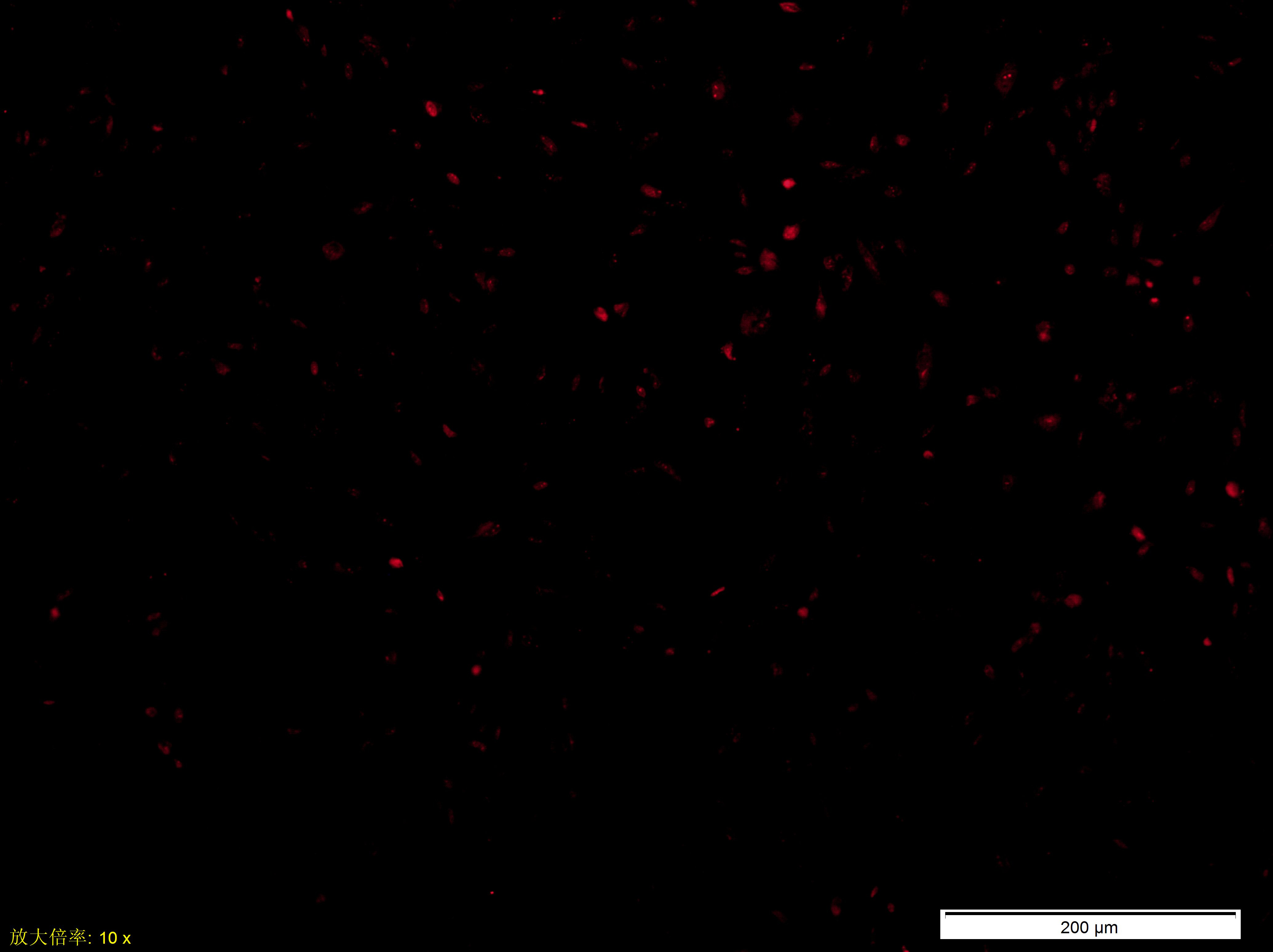

Supplement: Supplementary file 5 [file Data_Sheet_5.ZIP › Fig.11 EtBr uptake original image/LPS+GAP26 group/Etbr (Red).jpg]

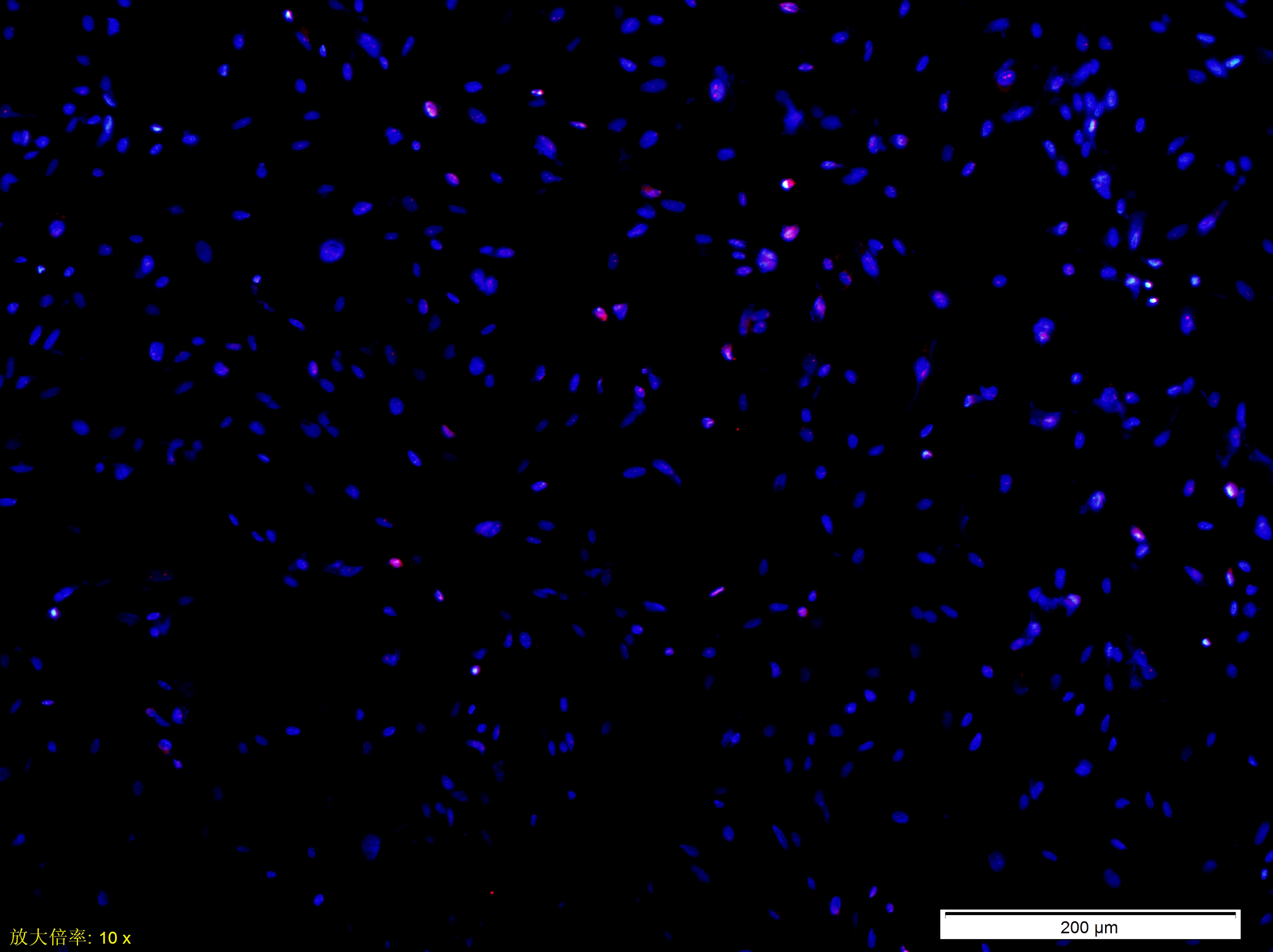

Supplement: Supplementary file 5 [file Data_Sheet_5.ZIP › Fig.11 EtBr uptake original image/LPS+GAP26 group/merge.jpg]

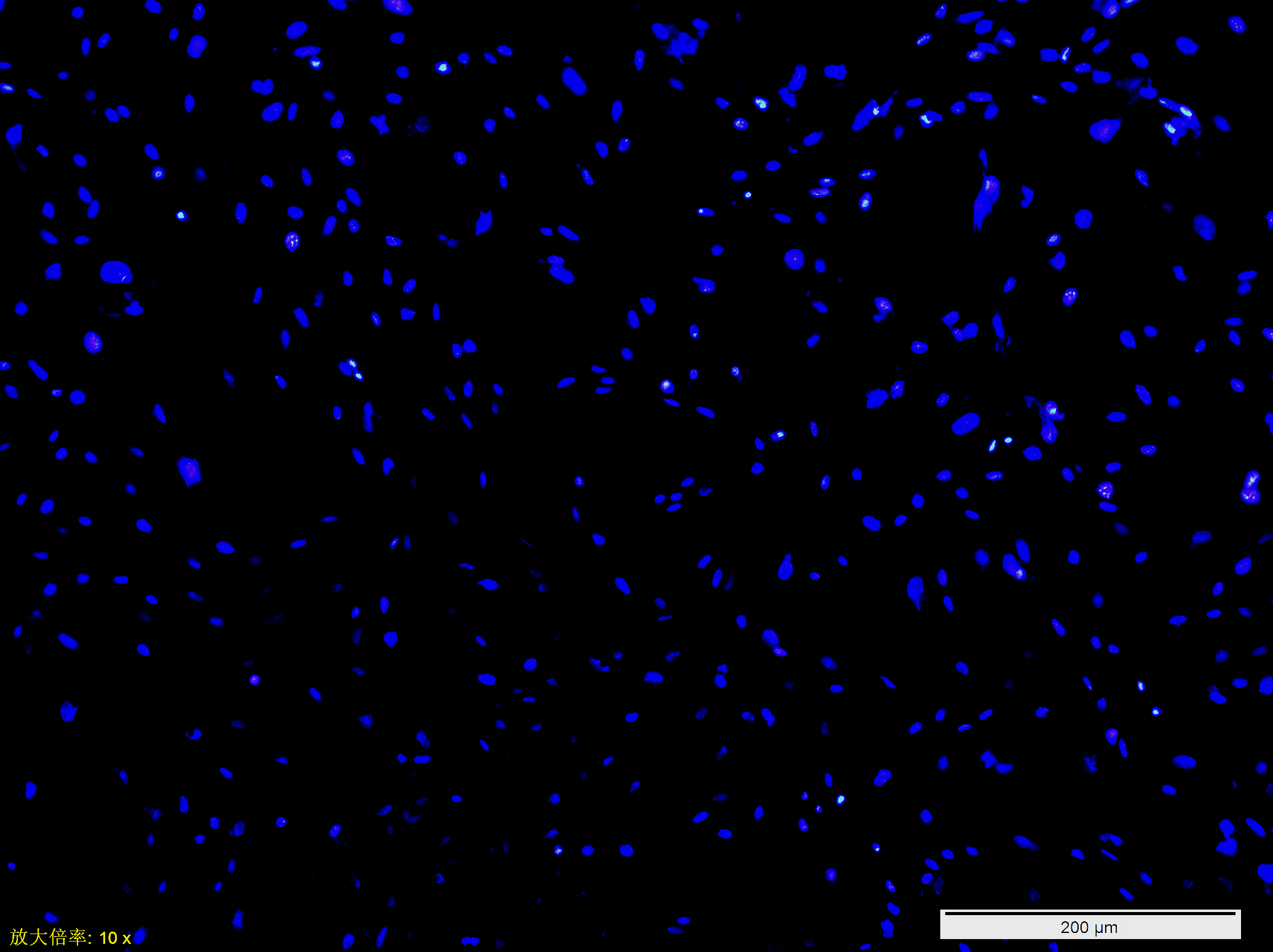

Supplement: Supplementary file 5 [file Data_Sheet_5.ZIP › Fig.11 EtBr uptake original image/LPS+TAK-242 group/DAPI (blue).jpg]

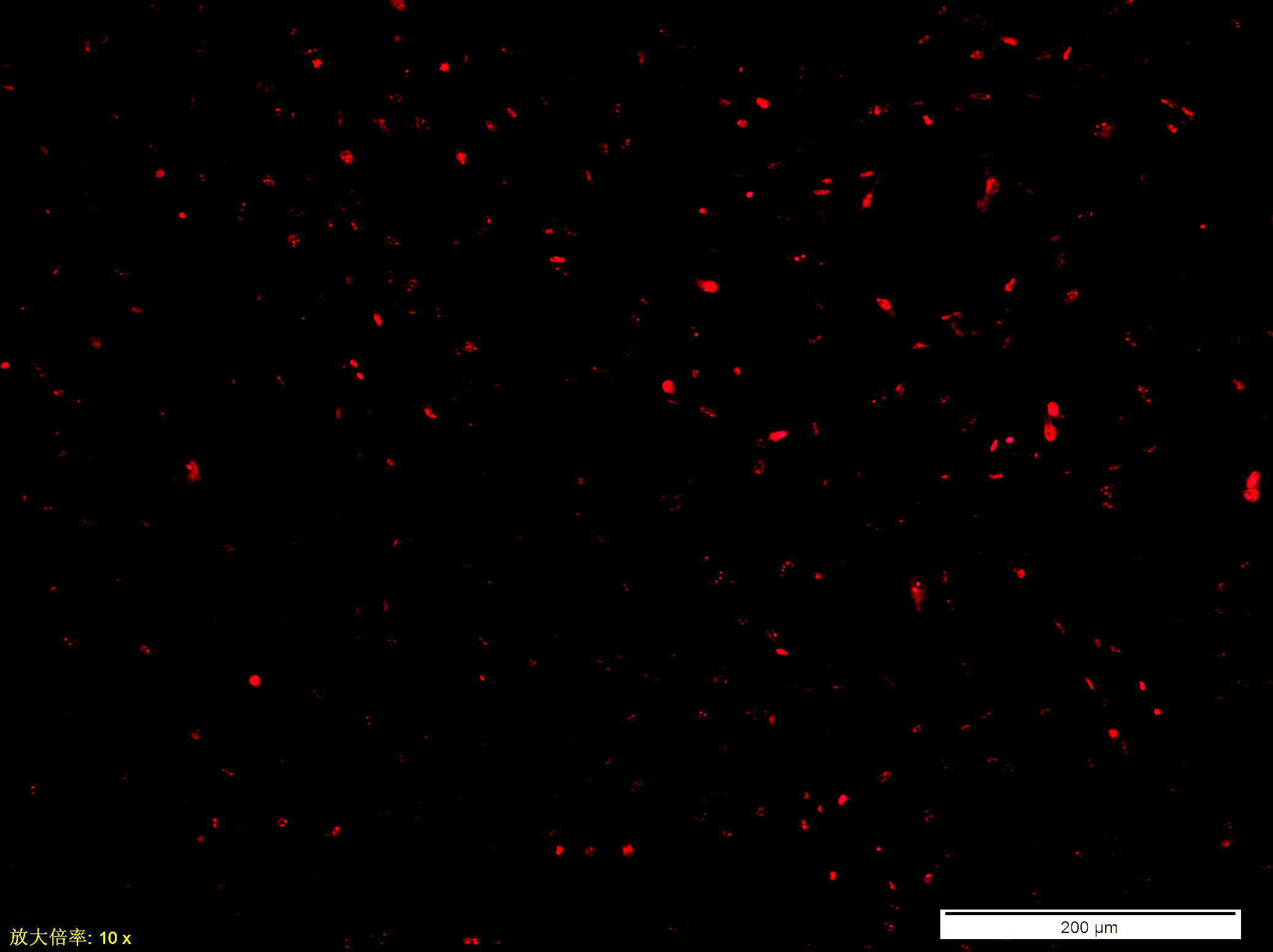

Supplement: Supplementary file 5 [file Data_Sheet_5.ZIP › Fig.11 EtBr uptake original image/LPS+TAK-242 group/Etbr (Red).jpg]

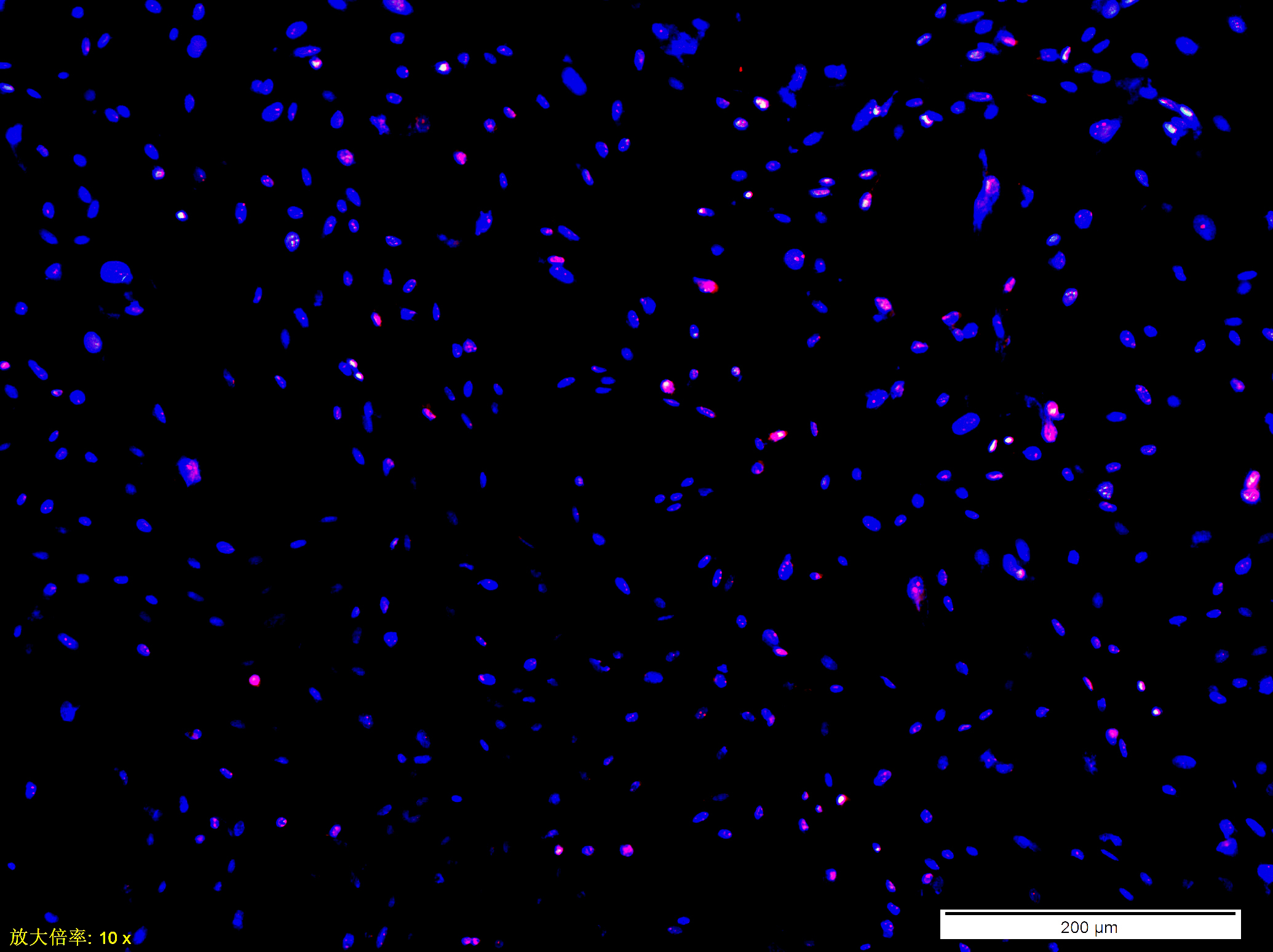

Supplement: Supplementary file 5 [file Data_Sheet_5.ZIP › Fig.11 EtBr uptake original image/LPS+TAK-242 group/merge.jpg]

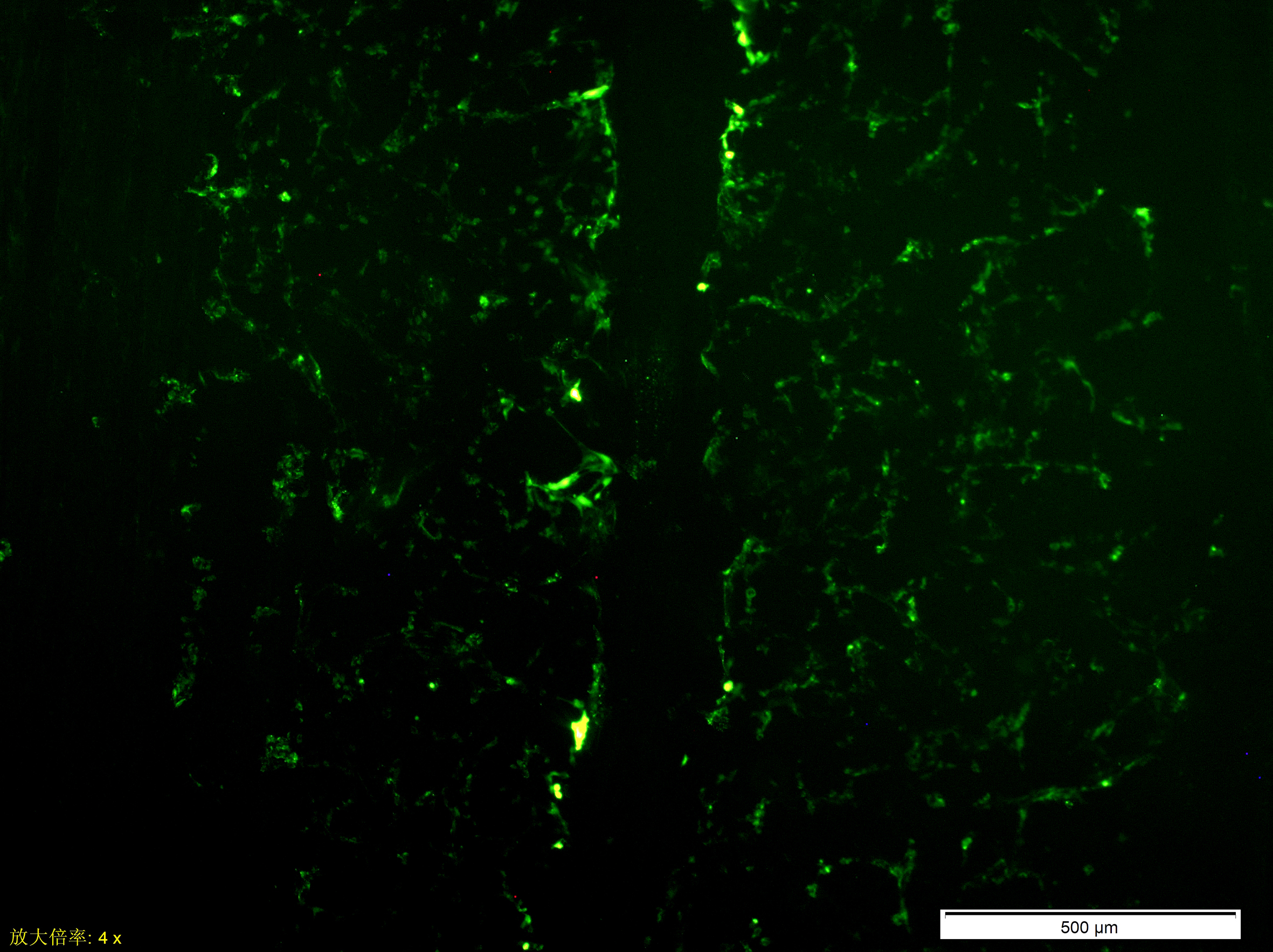

Supplement: Supplementary file 6 [file Data_Sheet_6.ZIP › Fig.11 Scrape-loading Dye Transfer assay original image/con group/10min/lucifer yellow (green).jpg]

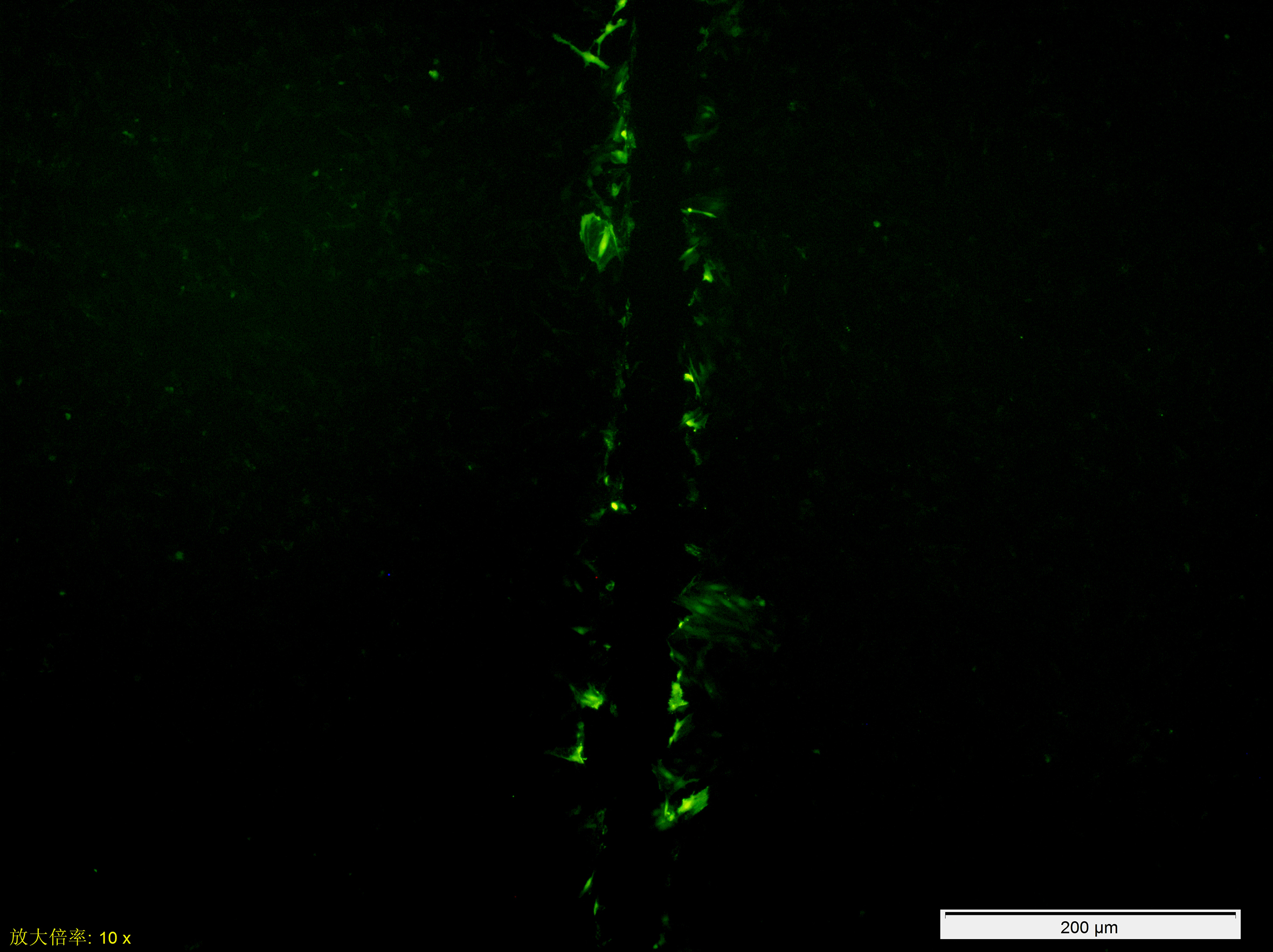

Supplement: Supplementary file 6 [file Data_Sheet_6.ZIP › Fig.11 Scrape-loading Dye Transfer assay original image/con group/5min/lucifer yellow (green).jpg]

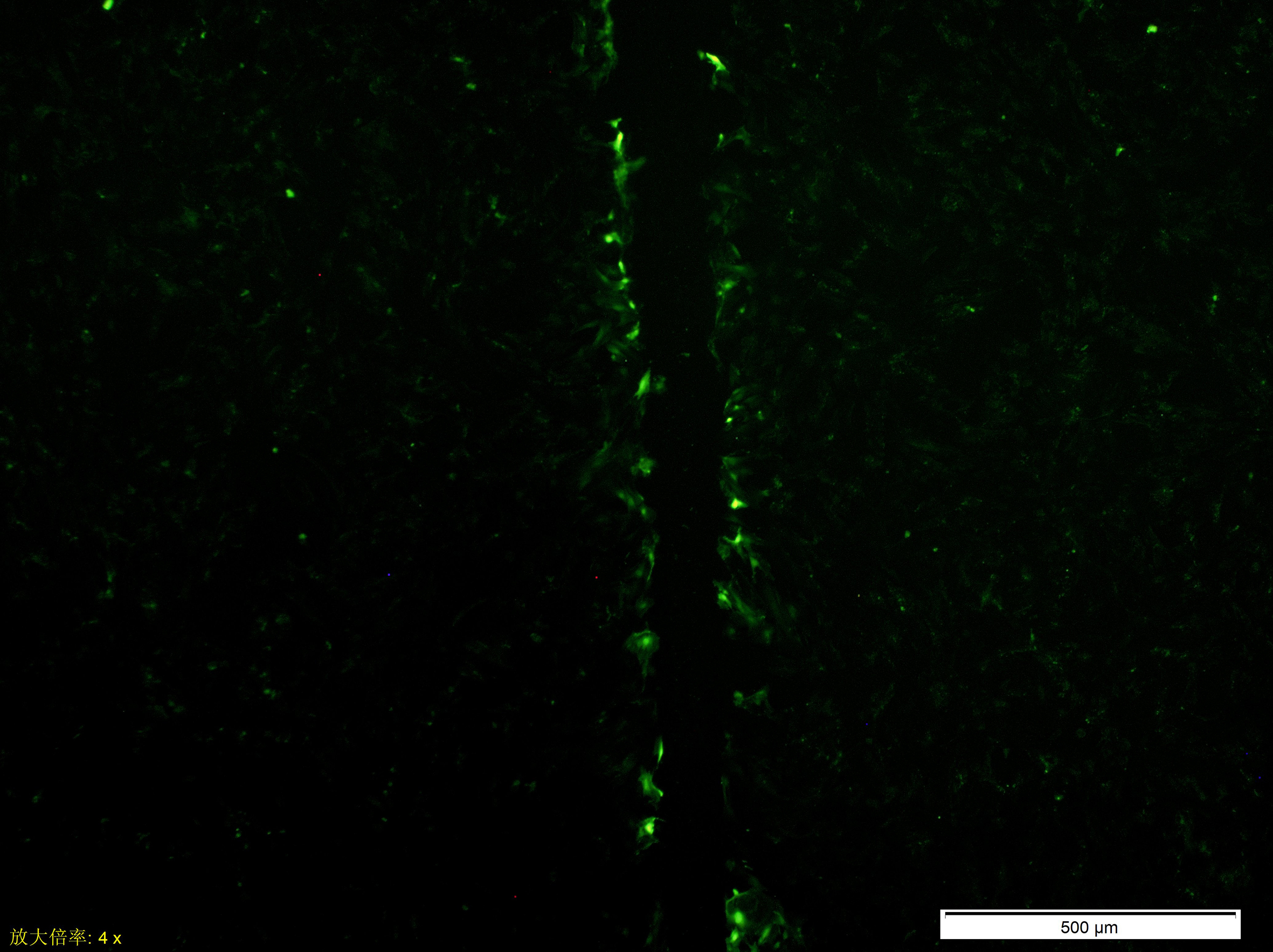

Supplement: Supplementary file 6 [file Data_Sheet_6.ZIP › Fig.11 Scrape-loading Dye Transfer assay original image/LPS group/10min/lucifer yellow (green).jpg]

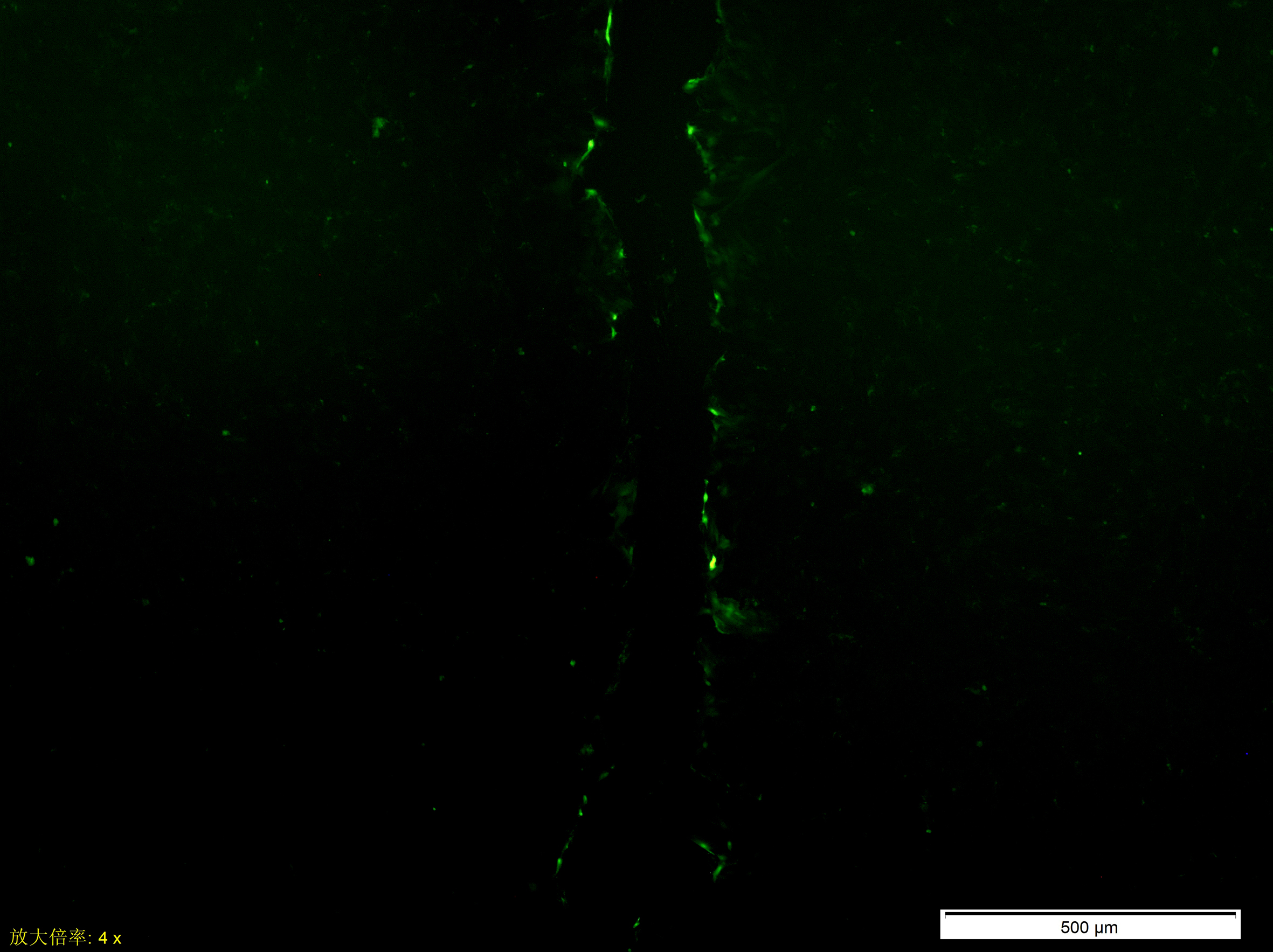

Supplement: Supplementary file 6 [file Data_Sheet_6.ZIP › Fig.11 Scrape-loading Dye Transfer assay original image/LPS group/5min/lucifer yellow (green).jpg]

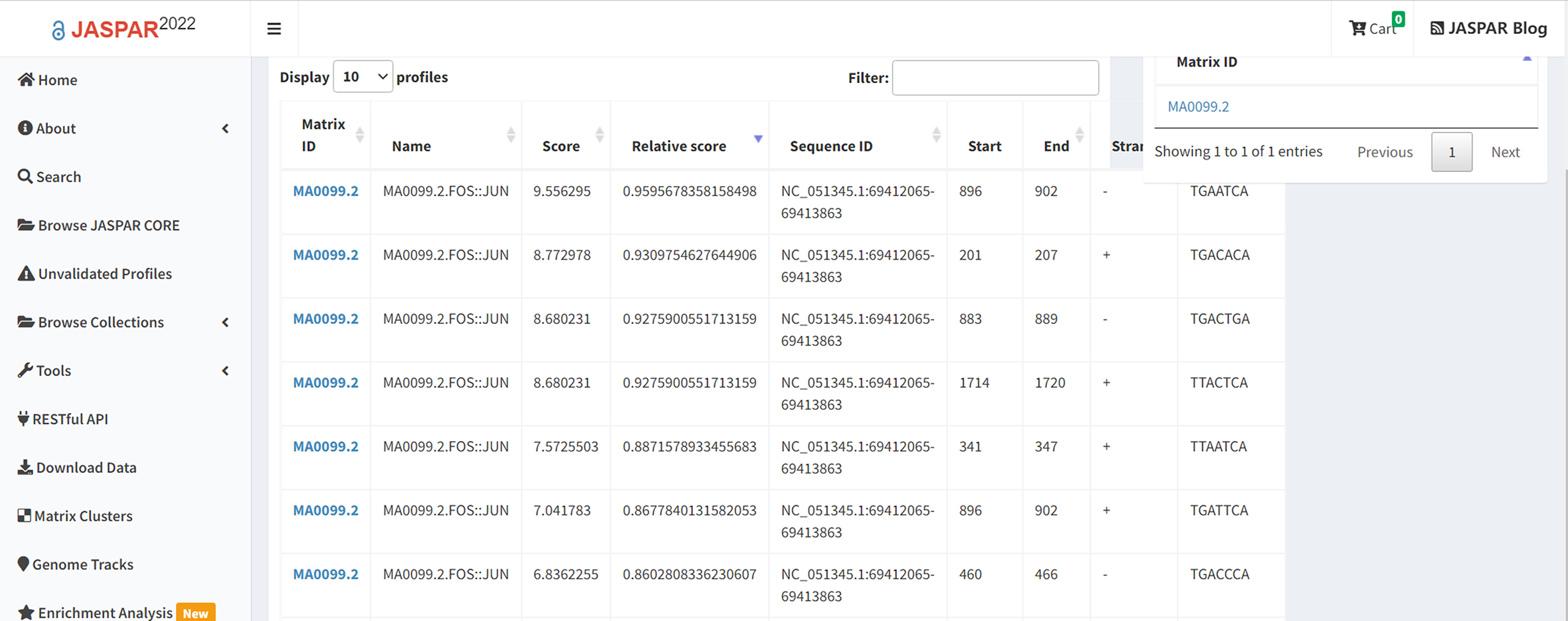

Supplement: Supplementary file 7 [file Data_Sheet_7.ZIP › JASPAR and PROMO analysis/JASPAR analysis/AP-1-2;AP-1-3;AP-1-4.jpg]

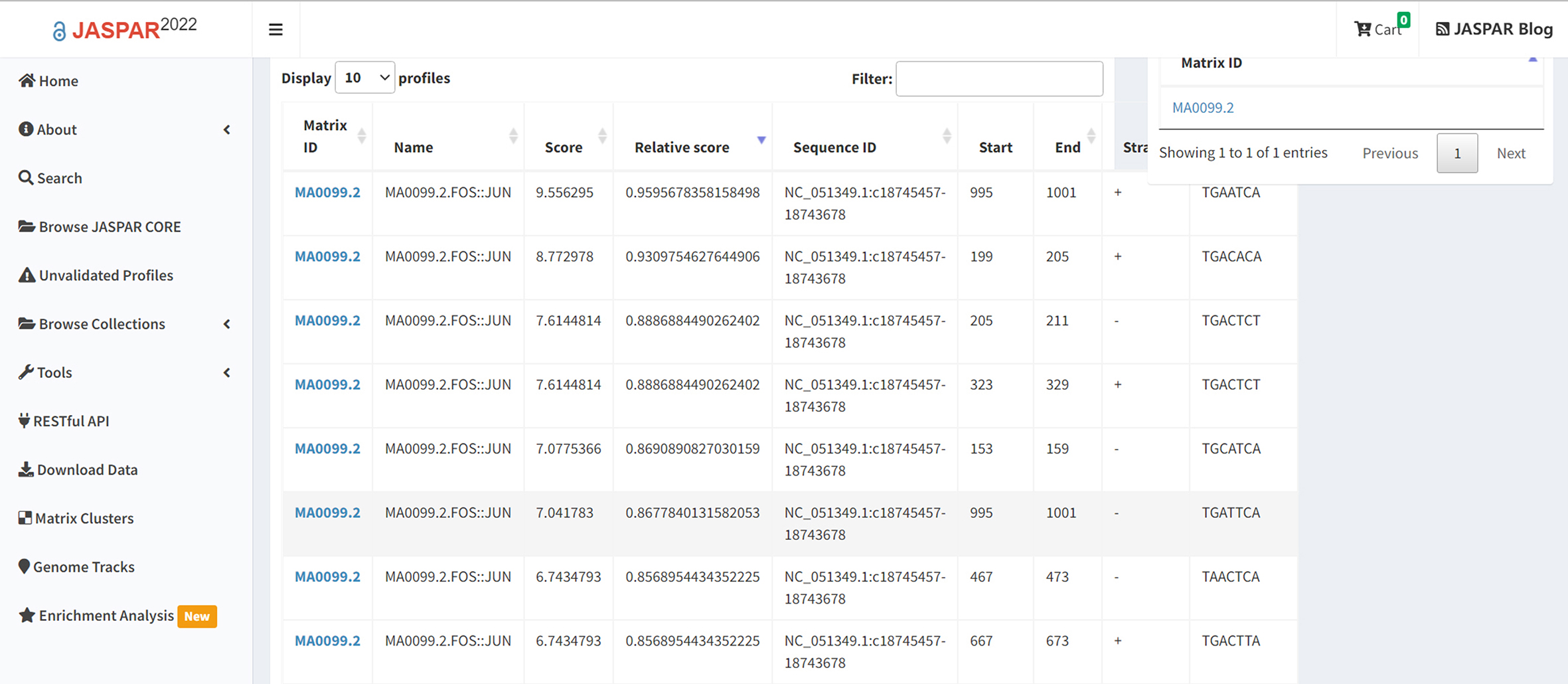

Supplement: Supplementary file 7 [file Data_Sheet_7.ZIP › JASPAR and PROMO analysis/JASPAR analysis/AP-1-5;AP-1-6;AP-1-7.jpg]

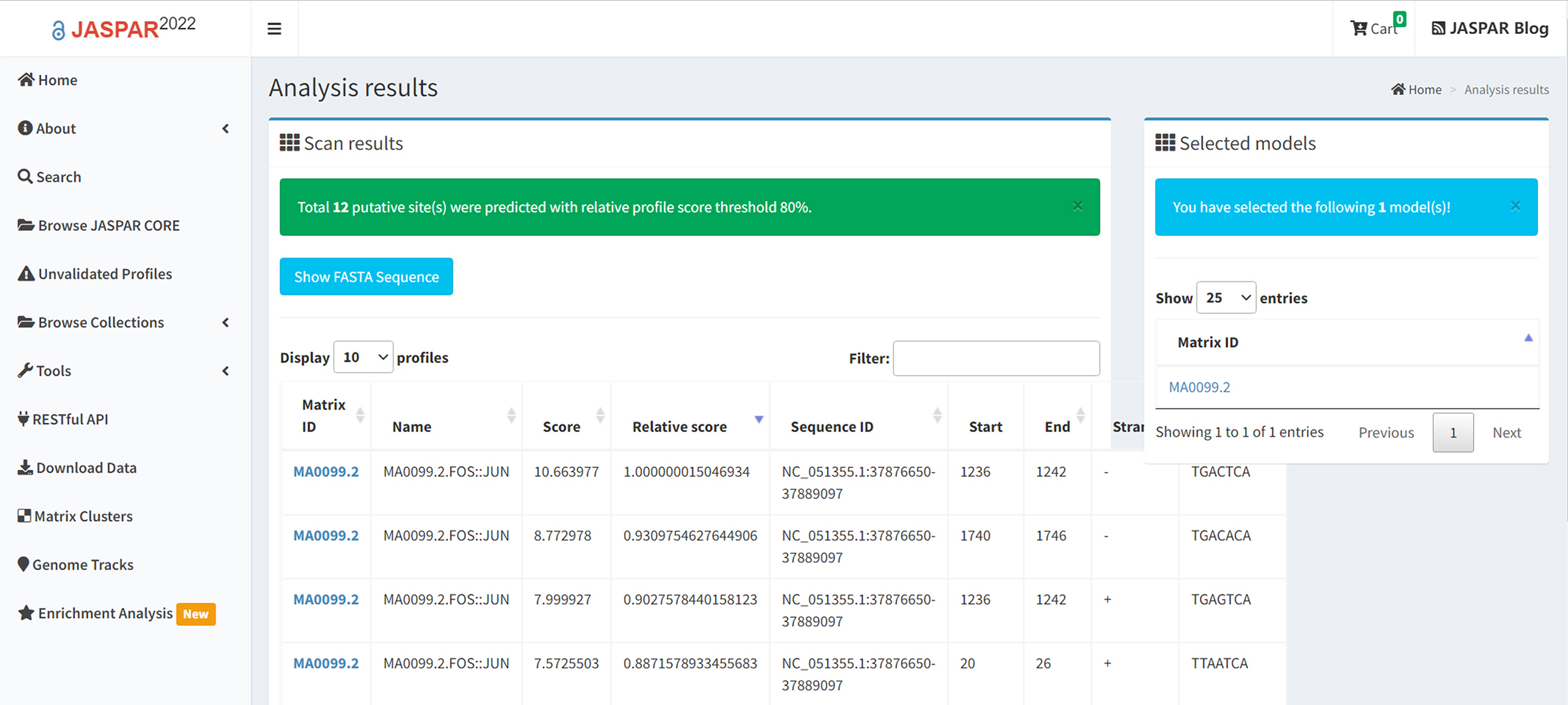

Supplement: Supplementary file 7 [file Data_Sheet_7.ZIP › JASPAR and PROMO analysis/JASPAR analysis/AP-1-8;AP-1-9.jpg]

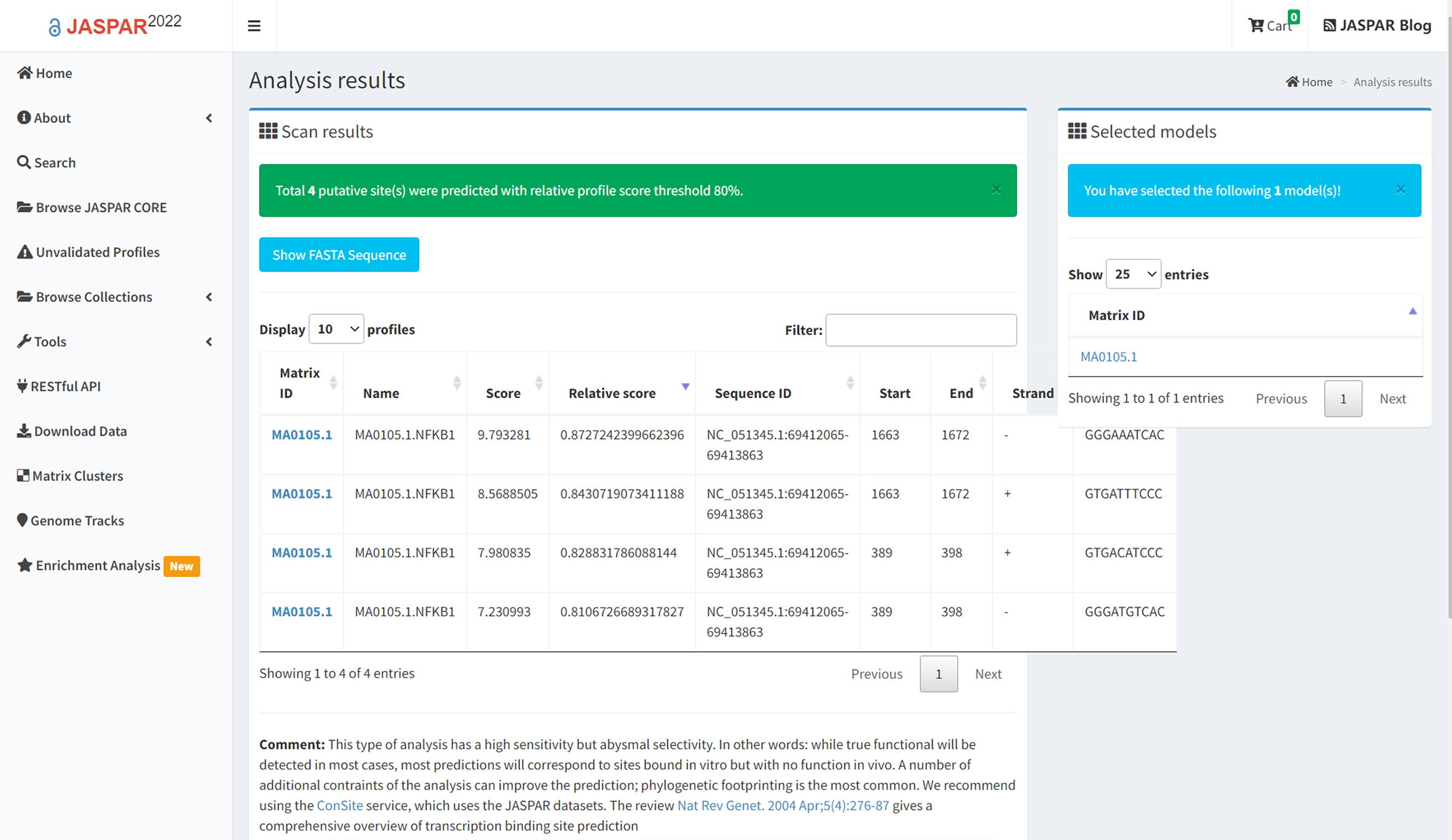

Supplement: Supplementary file 7 [file Data_Sheet_7.ZIP › JASPAR and PROMO analysis/JASPAR analysis/p50-1;p50-2.jpg]

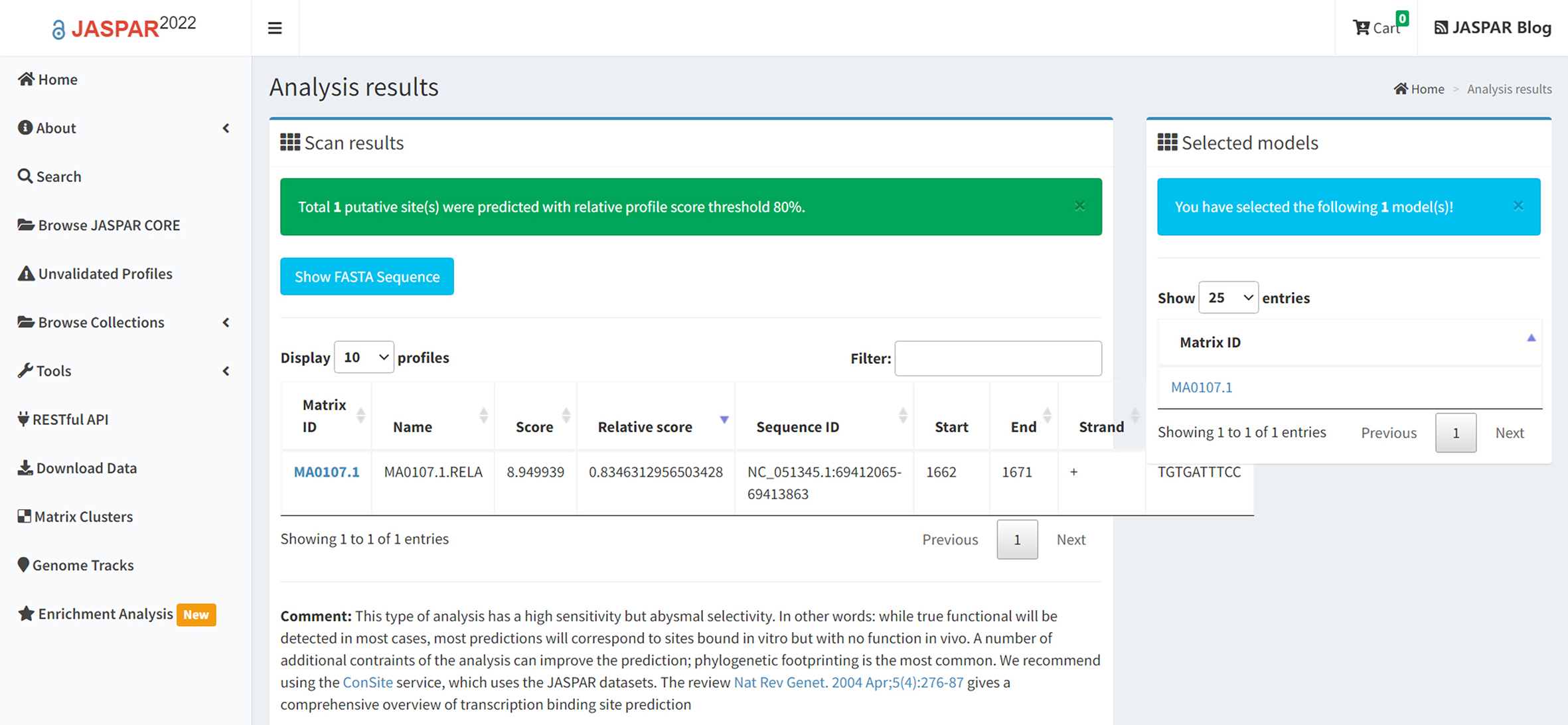

Supplement: Supplementary file 7 [file Data_Sheet_7.ZIP › JASPAR and PROMO analysis/JASPAR analysis/p65-2.jpg]

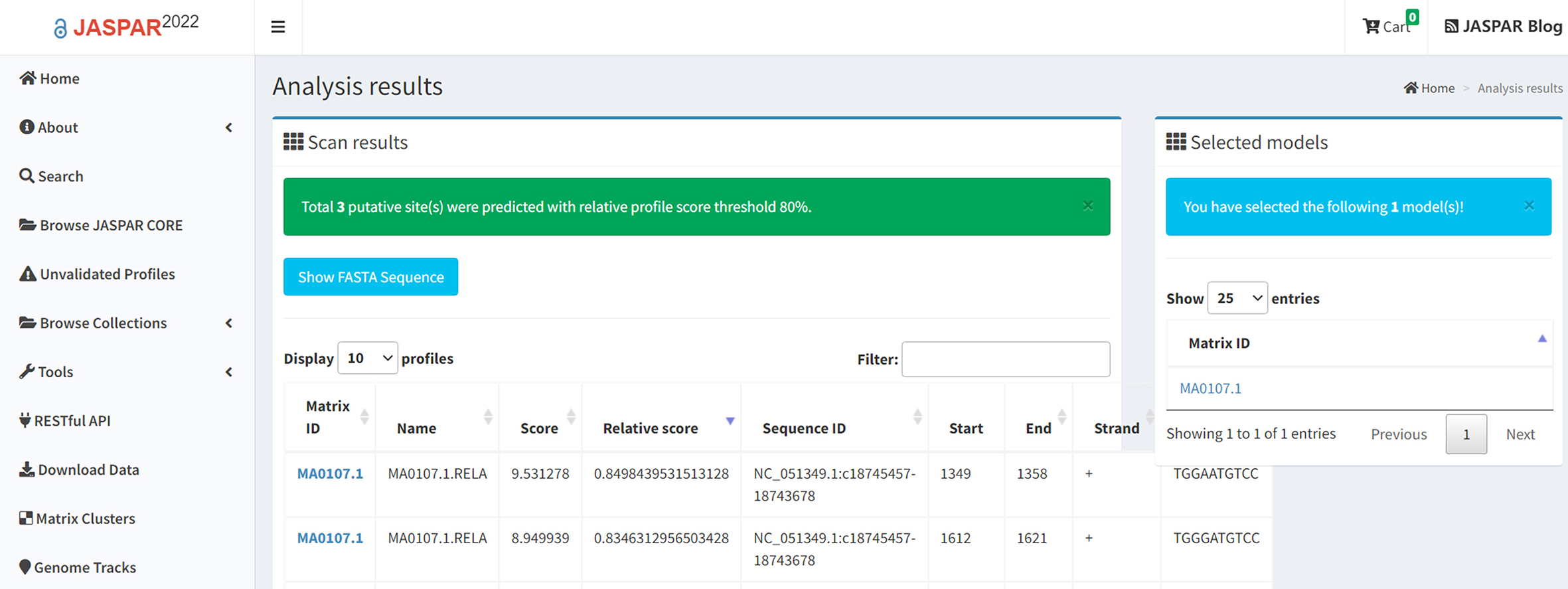

Supplement: Supplementary file 7 [file Data_Sheet_7.ZIP › JASPAR and PROMO analysis/JASPAR analysis/p65-3;p65-4.jpg]

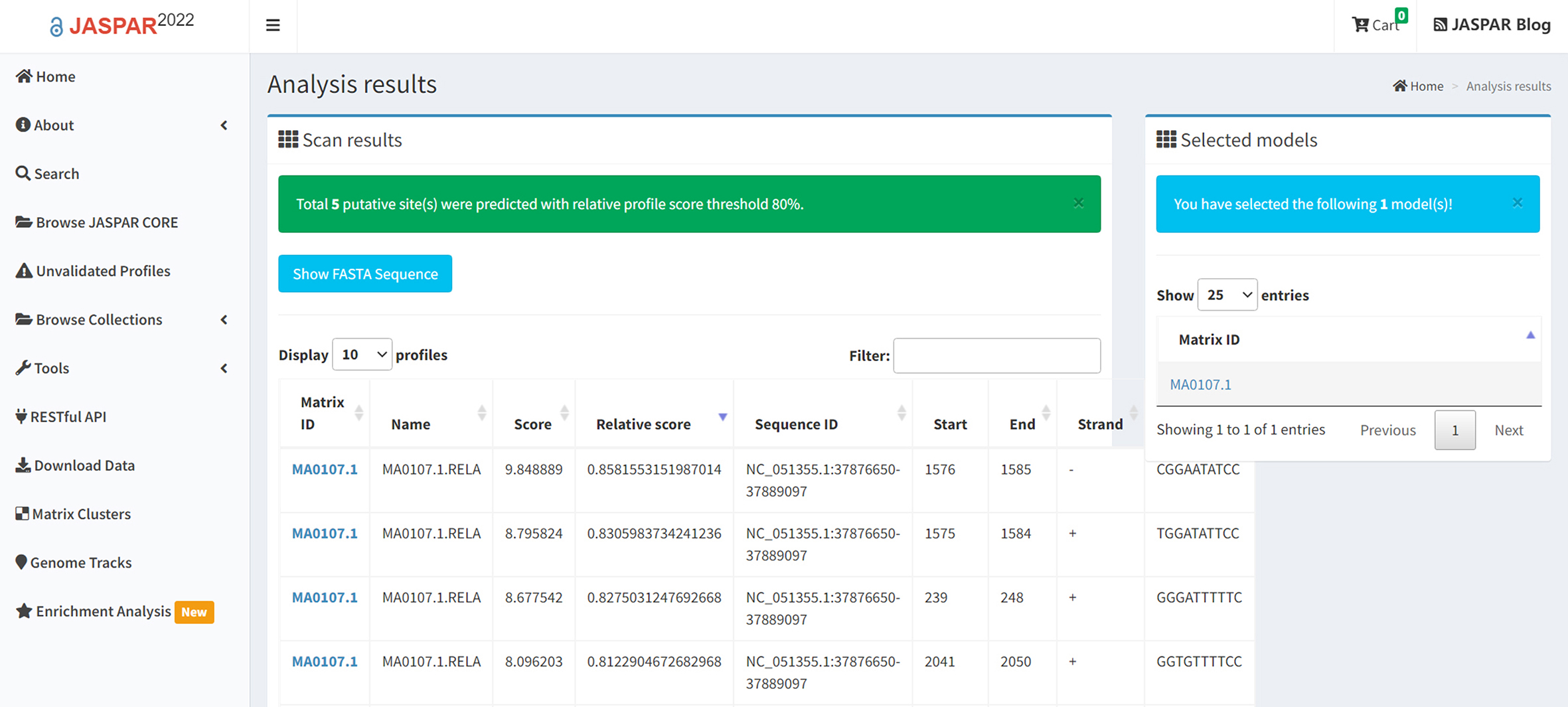

Supplement: Supplementary file 7 [file Data_Sheet_7.ZIP › JASPAR and PROMO analysis/JASPAR analysis/p65-5;p65-6;p65-7.jpg]

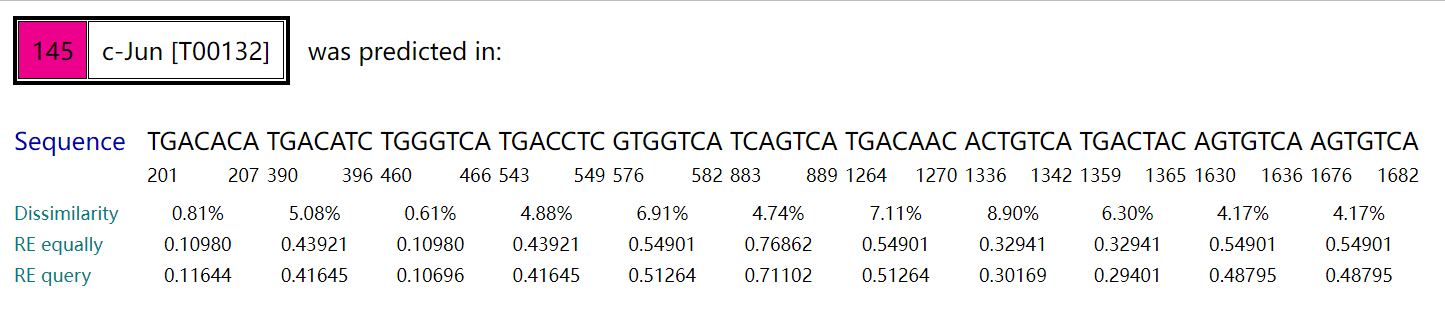

Supplement: Supplementary file 7 [file Data_Sheet_7.ZIP › JASPAR and PROMO analysis/PROMO analysis/AP-1-2;AP-1-3;AP-1-4.jpg]

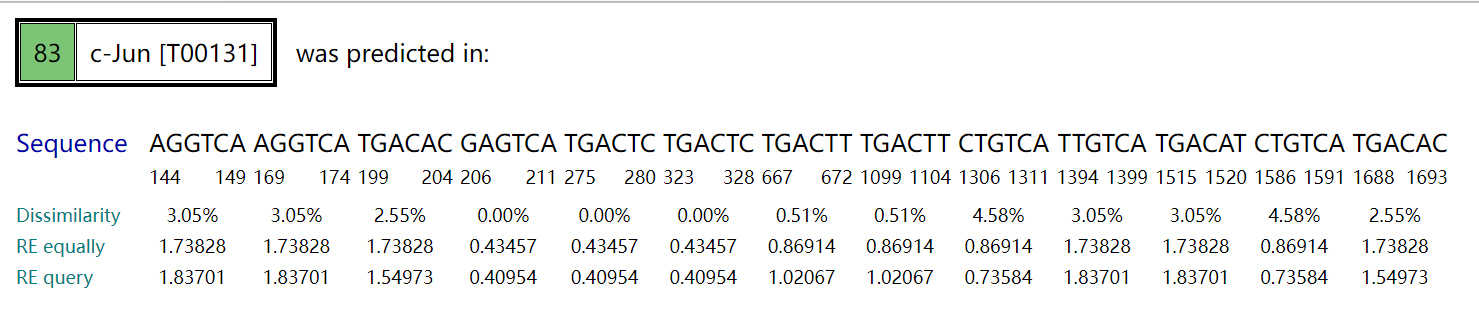

Supplement: Supplementary file 7 [file Data_Sheet_7.ZIP › JASPAR and PROMO analysis/PROMO analysis/AP-1-5;AP-1-6;AP-1-7.jpg]

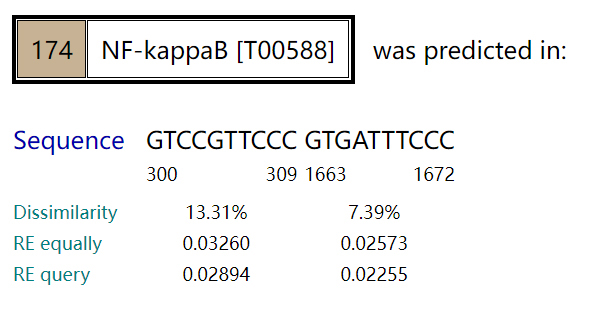

Supplement: Supplementary file 7 [file Data_Sheet_7.ZIP › JASPAR and PROMO analysis/PROMO analysis/p65-2.jpg]

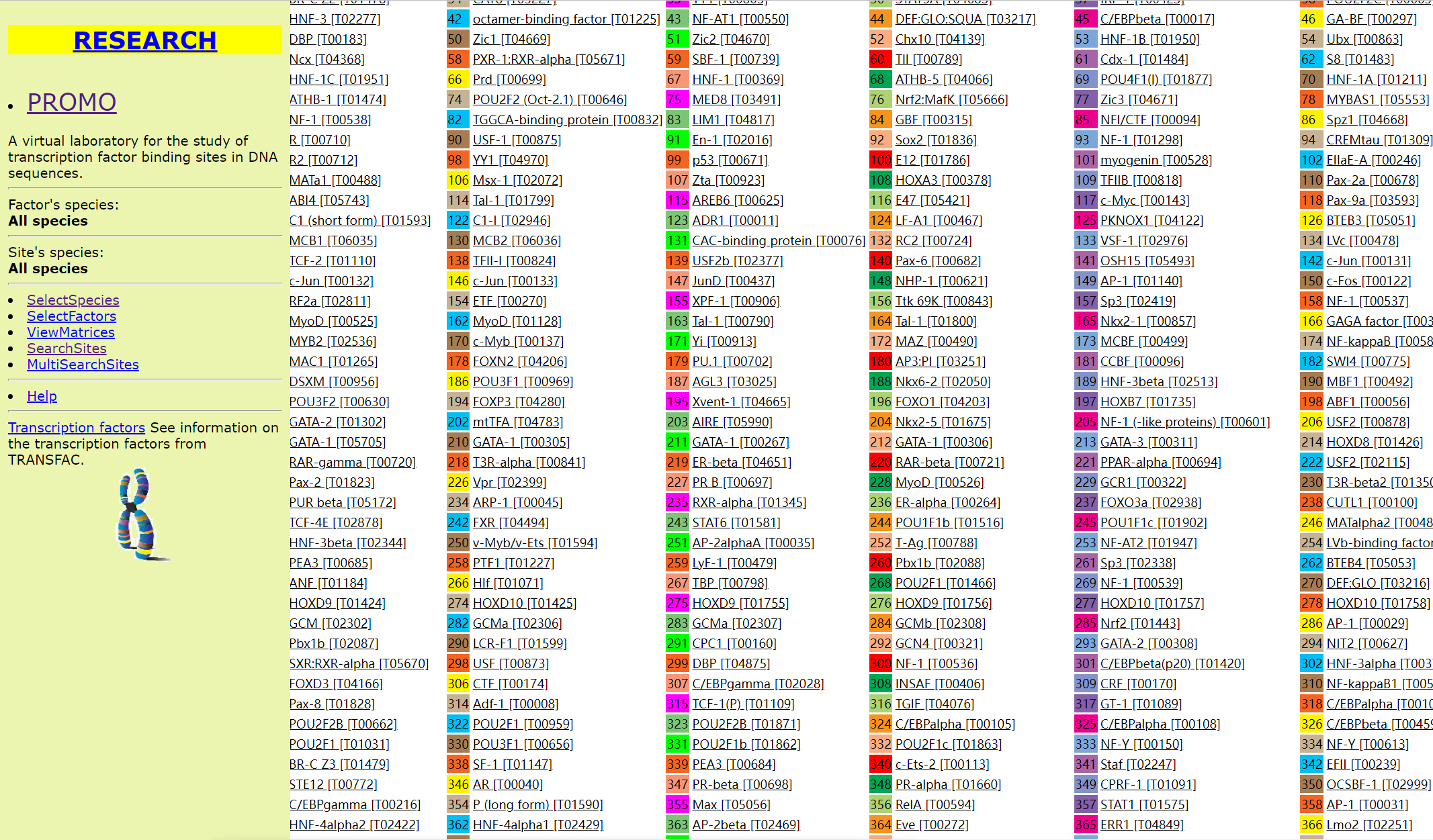

Supplement: Supplementary file 7 [file Data_Sheet_7.ZIP › JASPAR and PROMO analysis/PROMO analysis/PROMO website.jpg]
